# Intratumoral *Brevibacillus parabrevis* enhances antitumor immunity by inhibiting NK cell ferroptosis in hepatocellular carcinoma

**DOI:** 10.1038/s41419-025-07733-7

**Published:** 2025-05-21

**Authors:** Banglun Pan, Xiaoxia Zhang, Dongjie Ye, Yuxin Yao, Zhu Zhang, Yue Luo, Hao Wu, Xiaoqian Wang, Nanhong Tang

**Affiliations:** 1 Department of Hepatobiliary Surgery and Fujian Institute of Hepatobiliary Surgery, Fujian Medical University Union Hospital, Fuzhou, China; 2 Cancer Center of Fujian Medical University, Fujian Medical University Union Hospital, Fuzhou, China; 3 Key Laboratory of Ministry of Education for Gastrointestinal Cancer, Fujian Medical University, Fuzhou, China; 4 Key Laboratory of Clinical Laboratory Technology for Precision Medicine (Fujian Medical University), Fujian Province University, Fuzhou, China

**Keywords:** Experimental models of disease, Cancer microenvironment, Cell death and immune response

## Abstract

It is known that intestinal flora affects the number and function of NK cells through metabolites, thereby regulating the response of tumors to chemotherapy or immunotherapy. However, little is known about whether intratumoral bacteria are involved in NK cell-mediated antitumor immunity. In this study, 2bRAD-M analysis was performed on patient hepatocellular carcinoma and paired tissues to determine the composition of the intratumoral microbiota. Mass cytometry, flow cytometry, co-immunoprecipitation, immunoblotting, immunofluorescence, and DNA pull-down assays were used to evaluate the relationship between intratumoral bacteria, ferroptosis, and NK cell activity in Hu-SRC mice. Here, we found that the intratumoral *B. parabrevis* inhibited NK cell ferroptosis by promoting lipolysis into acetyl-CoA. Mechanistically, *B. parabrevis* catalyzed the acetylation of RORC, enhancing its binding to the NEDD4L promoter. NEDD4L induced ubiquitination of iron transporters SLC39A14, SLC39A8, and STEAP3. Functionally, *B. parabrevis* induced NK cells to differentiate into adaptability, cytotoxicity, and heat shock phenotypes, inhibiting the terminal phenotype and changing the tumor microenvironment from “cold” to “hot”. In conclusion, *B. parabrevis* enhanced the antitumor response of NK cells by regulating post-translational modifications. Our study identified a new strategy for utilizing intratumor bacteria for clinical treatment.

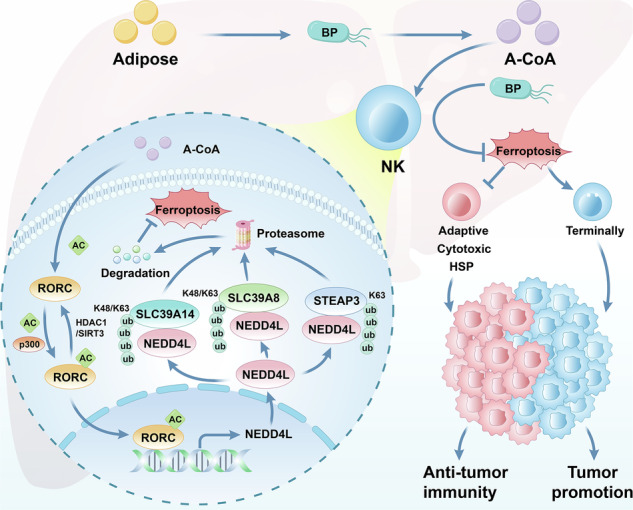

## Introduction

Ferroptosis, a distinct form of programmed cell death reliant on iron ions, is initiated by the accumulation of lipid peroxides and differs from other cell death modalities such as apoptosis and necrosis [1]. Ferroptosis plays a pivotal role in tumor immunity by enhancing anti-tumor responses through the release of damage-associated molecular patterns, activation of dendritic cells, and promotion of T-cell-mediated anti-tumor activity [2]. Additionally, ferroptosis influences the polarization of tumor-associated macrophages, facilitating their shift from the M2 phenotype to the M1 phenotype [3]. Furthermore, ferroptosis has the potential to mitigate immunosuppression by reducing the number and function of regulatory T cells [3]. Therefore, the combination of ferroptosis-targeting compounds has demonstrated significant efficacy; specifically, the integration of ferroptosis agonists with PD-1/PD-L1 inhibitors enhances anti-tumor effects, and when combined with chemotherapy or radiotherapy, it synergistically eradicates tumor cells through diverse mechanisms [4]. Nonetheless, the selective inhibition of ferroptosis in immune cells remains an area requiring further investigation. Certain immune cells or tumor cells may exhibit resistance to ferroptosis, necessitating a deeper exploration of the underlying mechanisms. Consequently, ferroptosis is pivotal in tumor immunity, offering a novel avenue for cancer therapy by augmenting anti-tumor immunity and mitigating immunosuppression.

There are a large number of microorganisms in tumor tissues, some of which change the tumor microenvironment (TME) and participate in the occurrence and development of tumors: causing DNA damage through metabolites and secretions, participating in signal transduction of signaling pathways, and affecting immune responses [5]. However, little is known about whether there are intratumoral bacteria that enhance the function of cytotoxic immune cells, and whether the effect of immunotherapy can be improved by transplanting specific bacterial flora needs to be clarified.

In this study, we took the intratumoral *Brevibacillus parabrevis* (*B. parabrevis*) as an entry point to investigate its role in NK cell-mediated immune responses: (1) First, we found that *B. parabrevis* upregulated the expression of NEDD4L, which inhibited NK cell ferroptosis by promoting the ubiquitination of iron transporters SLC39A14, SLC39A8, and STEAP3. (2) We pointed out that *B. parabrevis* inhibited ferroptosis of NK cells, promoted their transformation to cytotoxic/adaptive/heat shock phenotypes, inhibited their terminal differentiation, and enhanced the killing of hepatocellular carcinoma (HCC) cells. (3) Finally, we indicated that *B. parabrevis* promoted lipolysis, produced acetyl-CoA, increased RORC acetylation, enhanced the binding of RORC to the NEDD4L promoter, and upregulated its transcription. Therefore, *B. parabrevis* was leveraged as a novel adjuvant to improve the antitumor response of NK cells in HCC.

## Methods and materials

### Mouse model

10-week-old female mice weighing 20–25 g (C57BL/6J or Human SCID Repopulating cell (Hu-SRC) mice) were purchased from Shanghai Model Organisms Center, Inc (China). All mice were housed in individually ventilated cages in a specific pathogen-free environment at room temperature at Fujian Medical University Laboratory Animal Center and relative humidity of 50–60%. Six mice were randomly assigned to each group. Considering the effect of intestinal flora on the liver microenvironment and the interaction between various immune cells and NK cells, we constructed an orthotopic HCC model of humanized immunized mouse Hu-SRC and transplanted *B. parabrevis* or NK cells to accurately assess the role of human genes in NK cells on the HCC immune microenvironment. For C57BL/6J mice, the mouse HCC cell line Hep-53.4 was applied, while the Hu-SRC mice were inoculated with patient-derived tumor tissue cells. In this study, we mainly used the orthotopic HCC model to evaluate the impact of *B. parabrevis* and NK cells on the TME, which was corroborated by the subcutaneous tumor model.

1. Preparation of patient-derived tumor tissue cells: Tumor specimens were obtained (approximately 2 × 2 × 2 mm^3^/fragment) from one HCC patient (Table S1). The tumor mass was cut into small pieces with a diameter of 1–3 mm and digested in tissue digestion solution [0.4 mg/ml Collagenase Type IV (#17104019, Gibco, NY, USA), 10 mg/ml DNase I (#18047019, Invitrogen, CA, USA), 10% FBS (TMS-016, Merck, MA, USA), DPBS (#D5773, Merck)] at 37 °C for 30 min. The reaction was terminated by adding EDTA (#17892, Thermo Scientific, CA, USA) to a final concentration of 5 mM. To make the sample uniform, pipette repeatedly with a 23 G needle until a homogenate was formed. The homogenate was passed through a 70 mm cell strainer and centrifuged at 400 × *g* for 8 min at 4 °C. Tumor tissue cells were plated on bottles at 37 °C in Human Liver Organoid Culture Medium (#abs9529, Absin, China) for up to two weeks. Organoids with a diameter of 300 µm were made with DPBS at a concentration of 2 × 10^7^/ml, 1 × 10^6^ cells per Hu-SRC mouse, and an orthotopic liver injection volume of 100 μl. Passage 1 were maintained and sacrificed when the mice lost their weight and appetite. Organoids (passage 1) were prepared from minced xenograft in the same manner as patient tissues and implanted for several generations. Mouse model of carcinoma in situ used for formal experiments was established at passage 3, when tumor phenotype tended to stabilize.
2. Construction of orthotopic tumor model: Hep-53.4 cell (#CVCL_5765, Cellosaurus, Switzerland) or patient-derived tumor tissue cell suspensions were made with sterile DPBS at a concentration of 2 × 10^7^/ml, 1 × 10^6^ cells per mouse, and an injection volume of 50 μl. Mice were anesthetized with 0.8% Pentobarbital Sodium (#P3761, Merck), 60 mg/kg, by intraperitoneal injection. After anesthesia, the mice were fixed on the operating board, and the surgical site was disinfected. A longitudinal incision was made about 1 cm below the xiphoid process of mice to open the abdominal cavity, and the left lobe of the liver was gently exposed with a sterile cotton swab. Fix the liver lobe with a cotton swab, insert a 1 ml micro-syringe along the liver surface at 15–30°, penetrate into the liver about 0.5 cm, inject the cell suspension slowly, withdraw the needle slowly, and use a sterile cotton swab to lightly press the injection site to stop bleeding. The liver was then carefully placed back into the abdominal cavity, the abdomen was sutured layer by layer, and the incision was sterilized. Place the mice on an electric blanket until the mice wake up, then return them to their cages and observe the changes in the mice’s vital signs and body weight. Five days after the cell injection, the mice were randomly divided into control and experimental groups, and subsequent experiments were carried out. The orthotopic tumorigenesis time of mouse HCC is about three weeks. Anesthesia and euthanasia during the experiment: Animal ethics stipulate that if an animal becomes listless and loses its appetite, it should be immediately euthanized with CO_2_. If the animal survives for more than three months, it should also be euthanized to avoid unnecessary suffering.
3. To construct a subcutaneous tumor model, Hep-53.4 cells or patient-derived tumor tissue cells (5 × 10^6^) were resuspended in 100 μl DPBS and injected subcutaneously into anesthetized mice. Tumor volume was recorded using a vernier caliper (tumor volume = length × width^2^/2). When the tumor volume reached 100 mm^3^, the mice were randomly divided into different groups. The food intake and body weight of tumor-bearing mice were monitored regularly. Anesthesia and euthanasia during the experiment: Animal ethics stipulate that the weight of mouse tumors should not exceed 10% of the mouse body weight, the average tumor diameter should not exceed 20 mm, and if ulceration, infection or necrosis occurs, the experiment should be terminated, and the animal should be euthanized with CO_2_. The tumor was removed, photographed, and weighed.
4. Bacterial transplantation: To ensure that the mouse tumor model accurately represented the *B. parabrevis* content observed in patient HCC tumors, we initially quantified the bacterial presence in patient tumor tissues using fluorescence in situ hybridization (FISH) assay. Subsequently, we eliminated the endogenous bacteria in the mice using an antibiotic regimen to prevent confounding variables. Then, we administered a gradient concentration of *B. parabrevis* suspension, calibrated to mimic the bacterial load found in patient HCC tissues, either through gavage for carcinoma in situ or via intratumoral injection for subcutaneous tumors [6, 7]. Briefly, (I) To eliminate the bacterial flora in mice, mice were orally fed with antibiotic premix (1 ml/kg/d, Metronidazole (1 mg/ml, #M1547, Merck), Ampicillin (1 mg/ml, #A5354, Merck), Neomycin (1 mg/ml, #N1142, Merck), Gentamicin (1 mg/ml, #G1914, Merck), and Vancomycin (0.5 mg/ml, #V2002, Merck)) daily, and antibiotic premix (50 μl/ml) was added to the drinking water for 16 consecutive days. Hep-53.4 cells or patient-derived tumor tissue cells were administered one week after completion of antibiotic treatment. (II) Bacterial inoculation in orthotopic tumor models: *B. parabrevis* was gavaged into microbiota-depleted mice (200 μl per mouse (5 × 10^6^ CFU)) 1 days after the tumor cells were injected, and then every day for a total of five doses. (III) Bacterial inoculation in subcutaneous tumor model: *B. parabrevis* (with a load of 5 × 10^5^ CFU/mouse in 50 μl DPBS) was injected into the tumor mass 3 days after the tumor cells were injected, and then every day for a total of five doses.
5. NK cell elimination and infusion. (I) Elimination: Mice were intraperitoneally injected with InVivoMAb anti-mouse NK1.1 (10 mg/kg/d, # BE0036, Bio X Cell, NH, USA) 3 days after the tumor cells were injected, and then every day for a total of five times. (II) Infusion: Resuspend 5 × 10^6^ NK-92 cells in 100 μl normal saline and inject into the tail vein 10 days before the tumor cells were injected, and once every day for a total of five times.
6. Drug treatment: The drug injection protocol involved administering Liproxstatin-1 (15 mg/kg, HY-12726, MedChemExpress, NJ, USA) into the tail vein 10 days before the tumor cells were injected, and then every day for a total of five doses. Equal volume of DMSO (HY-Y0320, MedChemExpress) was used as the control group.

### Statistical analysis

We used SPSS 19.0 (IBM, NY, USA) and R 4.4 (Lucent Technologies, NJ, USA) software for statistical analysis, and Prism 9.0 (GraphPad, CA, USA) and R 4.4 software to generate visual images. All data were expressed as mean ± SD, and reported *P* values less than 0.05 were considered statistically significant and shown on the corresponding graphs. When comparing two samples, the independent sample *t* test was used if the data were normally distributed and the variances were homogeneous. Otherwise, Wilcoxon rank sum test was used. Kaplan-Meier curves with 95% confidence intervals were plotted and the Log-rank test was used to compare survival curves.

Other detailed methods are listed in Supplemental Information.

## Result

### Characteristics of intratumoral bacterium in patient HCC

Based on our previous demonstration that intestinal flora regulates the infiltration and function of HCC-infiltrating T and NK cells [6], we further explored the relationship between intratumoral bacterium and immune cells. We found that NK cell infiltration was impaired in patient HCC tissues (Fig. S1A), so we selected 10 pairs of patient cancer and paired tissues for 2bRAD-M analysis [8] to determine which bacterium regulates NK cell antitumor immunity. Principal co-ordinates analysis revealed a clear separation of microbiota composition between cancer and paired tissues (Fig. S1B). KEGG enrichment analysis showed that the microbial community profiles in HCC tissues were associated with impaired metabolic pathways compared with paired tissues (Fig. S1C), and K02015 (iron complex transport system permease protein) had a lower score in HCC tissues than in paired tissues (Fig. S1D). Since we detected only one fungus, *Malassezia restricta*, and its contribution was negligible, we next analyzed the specific bacterial composition. Quantitative analysis of bacterial abundance revealed that at the species level, the absolute abundances of *Anoxybacillus A rupiensis*, *Bradyrhizobium sp003020075*, *B. parabrevis*, *Methylobacterium rhodesianum*, and *Ralstonia sp021173085* were lower in HCC tissues (Fig. S1E). So, we next evaluated which of these five bacteria had immunomodulatory effects.

To ascertain the generalizability of the arguments derived from our samples to a broader population, we employed the BIC dataset [9] from the TCGA database for external validation. The bacterial reads determined from the BIC dataset [9] showed that the abundance of *Brevibacillus* in patient HCC tissues was lower than that in paired tissues at the genus level, while there was no difference in *Anoxybacillus*, *Bradyrhizobium*, *Methylobacterium*, and *Ralstonia* (Fig. S1F). To characterize the relationship between *Brevibacillus* and the TME of HCC, we imported the transcriptional profile data of TCGA into CIBERSORT [10] to calculate the proportion of immune cells and found that the proportion of activated NK cells in the high *Brevibacillus* group was higher than that in the low one (Fig. S1G). We subsequently performed FISH assay to investigate the presence of *B. parabrevis* in patient HCC and paired tissues. Our findings confirmed the presence of lower levels of *B. parabrevis* in HCC tissues (Fig. S1H). We further evaluated the relationship between *B. parabrevis* content and NK cell function and found that in comparison to the low infection group, patients in the high infection group exhibited elevated expressions of cytotoxic markers [11], including FCER1G, GZMK, IFN-γ, and TNFRSF18, heat shock protein HSPA1B [11], and the adaptive marker NKG7 [11] in tumor-infiltrating NK cells (Fig. S1I, J). To precisely replicate the extent of *B. parabrevis* present in patient HCC tissues (Fig. S1H), we conducted an experiment involving tumor-bearing mice, which were infected with varying concentrations of *B. parabrevis*. Our findings indicated that administering 5 × 10^6^ CFU of *B. parabrevis* orally to mice with orthotopic tumors or delivering an intratumoral injection of 5 × 10^5^ CFU of bacteria to mice with subcutaneous tumors, was appropriate (Fig. S1K). What’s more, *B. parabrevis* was witnessed to facilitate the intratumoral invasion of NK cells, both in in situ carcinoma models and subcutaneous tumor models (Fig. S1L).

### *B. parabrevis* enhanced the antitumor activity of NK cells

**Fig. 1:**
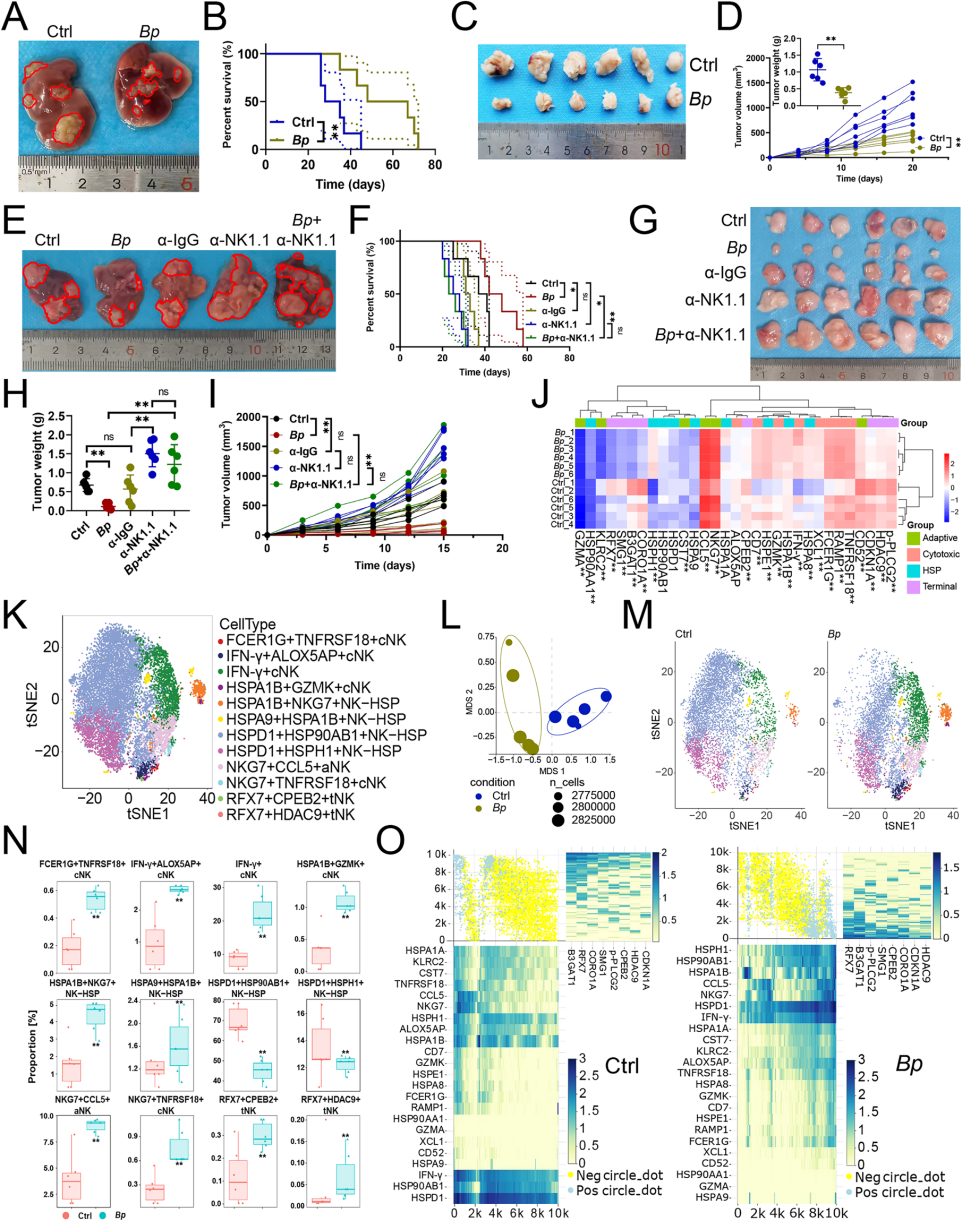
*B. parabrevis* enhanced the antitumor activity of NK cells. **A**, **B** Effects of *B. parabrevis* transplantation on orthotopic HCC growth in C57BL/6 J mice (*n* = 6). **A** Representative. **B** Survival curve. Effects of *B. parabrevis* transplantation on subcutaneous tumor growth in C57BL/6 J mice (*n* = 6). **C** Representative. **D** Tumor weight and growth curve. **E**, **F** Effects of *B. parabrevis* transplantation on orthotopic HCC growth in NK cell-deficient C57BL/6 J mice (*n* = 6). Anti-NK1.1 antibody was injected into the tail vein to clear NK cells. **E** Representative. **F** Survival curve. **G**–**I** Effects of *B. parabrevis* transplantation on subcutaneous tumor growth in NK cell-deficient C57BL/6 J mice (*n* = 6). **G** Representative. **H** Tumor weight. **I** Tumor growth curve. **J** Heatmap showing median expression levels of NK cell characteristic antigens (*n* = 6). **K** SOM was overlaid on mass cytometry data of HCC-infiltrating NK cells (*n* = 6). **L** NMDS analysis comparing the similarity of expression levels of NK cell characteristic antigens before and after *B. parabrevis* transplantation (*n* = 6). **M** Mosaic plot of individual NK cells with and without *B. parabrevis* transplantation (*n* = 6). **N** Boxplot showing the proportion of NK cell subsets before and after *B. parabrevis* transplantation (*n* = 6). **O** One-SENSE analysis compared the terminal and functional profiles of NK cells (*n* = 6). **B**, **F** were analyzed by Log-rank test, **D**, **H**, **I**, **N** represented mean ± SD analyzed by unpaired *t* test, **J** were analyzed by Euclidean distance. *P < 0.05, **P < 0.01. NMDS, non-metric multidimensional scaling, one-SENSE, one-dimensional soli-expression by nonlinear stochastic embedding.

To study the specific function and molecular mechanism of *B. parabrevis* on NK cell-mediated anti-tumor immunity, we administered bacterial to mice with HCC and employed mass cytometry to detect specific changes in NK cells. In vivo experiments found that *B. parabrevis* inhibited the growth of orthotopic and subcutaneous tumors in mice and improved survival time (Fig. 1A–D). Our study demonstrated that the in vivo depletion of NK cells using anti-NK1.1 antibody significantly facilitated tumor progression and adversely affected prognosis (Fig. 1E–I). Furthermore, the anticancer efficacy of *B. parabrevis* was abrogated in the absence of NK cells (Fig. 1E–I), indicating that the tumor-inhibitory effects of *B. parabrevis* were contingent upon the presence of NK cells. We selected cytotoxic, adaptive, heat shock, and terminal markers to define NK cell function [11] and found that *B. parabrevis* downregulated the expression of terminal markers (p-PLCG2, HDAC9, CDKN1A, CPEB2, CORO1A, B3GAT1, SMG1, and RFX7) and upregulated the expression of adaptive markers (CD52, NKG7, and CCL5), cytotoxic markers (TNFRSF18, RAMP1, FCER1G, XCL1, IFN-γ, GZMK, and CD7), and heat shock markers (HSPA8, HSPA1B, HSPE1, HSPH1, and HSP90AA1) (Figs. 1J and S2A, B).

Next, we used the heatmap of unsupervised hierarchical clustering to visualize the antigen expression of NK cells and revealed the existence of 12 NK cell subsets (Fig. S2C). We defined them as: FCER1G^+^ TNFRSF18^+^ cytotoxic NK (cNK), IFN-γ^+^ ALOX5AP^+^ cNK, IFN-γ^+^ cNK, HSPA1B^+^ GZMK^+^ cNK, HSPA1B^+^ NKG7^+^ heat shock protein NK (NK-HSP), HSPA9^+^ HSPA1B^+^ NK-HSP, HSPD1^+^ HSP90AB1^+^ NK-HSP, HSPD1^+^ HSPH1^+^ NK-HSP, NKG7^+^ CCL5^+^ adaptive NK (aNK), NKG7^+^ TNFRSF18^+^ cNK, RFX7^+^ CPEB2^+^ terminal NK (tNK), and RFX7^+^ HDAC9^+^ tNK (Fig. 1K). Non-metric MultiDimensional Scaling (NMDS) analysis revealed that *B. parabrevis* significantly altered the function of NK cells (Fig. 1L). After *B. parabrevis* transplantation, the proportions of FCER1G^+^ TNFRSF18^+^ cNK, IFN-γ^+^ ALOX5AP^+^ cNK, IFN-γ^+^ cNK, HSPA1B^+^ GZMK^+^ cNK, HSPA1B^+^ NKG7^+^ NK-HSP, HSPA9^+^ HSPA1B^+^ NK-HSP, NKG7^+^ CCL5^+^ aNK, NKG7^+^ TNFRSF18^+^ cNK, RFX7^+^ CPEB2^+^ tNK, and RFX7^+^ HDAC9^+^ tNK increased, while the infiltration of HSPD1^+^ HSP90AB1^+^ NK-HSP and HSPD1^+^ HSPH1^+^ NK-HSP decreased (Fig. 1M, N).

Because *B. parabrevis* modulated the antitumor function of NK cells, we obtained detailed characteristics of the NK cell phenotype by one-dimensional soli-expression by nonlinear stochastic embedding (One-SENSE) analysis [12]. Here, the X-axis represented the terminal phenotypic profile and the Y-axis represented the functional phenotypic profile (Fig. 1O). Based on the expression of NKG7 (an adaptive marker) and GZMK (a cytotoxic marker) [11], NK cells were divided into double-positive or double-negative cells (Fig. 1O). The profiles emphasized that most of the cells in the control group were classified as double-negative cells and the proportion of double-positive cells increased after *B. parabrevis* transplantation (Fig. 1O), which indicated that *B. parabrevis* endowed NK cells with a strong ability to adapt to environmental changes, inducing them to differentiate into memory cells that are longer-lived and more functional (adaptive) and secrete more effector cytokines (cytotoxic).

### *B. parabrevis* inhibited NK cell ferroptosis and improved antitumor immunity

**Fig. 2:**
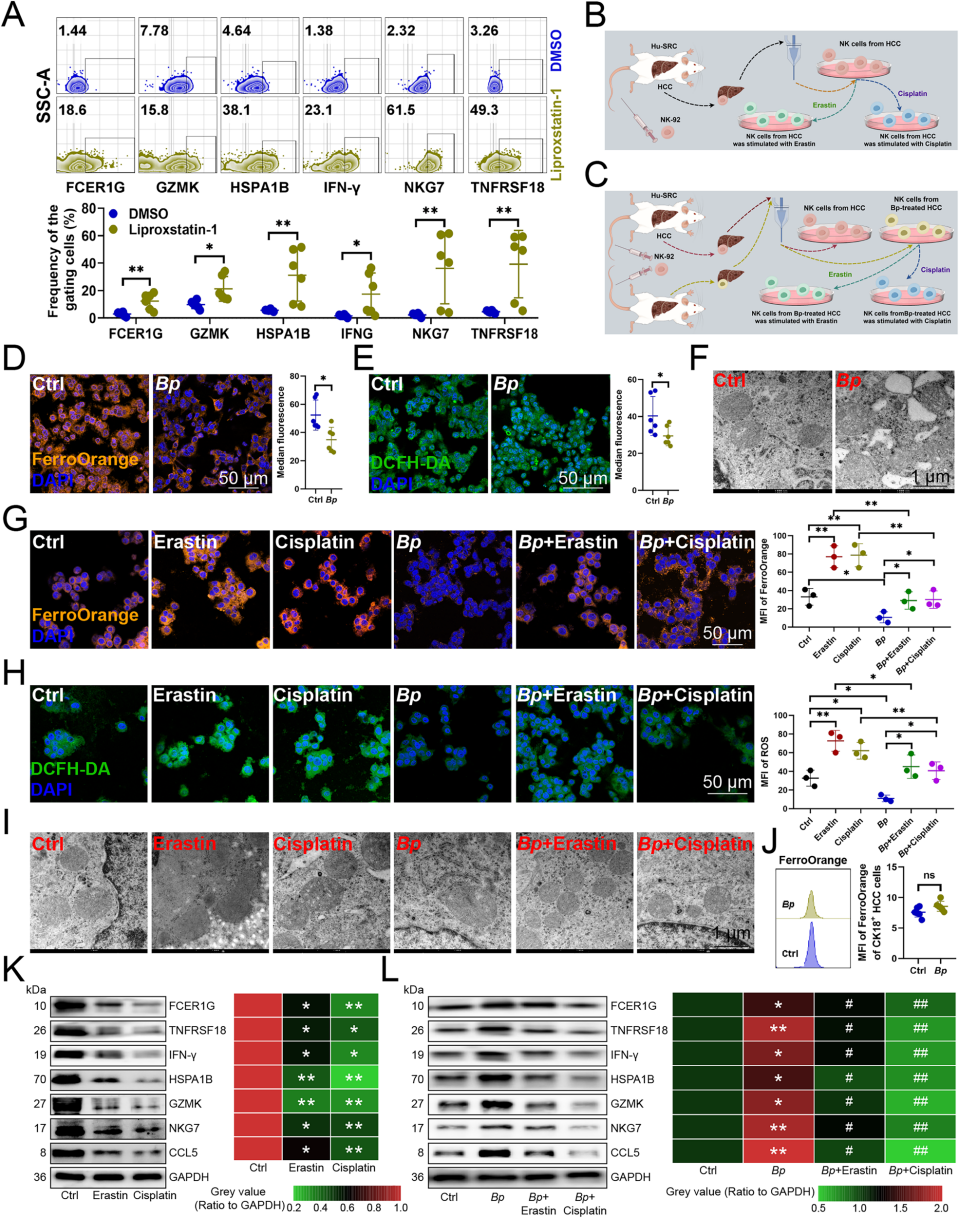
*B. parabrevis* inhibited NK cell ferroptosis and improved antitumor immunity. **A** Flow cytometry demonstrating the effect of ferroptosis inhibitor Liproxstatin-1 on the function of NK cells infiltrating HCC (*n* = 6). **B** Experimental flowchart to evaluate the effects of ferroptosis on NK cell function. **C** Experimental flowchart to evaluate the effects of *B. parabrevis* transplantation and two ferroptosis agonists on NK cell function. **D** FerroOrange probe analyzing of the effects of *B. parabrevis* transplantation on ferrous ion content in NK cells (*n* = 6). **E** DCFH-DA probe showing the effects of *B. parabrevis* transplantation on ROS content in NK cells (*n* = 6). **F** Transmission electron microscopy showing the length and morphology of each mitochondria in control and *B. parabrevis*-transplanted NK cells (*n* = 6). **G** FerroOrange probe demonstrating the effects of two ferroptosis agonists and *B. parabrevis* transplantation on the ferrous ion content in NK cells (*n* = 3). **H** DCFH-DA probe illustrating the effects of two ferroptosis agonists and *B. parabrevis* transplantation on ROS content in NK cells (*n* = 3). **I** Transmission electron microscopy showing the effects of two ferroptosis agonists and *B. parabrevis* transplantation on the length and morphology of each mitochondria in NK cells (*n* = 3). **J** FerroOrange probe analyzing the effects of *B. parabrevis* transplantation on ferrous ion content in HCC cells (*n* = 6). **K** Immunoblotting showing the effects of two ferroptosis agonists on the expression of effector genes in NK cells (*n* = 3). **L** Immunoblotting demonstrating the effects of two ferroptosis agonists and *B. parabrevis* transplantation on the expression of effector genes of NK cells (*n* = 3). ^*^indicating the statistical difference between *B. parabrevis* transplantation group and control group, ^#^indicating the statistical difference between *B. parabrevis* transplantation combined with two ferroptosis agonists treatment group and *B. parabrevis* transplantation group. **A**, **D**, **E**, **G**, **H**, **J**–**L** represented mean ± SD analyzed by unpaired *t* test. *P < 0.05, **P < 0.01.

Because the differences in bacterial composition between HCC and their paired tissues were closely related to the iron complex transport system permease protein (Fig. S2D), we analyzed whether *B. parabrevis* affected the function of NK cells by regulating their ferroptosis. Initially, we assessed the impact of Liproxstatin-1, an inhibitor of ferroptosis, on the functionality of tumor-infiltrating NK cells [11]. Our findings indicated that Liproxstatin-1 enhanced the expression of cytotoxic markers such as FCER1G, GZMK, IFN-γ, and TNFRSF18, as well as the heat shock protein HSPA1B and the adaptive marker NKG7 in NK cells (Fig. 2A). We infused human NK-92 cells into Hu-SRC mice, sorted NK cells from orthotopic HCC tissues, and then treated the cells with two ferroptosis agonists to determine the role of ferroptosis in antitumor immunity (Fig. 2B). We also transplanted *B. parabrevis* into mouse orthotopic HCC tissues and sorted the NK cells therein to analyze the immunomodulatory potential of *B. parabrevis* (Fig. 2C). The results indicated that *B. parabrevis* inhibited the levels of ferrous iron and ROS in NK cells, causing mitochondria to become smaller, membrane density to increase, cristae to decrease and disappear, and outer membrane to fragment (Fig. 2D–F). Moreover, the ferrous iron and ROS levels and mitochondrial hyperdensity induced by both agonists were suppressed by *B. parabrevis* (Fig. 2G–I). Considering that the ferroptosis of tumor cells also has a regulatory effect on tumor progression [2], we analyzed the role of *B. parabrevis* on the ferroptosis of HCC cells themselves and found that *B. parabrevis* did not cause changes in ferrous iron levels in HCC cells (Fig. 2J), which may be due to the higher sensitivity of immune cells to ferroptosis [4]. Moreover, we selected FCER1G, TNFRSF18, IFN-γ, HSPA1B, GZMK, NKG7, and CCL5 [11] to evaluate the antitumor function of NK cells and found that the two agonists inhibited the expression of these effector genes on NK cells (Fig. 2K). What’s more, *B. parabrevis* upregulated the levels of these genes, but both agonists weakened the expression-promoting effect of *B. parabrevis*, indicating that *B. parabrevis* enhanced antitumor function by inhibiting ferroptosis of NK cells (Fig. 2L).

### *B. parabrevis* suppressed NK cell ferroptosis dependently on upregulating NEDD4L

**Fig. 3:**
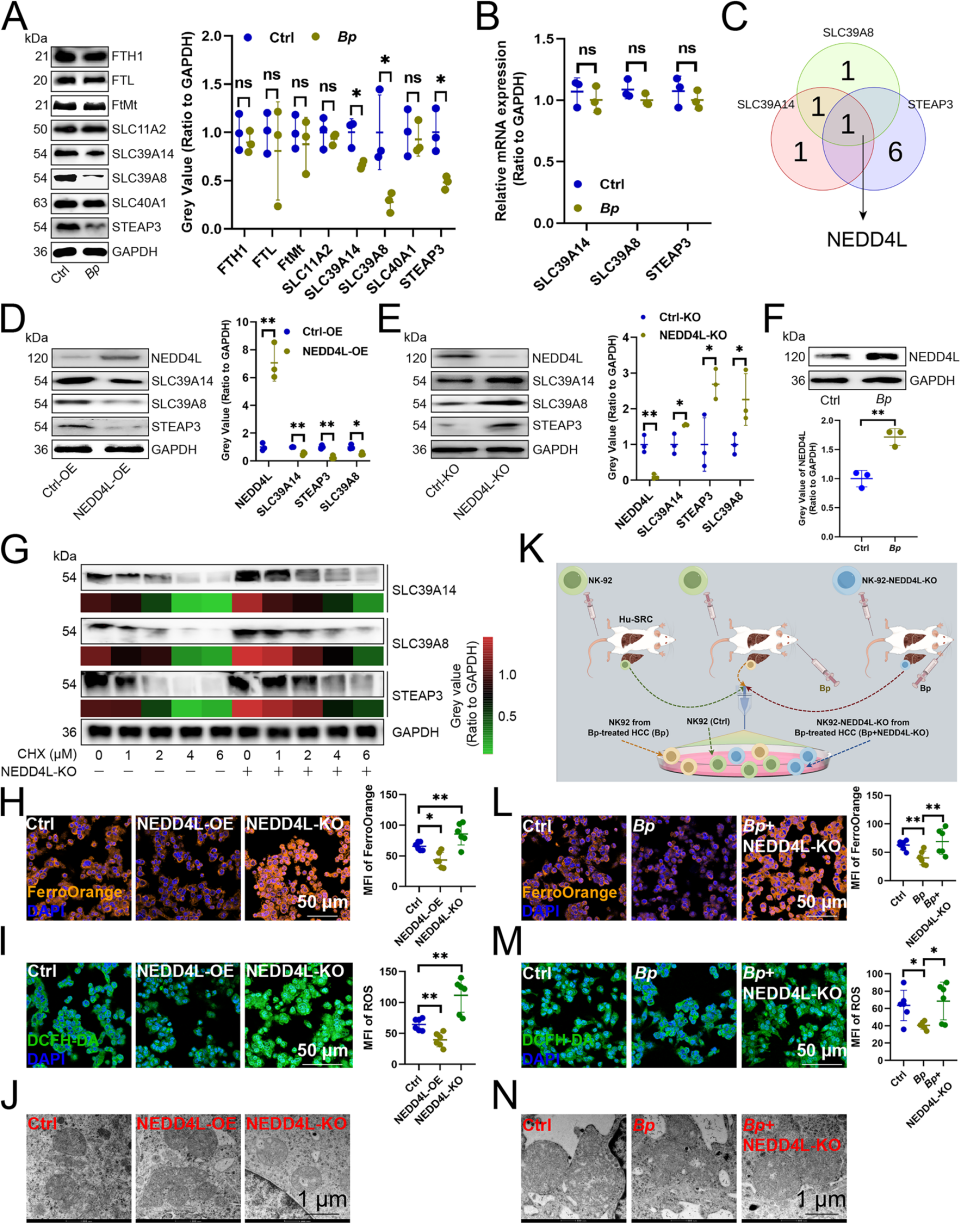
*B. parabrevis* suppressed NK cell ferroptosis dependently on upregulating NEDD4L. **A** Immunoblotting showing the expression of iron transporters in NK cells (*n* = 3). **B** RT-qPCR showing transcript levels of three iron transporters in NK cells (*n* = 3). **C** Venn diagram analysis of the common E3 ubiquitin ligases of three iron transporters. **D**, **E** Effects of overexpression (**D**) and knockout (**E**) of NEDD4L on the expression of three iron transporters in NK cells (*n* = 3). **F** Effects of *B. parabrevis* transplantation on the expression of NEDD4L in NK cells (*n* = 3). **G** Immunoblotting analysis of the expression of three iron transporters in NK cells treated with CHX (*n* = 3). **H** FerroOrange probe analyzing the effect of overexpression or knockout of NEDD4L on the ferrous ion content in NK cells (*n* = 6). **I** DCFH-DA probe demonstrating the effect of overexpression or knockout of NEDD4L on the ROS content in NK cells (*n* = 6). **J** Transmission electron microscopy showing the effects of overexpression or knockout of NEDD4L on the length and morphology of each mitochondria in NK cells (*n* = 6). **K** Experimental flowchart to evaluate the effects of *B. parabrevis* transplantation and NEDD4L knockout on NK cell function. **L** FerroOrange probe illustrating the effects of *B. parabrevis* transplantation and NEDD4L knockout on the ferrous ion content in NK cells (*n* = 6). **M** DCFH-DA probe analyzing the effects of *B. parabrevis* transplantation and NEDD4L knockout on ROS content in NK cells (*n* = 6). **N** Transmission electron microscopy showing the effects of *B. parabrevis* transplantation and NEDD4L knockout on the length and morphology of each mitochondria in NK cells (*n* = 6). **A**, **B**, **D**–**I**, **L**, **M** represented mean ±SD analyzed by unpaired *t* test. *P < 0.05, **P < 0.01. CHX Cycloheximide.

We then analyzed whether *B. parabrevis* affected ferroptosis of NK cells by affecting the expression of iron complex transport system permease protein (Fig. S1D). We found that *B. parabrevis* inhibited the protein expression of SLC39A14, SLC39A8, and STEAP3 in NK cells (Fig. 3A), but did not change their transcript levels (Fig. 3B), so we speculated that their protein inhibition may be related to ubiquitin modification. To this end, we sought which E3 ubiquitin ligase *B. parabrevis* used to degrade the three. UbiBrowser database [13] found that NEDD4L was a common high-confidence score enzyme for the three (Fig. 3C), and overexpression of NEDD4L inhibited the expression of the three (Fig. 3D), while knockout upregulated their levels (Fig. 3E). Moreover, *B. parabrevis* promoted the expression of NEDD4L (Fig. 3F). We then inhibited protein synthesis using Cycloheximide and observed that knocking out NEDD4L slowed the degradation of all three (Fig. 3G). Next, we investigated whether NEDD4L was involved in regulating ferroptosis of NK cells and found that NEDD4L overexpression inhibited ferrous ion and ROS levels in NK cells, leading to smaller mitochondria, increased membrane density, reduced and disappeared cristae, and outer membrane fragmentation, and vice versa (Fig. 3H–J). We also infused wild-type (WT) or NEDD4L-KO NK-92 cells into Hu-SRC mice, transplanted *B. parabrevis* into mouse orthotopic HCC tissues, and then sorted NK cells from HCC tissues to determine whether *B. parabrevis* inhibited NK cell ferroptosis by upregulating NEDD4L expression (Fig. 3K). We observed that *B. parabrevis* blocked the levels of ferrous iron and ROS and mitochondrial hypodensity were ameliorated by NEDD4L-KO (Fig. 3L–N). Consequently, we suggested that *B. parabrevis* impeded the ferroptosis of NK cells by enhancing the expression of NEDD4L and facilitating the degradation of SLC39A14, SLC39A8, and STAEP3.

### NEDD4L bound to SLC39A14, SLC39A8, and STEAP3

**Fig. 4:**
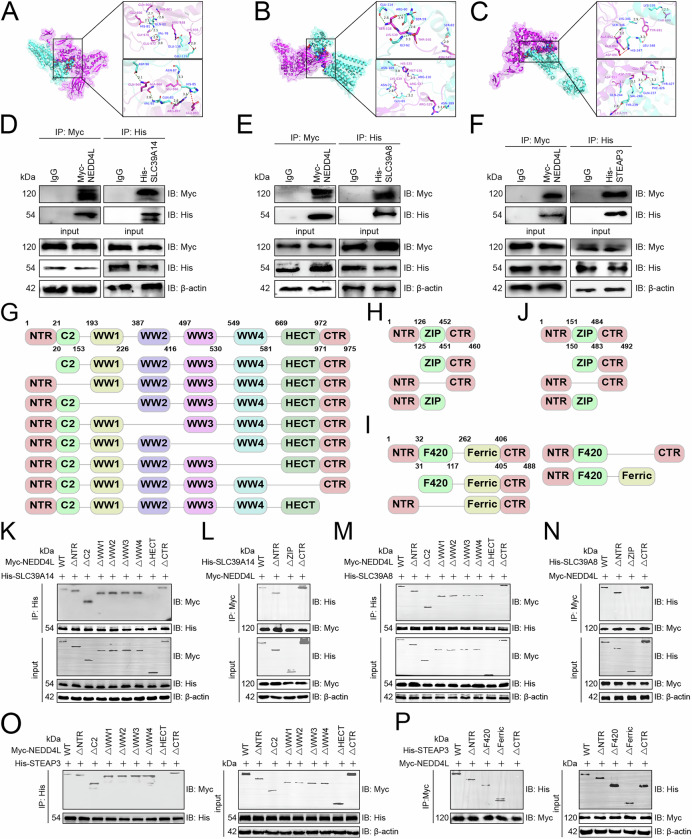
NEDD4L bound to SLC39A14, SLC39A8, and STEAP3. **A** Docking model between NEDD4L and SLC39A14 and surface map of their interface residues. Purple, NEDD4L; sky blue, SLC39A14; green dashed line, hydrogen bond; red dashed line, salt bridge. **B** Docking model between NEDD4L and SLC39A8 and surface map of their interface residues. Purple, NEDD4L; sky blue, SLC39A8; green dashed line, hydrogen bond; red dashed line, salt bridge. **C** Docking model between NEDD4L and STEAP3 and surface map of their interface residues. Purple, NEDD4L; sky blue, STEAP3; green dashed line, hydrogen bond; red dashed line, salt bridge. **D**–**F** NK-92 cell lysates were incubated with IgG control, and anti-His or -Myc tag antibodies (*n* = 3). 5% of lysate was used as input control. Cells were transfected with His-tagged SLC39A14 (**D**), SLC39A8 (**E**), and STEAP3 (**F**) constructs, respectively. Schematic representation of domain deletion mutants of NEDD4L (**G**), SLC39A14 (**H**), SLC39A8 (**I**), and STEAP3 (**J**). **K**, **L** Co-immunoprecipitation identifying the key domains that determine the binding of NEDD4L and SLC39A14 in NK-92 cells (*n* = 3). Cells were co-transfected with His-tagged SLC39A14 and Myc-tagged NEDD4L WT or deletion mutants (**K**), or Myc-tagged NEDD4L and His-tagged SLC39A14 WT or deletion mutants (**L**). **M**, **N** Co-immunoprecipitation identifying the key domains that determine the binding of NEDD4L and SLC39A8 in NK-92 cells (*n* = 3). Cells were co-transfected with His-tagged SLC39A8 and Myc-tagged NEDD4L WT or deletion mutants (**M**), or Myc-tagged NEDD4L and His-tagged SLC39A8 WT or deletion mutants (**N**). **O**, **P** Co-immunoprecipitation identifying the key domains that determine the binding of NEDD4L and STEAP3 in NK-92 cells (*n* = 3). Cells were co-transfected with His-tagged STEAP3 and Myc-tagged NEDD4L WT or deletion mutants (**O**), or Myc-tagged NEDD4L and His-tagged STEAP3 WT or deletion mutants (**P**). WT wild-type.

We further analyzed how NEDD4L bound to SLC39A14, SLC39A8, and STEAP3 in pairs. Molecular docking showed that NEDD4L was bonded to SLC39A14, SLC39A8, and STEAP3 by hydrogen bond and salt bridge (Fig. 4A–C). Co-immunoprecipitation demonstrated that NEDD4L bound to trisubstrate in pairs (Fig. 4D–F). To identify the binding regions of NEDD4L and the three iron transporters, we synthesized a series of domain deletion mutants of these four proteins (Fig. 4G–J) and found that the HECT domain of NEDD4L bound to the ZIP of SLC39A14 or SLC39A8 and the CTR of STEAP3 in pairs (Fig. 4K–P).

### NEDD4L promoted ubiquitination of SLC39A14, SLC39A8, and STEAP3

**Fig. 5:**
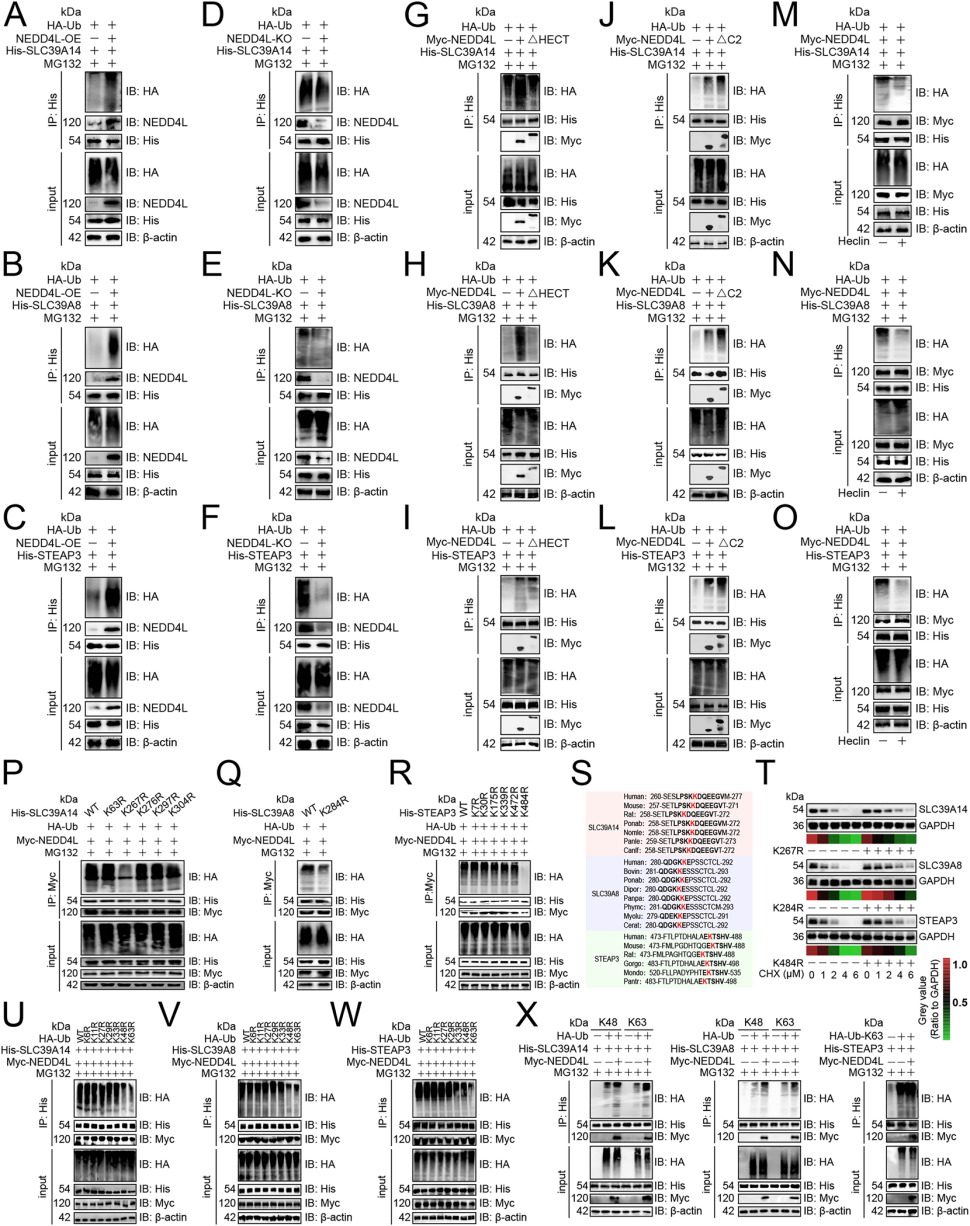
NEDD4L promoted ubiquitination of SLC39A14, SLC39A8, and STEAP3. **A**–**C** Ubiquitination of endogenous SLC39A14 (**A**), SLC39A8 (**B**), and STEAP3 (**C**) upon NEDD4L overexpression in NK-92 cells (*n* = 3). Ubiquitination of endogenous SLC39A14 (**D**), SLC39A8 (**E**), and STEAP3 (**F**) upon NEDD4L knockout in NK-92 cells (*n* = 3). NK-92 cells were co-transfected with His-tagged SLC39A14 (**G**), SLC39A8 (**H**), or STEAP3 (**I**), HA-tagged ubiquitin, and Myc-tagged WT or HECT deletion mutant of NEDD4L, and the ubiquitination of substrates was analyzed (*n* = 3). NK-92 cells were co-transfected with His-tagged SLC39A14 (**J**), SLC39A8 (**K**), or STEAP3 (**L**), HA-tagged ubiquitin, and Myc-tagged NEDD4L WT or C2 deletion mutant, and the ubiquitination of substrates was analyzed (*n* = 3). Effects of Heclin on NEDD4L-mediated ubiquitination of SLC39A14 (**M**), SLC39A8 (**N**), and STEAP3 (**O**) in NK-92 cells (*n* = 3). Ubiquitination of five His-tagged lysine mutants of SLC39A14 (**P**), one His-tagged lysine mutant of SLC39A8 (**Q**), or six His-tagged lysine mutants of STEAP3 (**R**) in NK-92 cells (*n* = 3). **S** Sequence alignment of SLC39A14, SLC39A8, and STEAP3 from different species showing the conservation of K267, K284, and K484 sites, respectively. **T** Immunoblotting analyzing the expression of three proteins in NK-92 cells transfected with His-tagged WT or lysine to arginine mutants of SLC39A14, SLC39A8, and STEAP3, and treated with CHX (*n* = 3). Determination of ubiquitinated chain linkages of SLC39A14 (**U**), SLC39A8 (**V**), and STEAP3 (**W**) in NK-92 cells. **X** Immunoblotting showing the effect of NEDD4L on K48 or K63 ubiquitin linked to SLC39A14 or SLC39A8, and K63 ubiquitin linked to STEAP3 in NK-92 cells (*n* = 3). **T** represented mean ± SD analyzed by unpaired *t* test. *P < 0.05, **P < 0.01. CHX Cycloheximide, WT wild-type.

Because NEDD4L belongs to HECT E3 ubiquitin ligase [14], we wanted to know whether NEDD4L is involved in the protein degradation of the three. We confirmed that overexpression of NEDD4L promoted the ubiquitination of the three (Fig. 5A–C), while knockout inhibited it (Fig. 5D–F). Considering that the HECT domain connected NEDD4L to the three (Fig. 4K, M, O), we analyzed the effect of the HECT domain on the ubiquitination of the substrates and pointed out that it was necessary for the ubiquitination of the three (Fig. 5G–I). The C2 domain is known to inhibit the enzymatic activity of HECT [14] and we confirmed that its deletion enhanced the ubiquitination of the three (Fig. 5J–L). Furthermore, we used Heclin, a small-molecule inhibitor of HECT, to determine whether the function of NEDD4L is pharmacologically targetable. The results showed that Heclin abolished NEDD4L-mediated ubiquitination of the three substrates (Fig. 5M–O). We also constructed lysine (K)-to-arginine (R) mutants of potential ubiquitination sites in the three substrates according to the PhosphoSitePlus database [15] and found that the K267R abolished NEDD4L-mediated ubiquitination of SLC39A14 (Fig. 5P), the K284R abolished the ubiquitination of SLC39A8 (Fig. 5Q), and the K484R abolished the ubiquitination of STEAP3 (Fig. 5R). Furthermore, residues K267 of SLC39A14, K284 of SLC39A8, and K484 of STEAP3 are evolutionarily conserved, suggesting that homologous sites in other organisms may be similarly modified (Fig. 5S). Moreover, mutation of these lysine residues inhibited NEDD4L-mediated protein degradation (Fig. 5T). Using single K-to-R mutants, we found that ubiquitin overexpressing K48R and K63R inhibited the ubiquitination of SLC39A14 or SLC39A8 (Fig. 5U, V), but only ubiquitin overexpressing K63R inhibited the ubiquitination of STEAP3 (Fig. 5W). Finally, NEDD4L enhanced SLC39A14- or SLC39A8-linked K48/K63 ubiquitination and upregulated STEAP3-linked K63 ubiquitination (Fig. 5X).

### NEDD4L inhibited NK cell ferroptosis and enhanced antitumor immunity

We infused NK-92 cells overexpressing NEDD4L and its three substrates into tumor-bearing Hu-SRC mice to evaluate whether NEDD4L-inhibited ferroptosis improved NK cell antitumor immunity. We found that NK cells overexpressing NEDD4L or *B. parabrevis* transplantation inhibited the growth of orthotopic and subcutaneous tumors in mice and improved survival time, but overexpression of three iron transporters blocked the antitumor function of NEDD4L (Fig. S3A–D). The expression of characteristic antigens of NK cells indicated that NEDD4L overexpression and *B. parabrevis* induced the expression of adaptive markers (CCL5 and NKG7), cytotoxic markers (CD7, FCER1G, GZMK, IFN-γ, and TNFRSF18), heat shock markers (HSPA8, HSPE1, and HSP90AA1), and downregulated the expression of terminal markers (B3GAT1, CDKN1A, CPEB2, HDAC9, p-PLCG2, RFX7, and SMG1) (Fig. S3E). When the three iron transporters were overexpressed, the adaptive, cytotoxic, and heat shock functions enhanced by NEDD4L overexpression were suppressed, while the terminal phenotypes suppressed by NEDD4L overexpression were enhanced (Fig. S3E).

We visualized the antigen expression of NK cells using the heatmap of unsupervised hierarchical clustering and revealed 13 NK cell subsets (Fig. S4A). We defined them as: CDKN1A^+^ CPEB2^+^ tNK, CORO1A^+^ CD7^+^ tNK, CDKN1A^+^ HSPA1B^+^ tNK, CORO1A^+^ HDAC9^+^ tNK, B3GAT1^+^ RFX7^+^ tNK, HDAC9^+^ IFN-γ^+^ tNK, CORO1A^+^ CD7^+^ tNK, RFX7^+^ CDKN1A^+^ tNK, HSPE1^+^ GZMK^+^ NK-HSP, RFX7^+^ HSPA8^+^ tNK, RFX7^+^ HDAC9^+^ tNK, RFX7^+^ B3GAT1^+^ tNK, and CDKN1A^+^ CORO1A^+^ tNK (Fig. S4B). NMDS analysis found that overexpression of NEDD4L and its substrates or *B. parabrevis* transplantation altered NK cell function (Fig. S4C). NEDD4L overexpression and *B. parabrevis* inhibited the infiltration of CORO1A^+^ HDAC9^+^ tNK, B3GAT1^+^ RFX7^+^ tNK, RFX7^+^ HSPA8^+^ tNK, RFX7^+^ HDAC9^+^ tNK, and RFX7^+^ B3GAT1^+^ tNK, and upregulated the proportion of HSPE1^+^ GZMK^+^ NK-HSP (Fig. S4D, E). When the three iron transporters were overexpressed, the infiltration of the five tNKs suppressed by NEDD4L overexpression was induced, whereas the infiltration of NK-HSPs enhanced by NEDD4L overexpression was attenuated (Fig. S4D, E).

We obtained detailed characteristics of NK cell phenotypes through One-SENSE analysis [12]. Here, the X-axis represented the terminal phenotypic profile and the Y-axis represented the functional phenotypic profile (Fig. S4F). Based on the expression of NKG7 and GZMK, NK cells were divided into double-positive and double-negative cells (Fig. S4F). The profiles highlighted that overexpression of NEDD4L or *B. parabrevis* transplantation induced the proportion of double-positive cells compared with the control group (Fig. S4F). Compared with overexpression of NEDD4L, the proportion of double-positive cells in NK cells overexpressing the three iron transporters was reduced (Fig. S4F).

### RORC acetylation promoted NEDD4L transcription

**Fig. 6:**
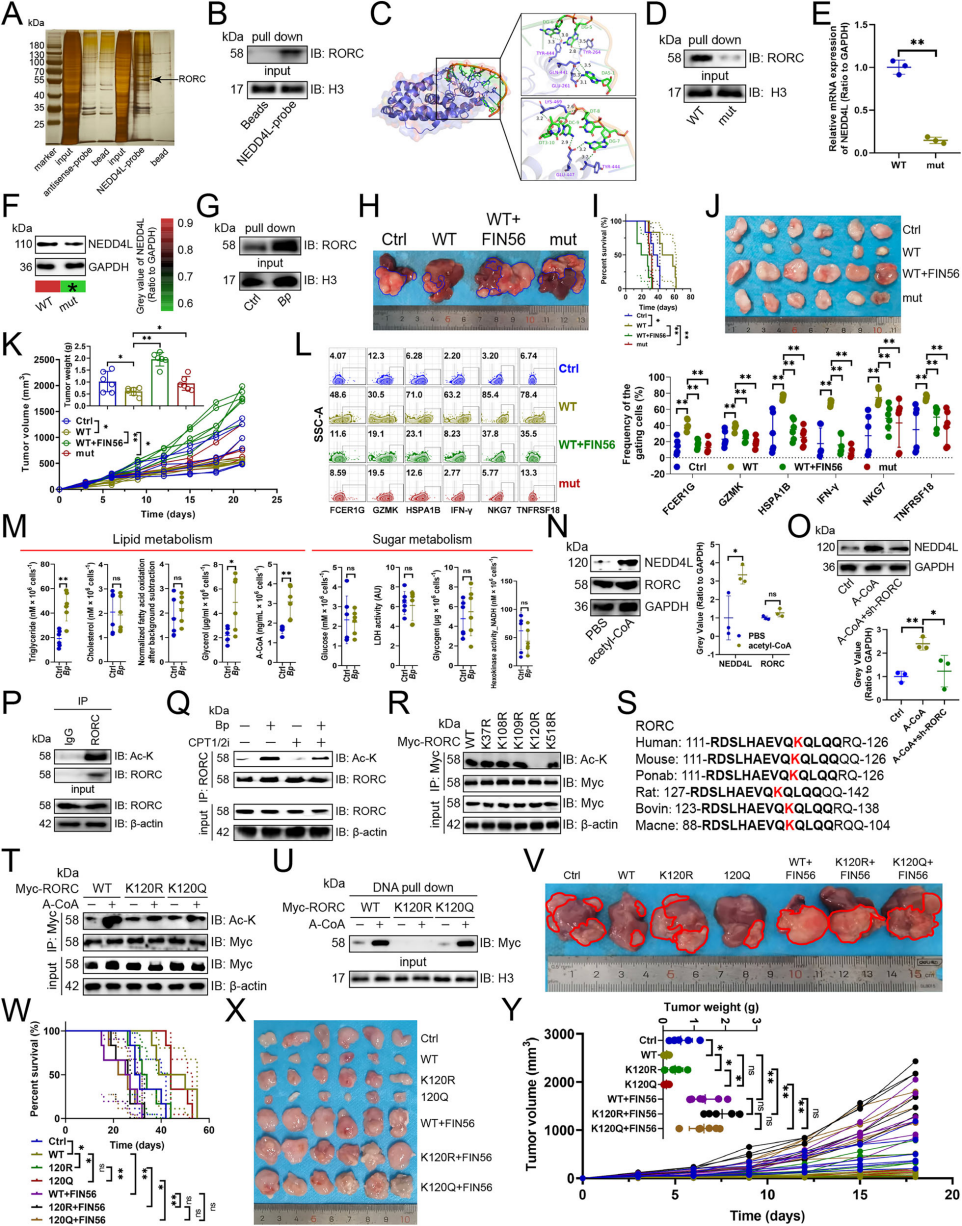
RORC acetylation promoted NEDD4L transcription. **A** DNA pull-down and mass spectrum assays showing protein on the NEDD4L promoter in NK-92 cells. **B** DNA pull-down assay confirming that RORC bound to the NEDD4L promoter in NK-92 cells (*n* = 3). **C** Docking model between RORC and NEDD4L promoter and surface map of their interface residues. Purple, RORC; green, DNA; rod-shaped structures, interacting amino acid residues; dark green dashed lines, hydrogen bonds. **D** DNA pull-down and immunoblotting assays analyzing the binding of RORC to the WT or mutant sequences of NEDD4L promoter in NK-92 cells (*n* = 3). RT-qPCR (**E**) and immunoblotting (**F**) showing the transcription and protein expression of NEDD4L, respectively (*n* = 3). NK-92 cells were co-transfected with the WT or mutant sequences of NEDD4L promoter. **G** DNA pull-down assay analyzing the effect of *B. parabrevis* transplantation on the binding of RORC to the NEDD4L promoter in NK cells (*n* = 3). **H, I** Impact of overexpressing the WT NEDD4L promoter sequence in NK-92 cells, pre-stimulation of NK-92 cells transfected with the WT promoter sequences using the ferroptosis inducer FIN56, or transfection with NEDD4L promoter sequences containing an AAAGGGGTCT deletion, on orthotopic HCC growth in Hu-SRC mice (*n* = 6). NK-92 cells were infused into mice with orthotopic HCC. **H** Representative. **I** Survival curve. **J**, **K** Effect of overexpressing the WT NEDD4L promoter sequence in NK-92 cells, pre-stimulation of NK-92 cells transfected with the WT promoter sequences using FIN56, or transfection with NEDD4L promoter sequences containing an AAAGGGGTCT deletion, on subcutaneous tumor growth in Hu-SRC mice (*n* = 6). **J** Representative. **K** Tumor mass weight and growth curve. **L** Flow cytometry evaluating the impact of overexpressing the WT NEDD4L promoter sequence in NK-92 cells, pre-stimulation of NK-92 cells transfected with the WT promoter sequences using FIN56, or transfection with NEDD4L promoter sequences containing an AAAGGGGTCT deletion, on the expression of effector genes in tumor-infiltrating NK cells in Hu-SRC mice (*n* = 6). **M** Effects of *B. parabrevis* transplantation on lipid or glucose metabolism of NK cells (*n* = 6). Immunoblotting showing the effects of acetyl-CoA (**N**) or knockdown of RORC (**O**) on NEDD4L protein expression in NK-92 cells (*n* = 3). **P** NK-92 cell lysate was incubated with anti-IgG control or -RORC antibodies (*n* = 3). 5% of lysate was used as input control. **Q** RORC acetylation was detected after *B. parabrevis* transplantation or stimulation with a CPT1/2 inhibitor (Perhexiline maleate) in NK cells (*n* = 3). **R** Evaluation of acetylation of five Myc-tagged RORC lysine mutants in NK-92 cells (*n* = 3). **S** Sequence alignment of RORC from different species showing the conservation of the K120 residue. **T** Immunoblotting showing the effect of acetyl-CoA on RORC acetylation in NK-92 cells (*n* = 3). **U** DNA pull-down assay demonstrating the binding of RORC lysine mutants to the NEDD4L promoter in NK-92 cells (*n* = 3). **V**, **W** Investigation of the impact of WT, K120R, K120Q of RORC, and FIN56-pretreatment in NK cells on orthotopic HCC growth in Hu-SRC mice (*n* = 6). (V) Representative. (W) Survival curve. **X**, **Y** Analysis of the influence of WT, K120R, K120Q of RORC, and FIN56-pretreatment in NK cells on the growth of subcutaneous tumors in Hu-SRC mice (*n* = 6). **X** Representative. **Y** Tumor mass weight and growth curve. **E**, **F**, **K**-**O**, **Y** represented mean ± SD analyzed by unpaired *t* test, **I**, **W** were analyzed by Log-rank test. *P < 0.05, ** *P* < 0.01. WT wild-type.

To determine how *B. parabrevis* promoted NEDD4L expression, we used DNA pull-down and mass spectrum assays to search for transcription factors on the NEDD4L promoter (Fig. 6A), and found that RORC bound to the promoter of NEDD4L (Fig. 6B). JASPAR database [16] predicted RORC binding to the AAAGGGGTCT sequence of the NEDD4L promoter, and molecular docking model predicted that TYR444, TYR264, GLN441, GLU264, LYS469, GLU447, and TYR444 of RORC formed hydrogen bond interactions with G6, G5, A1, G7, T8, C9, and T10 of the DNA chain (AAAGGGGTCT) (Fig. 6C). When AAAGGGGTCT was deleted, the binding of RORC to the NEDD4L promoter was inhibited (Fig. 6D), and the transcript and protein expression of NEDD4L were down-regulated (Fig. 6E, F). Moreover, *B. parabrevis* enhanced the binding of RORC to the NEDD4L promoter (Fig. 6G). In vivo experiments demonstrated that NK-92 cells transfected with the WT promoter sequence of NEDD4L not only inhibited the growth of both orthotopic and subcutaneous tumors in mice and extended survival time (Fig. 6H–K), but also increased the expression of NK cell effector genes (Fig. 6L). Conversely, pre-stimulation of NK-92 cells transfected with the WT promoter sequences of NEDD4L using the ferroptosis inducer FIN56, or transfection with NEDD4L promoter sequences containing AAAGGGGTCT deletion, diminished the anti-cancer efficacy of the WT promoter sequences (Fig. 6H–L). It has been reported that intratumoral bacteria reshape TME homeostasis in a metabolic reprogramming manner [17], so we examined the impact of *B. parabrevis* on lipid or glucose metabolism in tumor tissues. We found that *B. parabrevis* upregulated the contents of triglycerides, glycerol, and acetyl-CoA (Fig. 6M). Furthermore, acetyl-CoA upregulated the expression of NEDD4L but had no effect on RORC (Fig. 6N). Knockdown of RORC inhibited the promoting effect of acetyl-CoA on NEDD4L expression (Fig. 6O). Therefore, we speculated that acetyl-CoA enhanced the binding of RORC to the NEDD4L promoter, thereby upregulating the latter’s transcription. Because acetyl-CoA is a provider of acetyl groups and is closely related to protein acetylation [18], we hypothesized that RORC acetylation is the key to *B. parabrevis* promoting NEDD4L transcription. Our findings demonstrated that RORC underwent acetylation (Fig. 6P), a process facilitated by *B. parabrevis* transplantation (Fig. 6Q). Conversely, the administration of Perhexiline maleate, an inhibitor of CPT1/2 responsible for the oxidation of hydroxyacyl-CoA to acetyl-CoA [19], mitigated the acetylation effect induced by *B. parabrevis* (Fig. 6Q). Next, we constructed K-to-R mutants of potential acetylation sites in RORC based on the PhosphoSitePlus [15], dbPTM [20], and GPS-PAIL [21] databases, and found that only the K120R mutant abolished RORC acetylation (Fig. 6R). Since the K120 residue is evolutionarily conserved (Fig. 6S), this result suggested that acetylation may exist at this site in other organisms. It was demonstrated that K120R inhibited RORC’ s acetylation, while K-to-glutamine (Q) modification was still elevated (Fig. 6T). Furthermore, K120R abolished the effect of acetyl-CoA on the binding of RORC to the NEDD4L promoter, while K120Q maintained the effect of acetyl-CoA (Fig. 6U). In vivo rescue experiments revealed that NK cells transfected with WT RORC construct inhibited the growth of orthotopic and subcutaneous tumors in mice, thereby extending survival time (Fig. 6V–Y), whereas the K120R reduced this negative effect (Fig. 6V–Y). The K120Q, which mimicked the acetylation of RORC (Fig. 6T), exhibited antitumor effects (Fig. 6V–Y). However, when NK-92 cells were pre-treated with FIN56, the antitumor effect of WT RORC was significantly weakened, and neither mutant was able to restore the antitumor effect (Fig. 6V–Y). This suggested that RORC in NK cells relied on the inhibition of ferroptosis to inhibit tumor growth.

### RORC was acetylated and deacetylated by p300 and HDAC1 or SIRT3

**Fig. 7:**
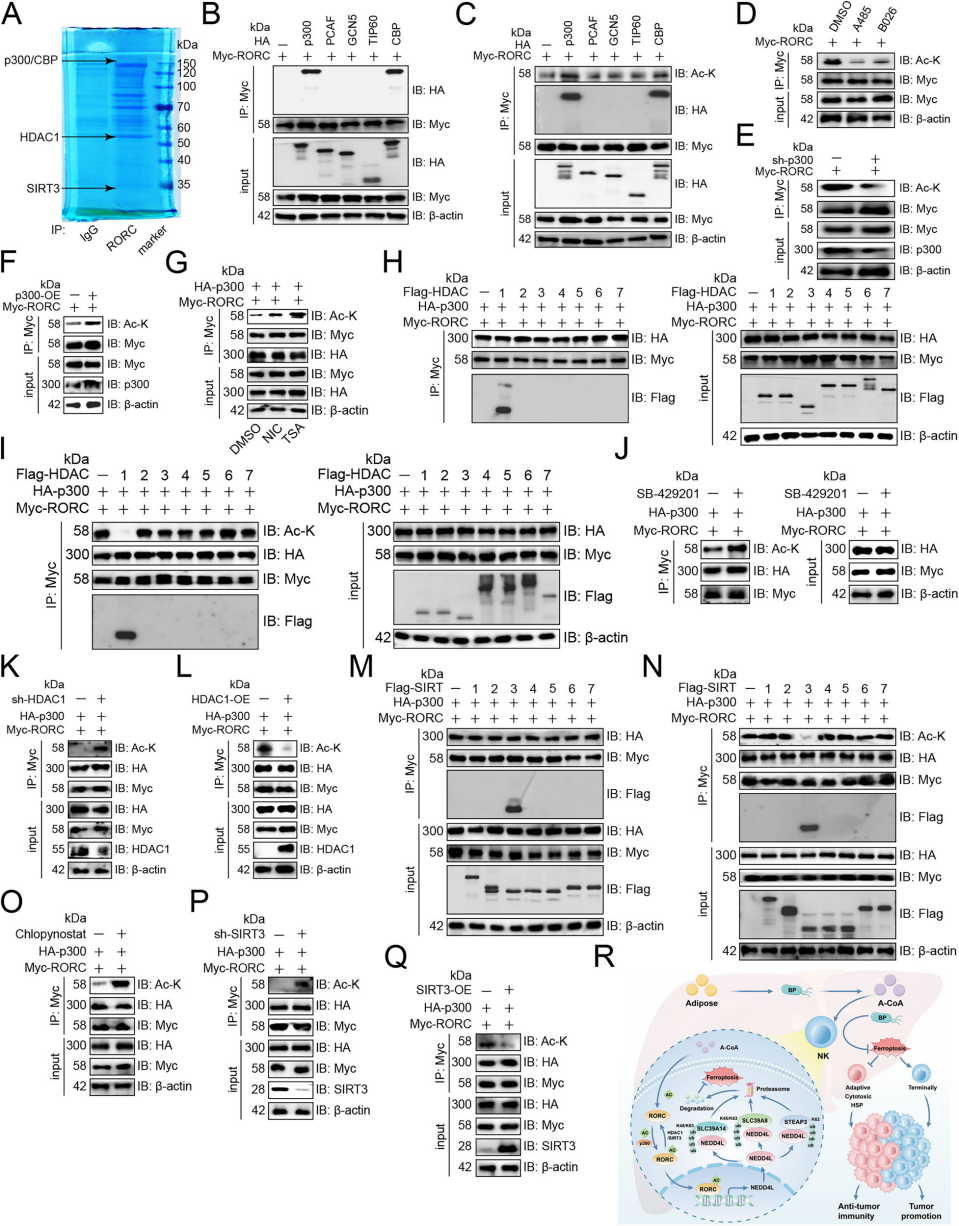
RORC was acetylated and deacetylated by p300 and HDAC1 or SIRT3. **A** Mass spectrometry showing proteins bound to RORC in NK-92 cells. **B** Immunoblotting analysis of input and protein-antibody complexes pulled down with anti-Myc antibody from NK-92 cells transfected with Myc-tagged RORC and HA-tagged acetyltransferases (*n* = 3). **C** RORC acetylation was detected upon overexpression of acetyltransferases in NK-92 cells (*n* = 3). **D** Immunoblotting showing the effects of p300 inhibitors A485 and B026 on RORC acetylation in NK-92 cells (*n* = 3). Immunoblotting showing the effect of knockdown (**E**) or overexpression (**F**) of p300 on RORC acetylation in NK-92 cells (*n* = 3). **G** Immunoblotting analysis of input and protein-antibody complexes pulled down by anti-Myc antibody from NK-92 cells transfected with Myc-tagged RORC and HA-tagged p300 in the presence or absence of the HDAC inhibitor TSA or the SIRT inhibitor NIC (*n* = 3). **H** Immunoblotting analysis of input and protein-antibody complexes pulled down by anti-Myc antibody from NK-92 cells transfected with Myc-tagged RORC, HA-tagged p300, and Flag-tagged HDACs (*n* = 3). **I** RORC acetylation was detected upon HDAC overexpression in NK-92 cells (*n* = 3). **J** Immunoblotting showing the effect of HDAC1 inhibitor SB-429201 on RORC acetylation in NK-92 cells (*n* = 3). Immunoblotting showing the effect of knockdown (**K**) or overexpression (**L**) of HDAC1 on RORC acetylation in NK-92 cells (*n* = 3). **M** Immunoblotting analysis of input and protein-antibody complexes pulled down by anti-Myc antibody from NK-92 cells transfected with Myc-tagged RORC, HA-tagged p300 and Flag-tagged SIRTs (*n* = 3). **N** RORC acetylation upon SIRT overexpression in NK-92 cells (*n* = 3). **O** Immunoblotting showing the effect of SIRT3 inhibitor Chlopynostat on RORC acetylation in NK-92 cells (*n* = 3). Immunoblotting showing the effect of knockdown (**P**) or overexpression (**Q**) of SIRT3 on RORC acetylation in NK-92 cells (*n* = 3). **R** Diagram of the mechanism by which *B. parabrevis* improved NK cell antitumor immunity.

Mass spectrometry analysis revealed that RORC was bound to acetyl transferases p300 and CBP, and deacetylases HDAC1 and SIRT3 (Fig. 7A). Our experiments also confirmed that p300 and CBP bound to RORC (Fig. 7B), but only p300 promoted RORC acetylation (Fig. 7C). p300 inhibitors A485 and B026 reduced RORC acetylation (Fig. 7D). Knockdown of p300 downregulated RORC acetylation (Fig. 7E), while overexpression decreased RORC acetylation (Fig. 7F). Because acetyltransferases and deacetylases are the yin and yang forces that control acetylation [22], we next determined the relationship between HDACs or SIRTs and RORC. NK-92 cells were treated with deacetylase inhibitors and the results showed that both the HDAC inhibitor Trichostatin A and the SIRT inhibitor Nicotinamide increased RORC acetylation (Fig. 7G). We found that only HDAC1 exhibited physical interaction with RORC and increased RORC acetylation, while other HDACs did not (Fig. 7H, I). Moreover, the HDAC1 inhibitor SB-429201 enhanced RORC acetylation (Fig. 7J). Knockdown of HDAC1 increased RORC acetylation (Fig. 7K), while overexpression did the opposite (Fig. 7L). On the other hand, only SIRT3 bound to RORC and increased RORC acetylation, while other SIRTs did not (Fig. 7M, N). Moreover, the SIRT3 inhibitor Chlopynostat enhanced RORC acetylation (Fig. 7O). Finally, knockdown of SIRT3 increased RORC acetylation (Fig. 7P), while overexpression did the opposite (Fig. 7Q).

## Discussion

NEDD4L selectively regulated ferroptosis through different ubiquitination patterns. NEDD4L promoted the ubiquitination of GPX4 [23] and STAT3 [24] to induce ferroptosis, but its mediated ubiquitination of ACSL4 [25] and LTF [26] alleviated ferroptosis. NEDD4L plays a dual role in ferroptosis by binding to different substrates in different diseases. This study revealed another ubiquitination mode of NEDD4L, namely, it catalyzed the ubiquitination of SLC39A14, SLC39A8, and STEAP3, inhibited ferroptosis of NK cells, therefore promoting antitumor immunity.

Previous reports have indicated that NEDD4L induced the degradation of TRAF3, leading to the production of proinflammatory cytokines in macrophages [27]. NEDD4L has been reported to bind to IKKα/β and IκBα, aggravating the inflammatory damage of diabetic nephropathy [28]. Our study enriched another way in which NEDD4L positively regulated inflammation—inhibiting ferroptosis of NK cells. We emphasized that *B. parabrevis* promoted NEDD4L expression and inhibited ferroptosis of NK cells, therefore leaving the TME in a highly inflammatory state.

Although NK cell ferroptosis has not been studied, recent evidence suggests that cytotoxic T cells accumulate lipid ROS and undergo ferroptosis, leading to decreased effector function [29]. Interestingly, we found that acetyl-CoA promoted RORC acetylation and increased NEDD4L transcription, therefore inhibiting NK cell ferroptosis. This suggested that acetyl-CoA had the effect of inhibiting ferroptosis. It has been reported that GPX4 blocks lipid peroxidation, restores the production of T cell effector cytokines, and promotes antitumor immune responses [30]. This study enriched the relationship between NK cell function and ferroptosis, pointing out that ferroptosis inhibited the transformation of NK cells into an adaptive subset with a strong ability to adapt to environmental changes and a long life cycle, inhibited the secretion of effector cytokines, and promoted their exit from the heat shock state [31]. However, why *B. parabrevis* did not affect the ferroptosis of tumor cells requires further research and exploration.

Acetyl-CoA is reported to generate malonyl-CoA under the catalysis of acetyl-CoA carboxylase, which is a key step in the synthesis of polyunsaturated fatty acids and a necessary step in initiating ferroptosis [32]. However, acetyl-CoA promotes cell proliferation [33], and its downstream product coenzyme Q or squalene plays an important role in preventing ferroptosis [34]. Our research indicated that acetyl-CoA has yin and yang effects on ferroptosis, especially the yin effect, which was a new discovery. Our study indicated that acetyl-CoA inhibited ferroptosis by catalyzing the acetylation of RORC and promoting its binding to the NEDD4L promoter. This may be one of the mechanisms by which acetyl-CoA promotes cell growth, and it also provided new ideas for analyzing the relationship between lipid peroxidation and ferroptosis.

In conclusion, we demonstrated that intratumoral *B. parabrevis* inhibited NK cell ferroptosis and changed the TME from “cold” to “hot”, therefore inhibiting tumor growth (Fig. 7R).

## Supplementary information

Supplementary Information

Original western blots

## Data Availability

All data relevant to the study are included in the article or uploaded as supplemental information. The raw data that support the findings of this study are deposited PRJNA1126193 (2bRAD-M, https://www.ncbi.nlm.nih.gov/bioproject/PRJNA1126193); PRJCA029040 (Mass cytometry for Hu-SRC or C57BL/6J, https://ngdc.cncb.ac.cn/bioproject/browse/PRJCA029040); PRJCA029034 (Mass spectrometry for RORC binding proteins, https://ngdc.cncb.ac.cn/bioproject/browse/PRJCA029034). In addition, we didn’t generate any new code.
