# Supplementary material for: Intratumoral Brevibacillus parabrevis enhances antitumor immunity by inhibiting NK cell ferroptosis in hepatocellular carcinoma
Source: Cell Death Dis. 2025 May 21;16(1):407. doi: 10.1038/s41419-025-07733-7 (PMC12095603; doi:10.1038/s41419-025-07733-7)
Supplement: Supplementary file 1 — Supplementary Information [file 41419_2025_7733_MOESM1_ESM.docx]

***Supplementary information for***

**Intratumoral *Brevibacillus parabrevis* enhances antitumor immunity by inhibiting NK cell ferroptosis in hepatocellular carcinoma**

**Table of contents**

**Supplementary Materials and Methods**2

**Supplementary Figures**14

**Supplementary Tables**20

**Supplementary References**25

**Supplementary Materials and Methods**

**Study design and group**

In order to study the effects of *B. parabrevis* transplantation, NEDD4L and its three substrates on ferroptosis of HCC-infiltrating NK cells, we used WT C57BL/6J mice and humanized immune mice Hu-SRC to construct carcinoma in situ and analyzed the characteristics of NK cells by mass cytometry or flow cytometry. When studying the function of a gene or the region where two proteins interact, an empty vector plasmid or a vector plasmid that overexpressed the WT gene was set as a control group. When studying the effects of a drug or compound, the solvent, such as normal saline, DPBS or DMSO, was used as a control group. For in vivo animal experiments or in vitro experiments, six replicates were set for each group, and each experiment was repeated three times. This study did not set any special inclusion or exclusion criteria for animal experiments or cytological experiments. In addition, patients who previously received palliative surgery or neo-adjuvant chemo- and/or radiotherapy were excluded. During allocation, experimental conduct, result evaluation and data analysis, we ensured that the experimenters in each link were independent and did not interfere with each other. Moreover, details regarding the number of biological replicates, the statistical methods employed, and the *P* values were provided in the figure legends.

**Bacterial culture**

*B. parabrevis* (#10027, American type culture collection, VA, USA) was stored at -80℃. *B. parabrevis* was inoculated in Trypticase Soy Broth (#Z699209, Merck) medium and incubated at 37℃ for 8 days. The bacteria were collected and washed three times with DPBS, collected with sodium chloride physiological solution (#52455, Merck), filtered with a 70 mm nylon mesh (#352350, Corning, NY, USA), and stored at 4℃ until use.

**Bioinformatics analysis**

(1) Data acquisition from the BIC and TCGA databases：The bacterial community profiles of HCC and their paired tissues were obtained from the BIC database(1) (http://bic.jhlab.tw/), and the corresponding genomes were obtained from the TCGA database (https://portal.gdc.cancer.gov/repository). (2) Prediction of immune cell infiltration: The CIBERSORT(2) R package was employed to assess the infiltration levels of 22 distinct immune cell types by utilizing gene expression data.

**Cell lines**

HEK293T (C6008, Beyotime, China) and Hep-53.4 cells were cultured in DMEM (#11965092, Gibco) supplemented with 10% FBS and 1% Penicillin-Streptomycin (PS, #TMS-AB2, Merck). NK-92 cells were cultured in MEMα (#11095080, Gibco) supplemented with 0.2 mM Inositol (#1340960, Merck), 0.1 mM β-mercaptoethanol (#444203, Merck), 0.02 mM Folic Acid (#F7876, Merck), 200 IU/ml recombinant IL-2 (#GF423, Merck), 12.5% Horse Serum (#164215, Pricella, China), and 1% PS. MycAway™ Plus-Color One-Step Mycoplasma Detection Kit (#40612ES25, Yeasen, China) was used to test for mycoplasma contamination every two weeks. All cells were identified by Short Tandem Repeat to ensure that the cell lines were accurate and uncontaminated. All cells were cultured at 37℃ in an incubator with 5% CO_2_. Acetyl-CoA (20 μM, #HY-114293, MedChemExpress), A485 (50 nM, #HY-107455, MedChemExpress), B026 (50 nM, #HY-147261, MedChemExpress), Chlopynostat (10 nM, #HY-161464, MedChemExpress), Cisplatin (30 μM, #232120, Merck), Cycloheximide (CHX, #239765, Merck), Erastin (10 μM, #329600, Merck), FIN56 (20 μM, #HY-103087, MedChemExpress), Heclin (3 μM, #HY-110204, MedChemExpress), Perhexiline maleate (10 μM, #HY-B1334A, MedChemExpress), and SB-429201 (300 nM, #HY-119017, MedChemExpress) were employed to stimulate primary NK cells or NK-92 cells for 48 h.

**Cell transfection**

pDNA3.1 (#V79020, Invitrogen) or pLKO.1 (#8453, Addgene, MA, USA) constructs were transfected using Lipofectamine™ 3000 Transfection Reagent (#L3000001, Invitrogen). For gene knockout experiments, CRISPR constructs were co-transfected with packaging plasmid pMD2.G (#12259, Addgene)/psPAX (#12260, Addgene) into HEK293T cells using Lipofectamine™ CRISPRMAX Transfection Reagent (#CMAX00008, Invitrogen) and packaged into lentivirus. 48 h after transfection, the culture medium was collected and performed to infect primary NK cells or NK-92 cells. CRISPR Target Sequence for NEDD4L: 5’-GCTCCATGGCGACCGGGCTC-3’. shRNA for HDAC1: 5’-GAAGAAAGAAGTCACCGAAGA-3’, for p300: 5’-GCAATGGACAAGGGATAATGC-3’, for RORC: 5’-GGCCCTCATATTCCAACAACT-3’, and for SIRT3: 5’-GTGGGTGCTTCAAGTGTTGTT-3’.

**Construction**

The target gene was amplified from cDNA by PCR and cloned into pDNA3.1 vector. 6 × His, Flag, HA, or Myc tag was inserted into the C-terminus of the vectors. The lysine (K) to arginine (R) or glutamine (Q) mutation of the target gene was generated by PCR and verified by sequencing.

**Patients and tissue samples**

Fresh tumor tissues were collected from 11 HCC patients who underwent hepatectomy at Fujian Medical University Union Hospital (Table S1). The residual tumor tissues, identified through pathological diagnosis, were utilized in this retrospective study. Patients/participants have provided written informed consent to participate in this study.

**Cytometry by Time-Of-Flight (CyTOF)**

Labeled antibodies: Antibodies were conjugated to isotopically enriched lanthanide metals using the Maxpar X8 Antibody Labeling Kit (#NC1648790, Standard BioTools, CA, USA) according to the manufacturer’s protocol. The labeled antibodies were stored in DPBS supplemented with 1% glycerol (#1295731, Merck), 0.05% bovine serum albumin (BSA, #B14, Thermo Scientific), and 0.05% sodium azide (#S2002, Merck) at 4℃.

Cell staining: Collect single cell suspension, add 1 μl of Cisplatin (#201064, Standard BioTools) with a final concentration of 5 μM to distinguish live cells from dead cells, incubate at 37℃ for 5 min, and then add five times the volume of Cell Staining Buffer (#201068, Standard BioTools) to terminate the labeling reaction. Centrifuge at 300 × g for 5 min, discard the supernatant, and resuspend the cells in Cell Staining Buffer. To detect cytokine expression, stimulate the cells with Cell Stimulation Cocktail (plus protein transport inhibitors) (#00-4975-93, eBioscience, CA, USA) for 6 h. After stimulation, the cells were centrifuged at 300 × g for 5 min, and diluted to 1 ml with Cell Staining Buffer to terminate the stimulation. Nonspecific signals were blocked by adding 5 μl Fc Receptor Binding Inhibitor Polyclonal Antibody (#14-9161-73, eBioscience), followed by staining with pre-mixed surface antibodies (Table S2) at 4℃ for 30 min. After washing the cells twice with Cell Staining Buffer, the cells were fixed in 200 μl of Fix and Perm Buffer (#201067, Standard BioTools) containing 250 nM Intercalator-Ir (#201192A, Standard BioTools) at 4℃ overnight. The cells were washed twice with Cell Staining Buffer and stained with premixed intracellular antibodies (Table S2) at 4℃ for 30 min. The cells were washed twice with Nuclear Antigen Staining Buffer (#201063, Standard BioTools), resuspended in Cell Acquisition Solution Plus for CyTOF XT (#201244, Standard BioTools), and then mixed with 20% EQ Four Element Calibration Beads (#201078, Standard BioTools). Data were acquired using a CyTOF XT (Standard BioTools) and saved as fcs files.

Preprocessing of mass cytometry data: Raw data were normalized using the MATLAB version of the Normalizer tool(3). Cells were assigned by manually gating on Event length and DNA (^191^Ir and ^193^Ir) channels, followed by the dead cell discrimination analyzing ^195^Pt expression using a FlowJo Software (BD Biosciences, CA, USA). Doublets were excluded using Gaussian discrimination channels. Next, data were concatenated and de-barcoded using Boolean gating. The normalized data containing living cells from every individual sample were manually exported from FlowJo Software and imported into RStudio Software (RStudio) using the R packages “flowCore”(4) and “flowWorkspaceData”(5). Before automated high-dimensional data analysis, the mass cytometry data were transformed with a cofactor in the range of 5 and 60 using an inverse hyperbolic sine function(6). After live, single, compensated cells were exported and imported into RStudio Software. Before automated high-dimensional data analysis, data were transformed using an inverse hyperbolic sine function with a cofactor in the range of between 300 and 600. Additionally, all data were normalized between 0 and 1 to match the 99-999^th^ percentile of the combined samples in each batch.

Automated subset identification: To identify NK cell subsets accurately, we first performed step 1 of FlowSOM clustering on the preprocessed and combined mass cytometry dataset to generate a starting point of 100 nodes(7). The respective k-value was manually chosen (in the range of between 20 and 30); identified subsets were annotated and merged based on a similarity of antigen expression to uphold the biological relevance of the dataset. Manually-annotated subsets were used to calculate the relative frequencies of NK cell subsets. Heatmaps showed the median expression levels of all markers for each merged subset and were plotted using the R package “pheatmap”. From mass cytometry datasets, we pre-selected major subsets and performed additional FlowSOM(7) analysis to identify smaller cell subsets. We calculated the median antigen expression among selected cell types of the mass cytometry batch using the R package “dplyr”. For data visualization, we applied dimensionality reduction techniques. For a complex overview of the immune compartment, we used t-SNE(8). To create a t-SNE of isolated NK cells, we pooled equally proportioned 120,000 NK cells from the datasets from the CyTOF batch. Categorical One-Dimensional Soli-Expression by Nonlinear Stochastic Embedding (One-SENSE) analysis(9) generated one-dimensional t-SNE of equally pooled NK cells of the CyTOF batch, where axis was calculated using terminal or functional markers. The one-dimensional t-SNEs were aligned with two heatmaps, displaying terminal or functional cell profiles using the R package “gplots”.

**DNA pull-down assay**

500 μg of nuclear protein extract, 5 μg of biotin-labeled NEDD4L promoter double-stranded oligonucleotide probe, and 100 μl of streptavidin magnetic beads (#Bes5004, Bersinbio, China) were incubated overnight at 4℃. After centrifugation at 5000 × g for 5 min in a precooled fixed-angle centrifuge, the protein-DNA-probe complex was resuspended in 30 μl of loading buffer, and the mixture was boiled for 10 min. The complexes were separated using SDS-Polyacrylamide Gel Electrophoresis (SDS-PAGE, #89888, Thermo Scientific) and analyzed by silver staining or immunoblotting.

**Ferrous ion determination**

FerroOrange (#SCT210, Merck) was used to measure ferrous iron in the cytoplasm. The cells were incubated with serum-free medium containing 1 μM FerroOrange at 37℃ for 30 min and then examined using a Leica TCS SP8 microscope (Leica, Germany).

**Flow cytometry analysis**

The cells were resuspended in Flow Cytometry Staining Buffer (#00-4222-26, eBioscience) and stained with primary antibodies (Table S2) at 4℃ for 30 min, followed by staining with secondary antibodies (Table S2) at 4℃ for 30 min. The cells were washed twice in Flow Cytometry Staining Buffer and resuspended. Samples were collected and recorded in a FACSAria™ III flow cytometer (BD Biosciences), and data were analyzed using a FlowJo software.

**FISH assay**

Tissue sections were washed in 0.1 M Tris-HCl, pH 7.4 (ST774, Beyotime, China) for 15 min, and bacterial cell walls were hydrolyzed with lysozyme (10 mg/ml, 10837059001, Merck) for 30 min. The *B. parabrevis* probe was used to labelled bacterial. *B. parabrevis* colonization was detected on a Leica TCS SP8 microscope. The sequence of the probe was CCGCCGCACACGATGGGCAG, and the coupled fluorophore was FITC.

**Immunoblotting and co-immunoprecipitation**

Cells were washed twice with DPBS and fully lysed with Novex™ Tricine SDS Sample Buffer (#LC1676, Invitrogen) supplemented with 1% PMSF Protease Inhibitor (#36978, Thermo Scientific). The cell lysate was boiled for 10 min and subjected to SDS-PAGE, followed by incubation with primary antibodies (Table S2). For co-immunoprecipitation, cell lysates were incubated with tag or primary antibodies (Table S2) at 4°C for 1 h and then incubated with Protein A/G Magnetic Beads (#88802, Thermo Scientific) at 4°C for 2 h. The protein-antibody-magnetic bead complex was washed three times with Novex™ Tricine SDS Sample Buffer and then subjected to immunoblotting. For ubiquitin experiments, cells were treated with MG132 (10 μM, #474791, Merck) for 4 h before harvesting.

**Immunohistochemistry**

Frozen sections were fixed using Paraformaldehyde 4% (#I28800, Thermo Scientific) for 30 min. Subsequently, permeabilization was achieved by treating the sections with Triton™ X-100 Surfact-Amps™ Detergent Solution (#85111, Thermo Scientific) for 20 min. Following this, the sections were blocked using a 5% BSA solution for 1 h. The primary antibody (Table S2) was then applied to the sections and incubated overnight at 4 °C. After three washes with PBS Tween 20 Buffer (#28352, Pierce, CA, USA), the sections were exposed to the secondary antibody (Table S2) for 30 min at room temperature. Finally, the slides were mounted using an anti-quenching reagent and the images were captured using a Leica TCS SP8 microscope.

**Isolation of NK cells**

Initially, the dissociation of mouse or patient tumor tissue was conducted as detailed in the “Preparation of patient-derived tumor tissue cells” subsection within the “Mouse model” section. Human and mouse NK cells were purified from single cell suspension from tumor tissue using MojoSort™ Human NK Cell Isolation Kit (#480054, Biolegend) and MojoSort™ Mouse NK Cell Isolation Kit (#480049, Biolegend), respectively.

**Mass spectrometry**

Proteins bound to RORC were co-immunoprecipitated with RORC antibody (Table S2) and the purified proteins were sent to 10K Genomics, Shanghai, China for mass spectrometry analysis.

**Metabolite determination**

According to the manufacturer’s protocol, we used the Triglyceride Assay Kit-quantification (#ab65336, Abcam, MA, USA), Mouse Total cholesterol ELISA Kit (#ab285242, Abcam), Fatty Acid Oxidation Assay (#ab217602, Abcam), Glycerol Assay Kit (Cell-Based) (#ab133130, Abcam), CheKine™ Micro Acetyl Coenzyme A (acetyl-Coa) Assay Kit (#KTB1260, Abbkine, China), Glucose Assay Kit (#ab65333, Abcam), LDH Assay Kit/Lactate Dehydrogenase Assay Kit (Colorimetric) (#ab102526, Abcam), CheKine™ Micro Glycogen Assay Kit (#KTB1340, Abbkine), and CheKine™ Micro Hexokinase (HK) Activity Assay Kit (#KTB1123, Abbkine) to detect triglyceride, cholesterol, fatty acid oxidation, glycerol, acetyl-CoA, glucose, lactate dehydrogenase activity, glycogen activity, and hexokinase activity in tumor infiltrating NK cells, respectively.

**Molecular docking analysis of DNA with protein**

3dRNA(10) was used to construct the 3D structure of DNA. Five models were constructed for each structure. The energy was minimized using the minimize module of Discovery Studio software, and the structure with the lowest energy was selected as the optimal structure. The protein structure was constructed using I-TASSER(11) and subsequently visualized using PyMol, and Mgtools was performed to add/remove hydrogens, calculate charges, and merge nonpolar hydrogens. The ligands were docked to the receptors using AutoDock Vina(12), and the higher scoring conformations were selected and visualized using PyMol and LigPlus(13).

**Molecular docking analysis of protein with protein**

HDOCK(14) was used as a molecular docking program to analyze the interaction of NEDD4L with SLC39A14, SLC39A8 and STEAP3, respectively. PyMol software was used to separate the original ligand and protein structure, dehydrate to remove organic matter, and then the Prepare module of Discovery Studio software was used to prepare the protein, such as hydrogenation and protonation. LigPlus software(13) was performed to analyze the forces between two proteins in two dimensions. The protein interaction interface was analyzed using the Analysis Interface module of Discovery Studio software, and PyMol software was applied to draw the interacting amino acid residues between two proteins.

**Reactive oxygen species (ROS) detection**

Cells were stained with 10 μM 2’,7’-Dichlorofluorescein diacetate (DCFH2-DA, #D6883, Merck) in serum-free medium at 37°C for 30 min and then detected using a Leica TCS SP8 microscope.

**Real‑time Quantitative PCR (RT-qPCR)**

Total RNA was extracted and purified with FastPure Cell/Tissue Total RNA Isolation Kit V2 (#RC112-01, Vazyme, China), followed by reverse transcription using HiScript II Q Select RT SuperMix for qPCR(+gDNA wiper) (#R233-01, Vazyme). RT-qPCR was performed on Applied Biosystems 7500 and 7500 Fast Real-Time PCR Systems (Thermo Scientific) using ChamQ SYBR qPCR Master Mix (#Q311-02, Vazyme). PCR cycling conditions were 95°C for 30 s, followed by 40 cycles of 95°C for 10 s, 63°C for 10 s, and 72°C for 30 s. The cDNA melting curve was set as usual. Relative gene expression was calculated using 2^−ΔΔCT^ method, calibrated against GAPDH. The primers used in this study were as follows: GAPDH-F, 5’-GGAGCGAGATCCCTCCAAAAT-3’, GAPDH-R, 5’-GGCTGTTGTCATACTTCTCATGG-3’; NEDD4L-F, 5’-GACATGGAGCATGGATGGGAA-3’, NEDD4L-R, 5’-GTTCGGCCTAAATTGTCCACT-3’; SLC39A8-F, 5’-ATGCTACCCAAATAACCAGCTC-3’, SLC39A8-R, 5’-ACAGGAATCCATATCCCCAAACT-3’; SLC39A14-F, 5’-GAGGCTCACGCTTCATCCC-3’, SLC39A14-R, 5’-CCCTCGCCATACCGATGTATTA-3’; STEAP3-F, 5’-CTCCCCGGAGGTCATCTTTG-3’, STEAP3-R, 5’-TCTTGCTCTGTAGGGTTGCTC-3’.

**Transmission electron microscopy**

Cells were fixed with 2.5% glutaraldehyde (#8.20603, Merck) at room temperature for 2 h and at 4°C overnight. Cells were fixed with 1% ruthenium tetroxide (#20427-56-9, Macklin, Canada) for 1 h, washed, dehydrated through graded ethanol (#1012768, Merck) (30, 50, 70, and 95%, 5 min each step), embedded, and polymerized at 60°C for 48 h. Ultrathin sections of 85 nm were cut and observed under a FEI Tecnai T12 Transmission Electron Microscope (FEI, OR, USA) at 120 keV. For analysis, the length and morphology of each mitochondria was determined in a ImageJ software (NIH, MA, USA) after manual drawing of individual organelles.

**2bRAD sequencing for Microbiome (2bRAD-M)**

Library preparation: Genomic DNA was extracted using TIANamp Micro DNA Kit (#DP316, Tiangen, China). The 2bRAD-M library was prepared according to the following protocol(15): DNA (200 ng) was heated at 37°C for 3 h with 4 IU of BcgI enzyme (#R0545, New England Biolabs, MA, USA). The DNA fragment was then ligated to the vector. 5 µl of digested DNA, 10 µl of vector, and 800 IU of T4 DNA Ligase (#M0202S, New England Biolabs) were mixed and ligated at 4°C for 12 h. The products were amplified by PCR and electrophoresed on 8% polyacrylamide gel at 400 V for 35 min. A band of approximately 100 bp was excised from the polyacrylamide gel and the DNA was diffused from the gel into DEPC-Treated Water (#AM9916, Invitrogen). PCR was performed using platform-specific barcoded primers. The PCR products were purified using the QIAquick PCR purification kit (#28104, Qiagen, MA, USA) and then sequenced using the Illumina Nova PE150 platform. 2bRAD-M was performed at OE BioTech Co., Ltd., Qingdao.

Strain identification: Scan reads according to the recognition sites of type IIB restriction endonucleases, extract sequences containing enzyme-cut fragments, and generate clean reads according to the following conditions: (1) Remove reads containing more than 8% unknown bases; (2) Remove low-quality bases (the number of bases with quality values ​​lower than Q30 exceeded 20% of the total number of bases). The quality controlled 2bRAD markers were mapped to the 2bRAD marker database. To control for false positives in species identification, a G score was assigned to each species in the sample: G score _species i_ = $\sqrt{si\times ti}$ (*s*: number of reads of all 2bRAD tags belonging to species *i* in the sample. *t*: number of all 2bRAD tags of species *i* in the sample). The G value is the harmonic mean of the read coverage of 2bRAD markers belonging to a species and the number of all 2bRAD markers in the species. The G value was set to 5(16). Next, the average read coverage of all 2bRAD markers for each species was calculated, which represented the number of individuals belonging to a species in a sample at a given sequencing depth. Finally, the relative abundance of a particular species was calculated according to the formula: $\mathrm{species}i=\frac{si/ti}{\sum_{i=1}^{n} si/ti}$。

Bioinformatics analysis: We used principal co-ordinates analysis (PCoA) for visualization of complex and multidimensional data. When the raw counts were normalized to the Operational Taxonomic Unit (OUT) table of relative abundance, taxa of the same type were summarized at the phylum, class, order, family, and genus levels. At a taxonomic level, the relative abundance of a microorganism was calculated as the number of tags corresponding to that microorganism divided by the total number of tags in the sample. Student’s *t*-test was used to identify significantly different species at different levels. Linear discriminant analysis (LDA) with Linear discriminant analysis Effect Size (LEfSe) was utilized to identify bacterial species. The LDA score indicated the effect size of each OTU, and OTUs with an LDA score > 3.0 were defined as differentially abundant OTUs. Metastats were used to detect differences in microbial abundance between groups. Finally, the differences in the microbiome within tumors were visualized using Metastats complex heatmaps.

**Supplementary Figures**


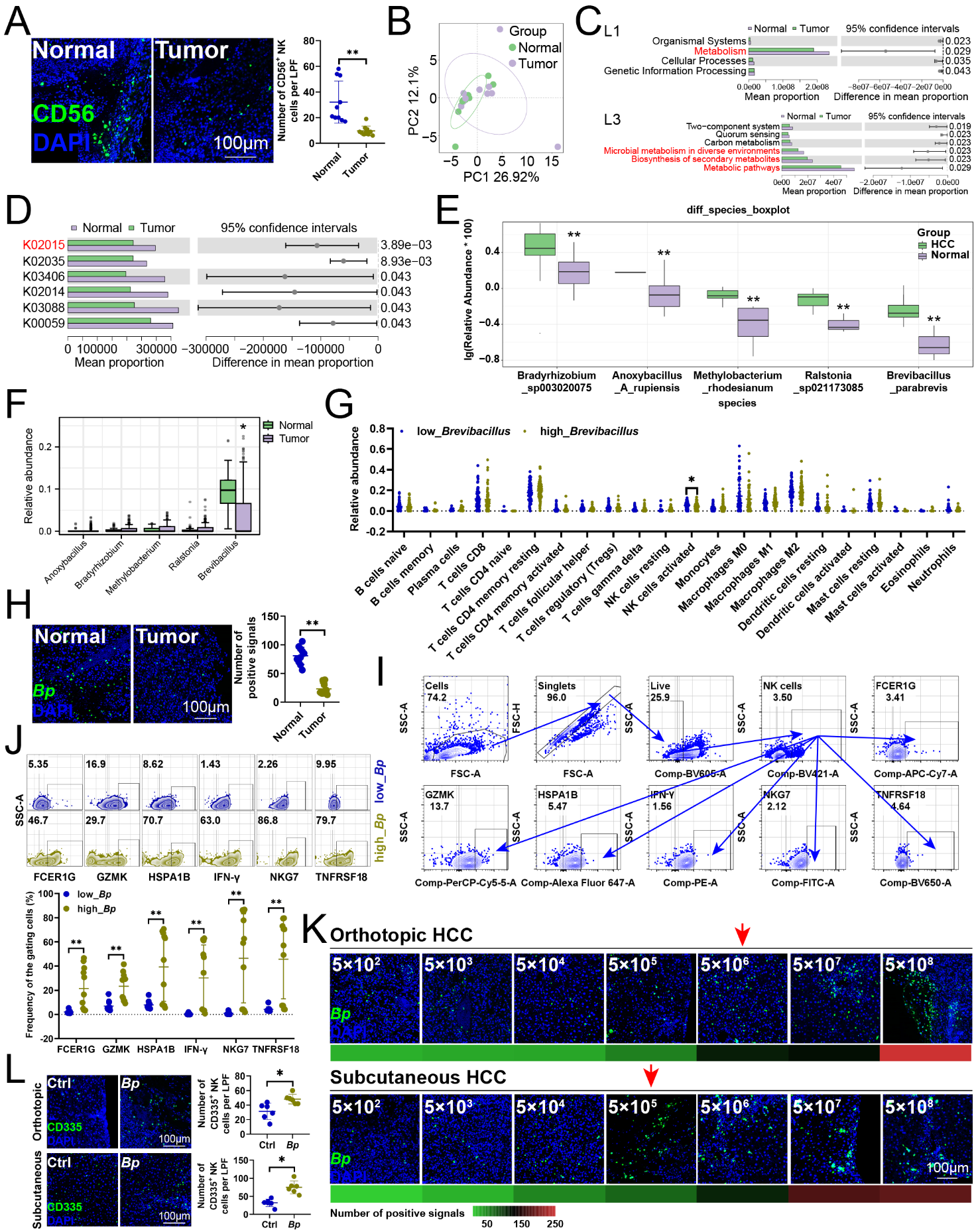


**Fig. S1 Characteristics of intratumoral bacterium in patient HCC*.*** **A** Immunofluorescence showing the infiltration of NK cells in patient cancer and paired tissues (*n* = 10). **B** NMDS analysis comparing the similarity of microbial community between cancer and paired tissues (*n* = 10). **C** Bar chart of KEGG difference analysis (*n* = 10). The left bar chart showing the mean proportion of the pathway in each group, and the right bar chart demonstrating the 95% confidence interval of the inter-group difference comparison and the corresponding significant *P* value. **D** KEGG predicting differential metabolites between cancer and paired tissues (*n* = 10). **E** Differences between cancer and paired tissues at the species levels (*n* = 10). **F** The abundance differences of *Brevibacillus*, *Anoxybacillus*, *Bradyrhizobium*, *Methylobacterium*, and *Ralstonia* between cancer and paired tissues at the genus level from the BIC database (*n* = 371). **G** CIBERSORT algorithm evaluating immune cell scores between low and high *Brevibacillus* tissues from the TCGA database (*n* = 371). **H** FISH assay showing the *B. parabrevis* levels in patient HCC and paired tissues (*n* = 10). **I, J** Flow cytometry elucidating the relationship between varying abundance of *B. parabrevis* and the expression levels of specific markers on NK cells infiltrating HCC (*n* = 10). (I) Gating logic. (J) Representational and quantitative analysis. **K** FISH assay quantifying the *B. parabrevis* levels within orthotopic or subcutaneous HCC tissues following administration via gavage with differing concentrations of *B. parabrevis* (n = 6). The red arrow denoted the transplant concentration suitable for replicating the extent of *B. parabrevis* present in patient HCC tissues. **L** Immunofluorescence demonstrating the impact of administering 5 × 10^6^ or 5 × 10^5^ CFU of *B. parabrevis* on the infiltration of NK cells within orthotopic or subcutaneous HCC (*n* = 6). (A), (E-H) and (J-L) mean ± SD analyzed by unpaired *t* test. **P* < 0.05, ***P* < 0.01. *B. parabrevis*, *Brevibacillus parabrevis*; FISH, Fluorescence in situ hybridization assay; HCC, hepatocellular carcinoma; NMDS, Non-metric MultiDimensional Scaling.


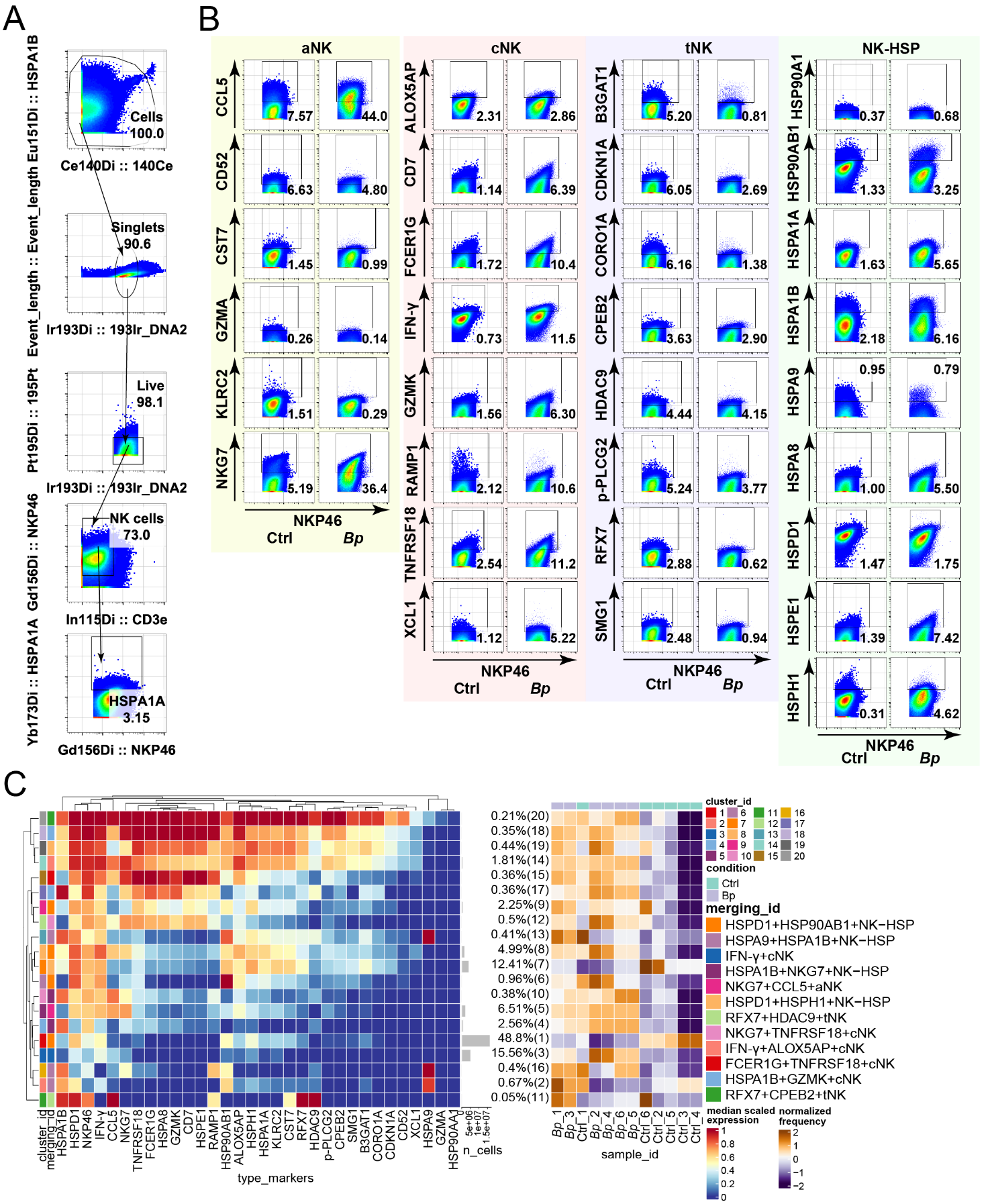


**Fig. S2** ***B. parabrevis* enhanced the antitumor activity of NK cells. A, B** Mass cytometry showing the effect of *B. parabrevis* transplantation on the expression levels of NK cell characteristic antigens (*n* = 6). (A) Gating logic. (B) Visualization of Fig. 1K. **C** Heatmap showing the median expression levels of antigens used to generate SOM (*n* = 6). (C) were analyzed by Euclidean distance.


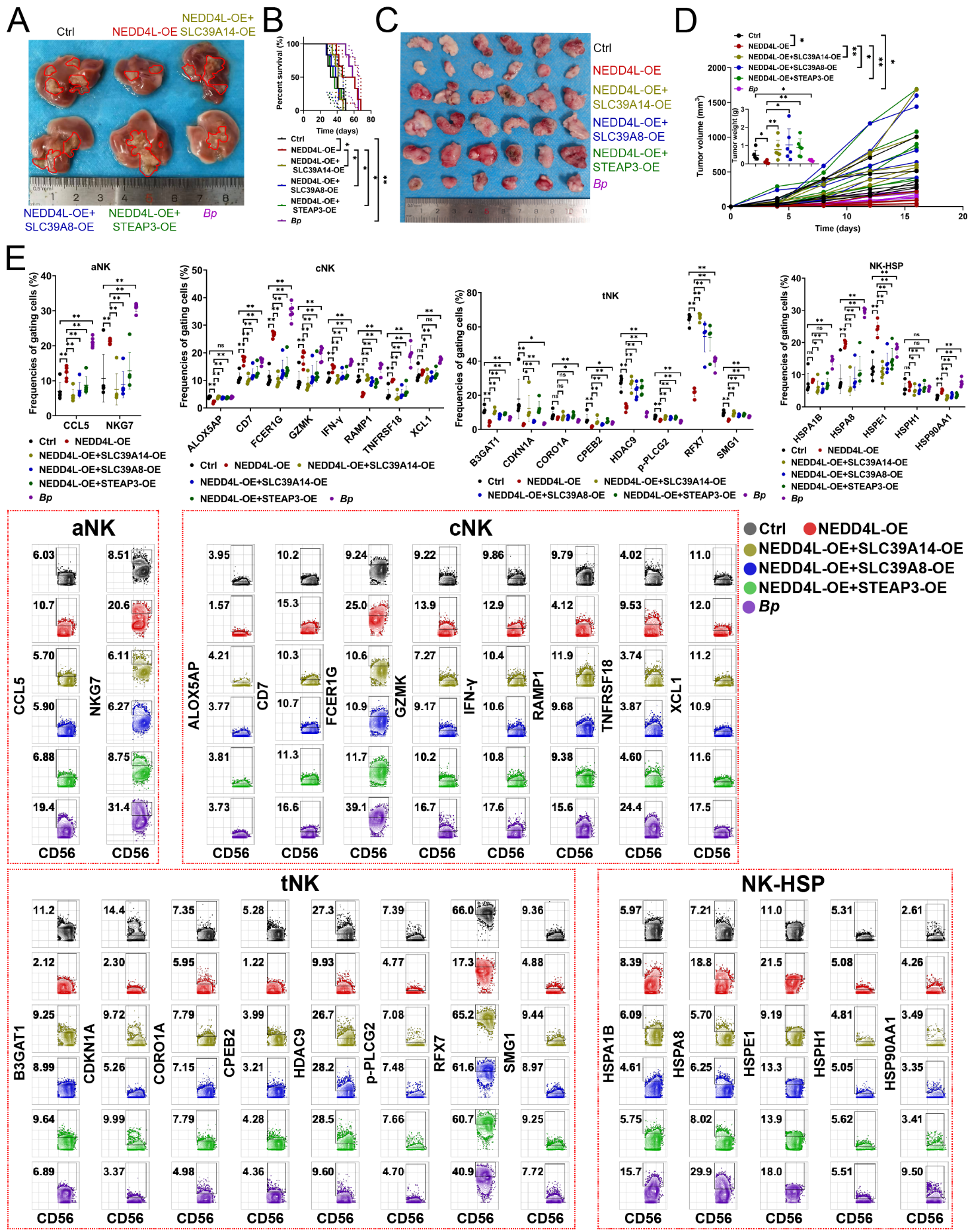


**Fig. S3 NEDD4L inhibited NK cell ferroptosis and enhanced antitumor immunity.** **A, B** Growth of orthotopic HCC in Hu-SRC mice (*n* = 6). NK-92 cells overexpressing specific genes were injected into the tail vein or *B. parabrevis* was given by gavage. (A) Representative. (B) Survival curve. **C, D** Growth of subcutaneous tumors in Hu-SRC mice (*n* = 6). NK-92 cells overexpressing specific genes were injected into the tail vein or *B. parabrevis* was injected into the tumor. (C) Representative. (D) Tumor weight and growth curve. **E** Mass cytometry analysis showing the expression levels of NK cell characteristic antigens in (A) (*n* = 6). (B) was analyzed by log-rank test, (D) and (E) represented mean ± SD analyzed by unpaired *t* test. **P* < 0.05, ***P* < 0.01.


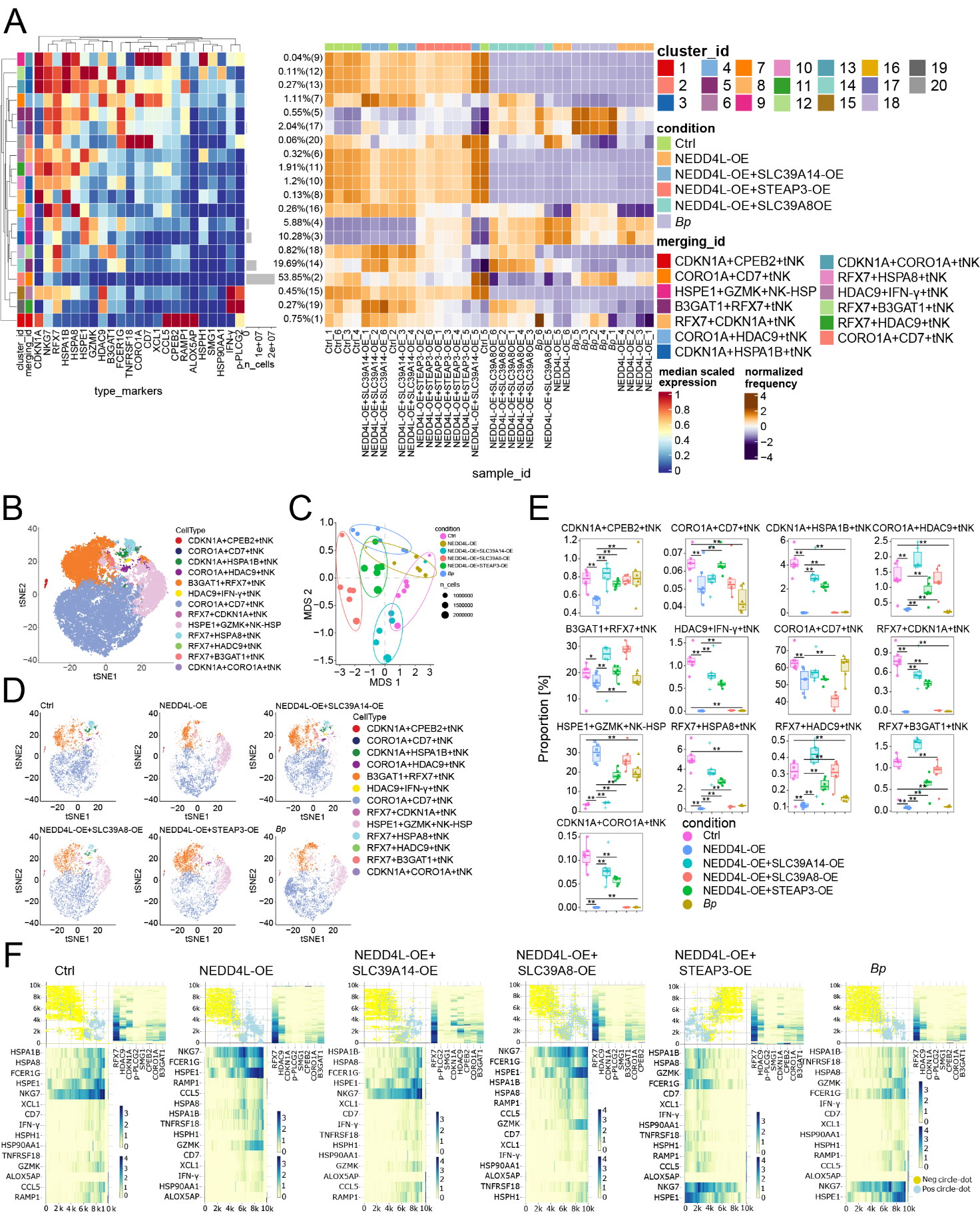


**Fig. S4 NEDD4L inhibited NK cell ferroptosis and enhanced antitumor immunity.** **A** Heatmap showing the median expression levels of antigens used to generate SOM (*n* = 6). **B** SOM overlaid on mass cytometry data of NK cells (*n* = 6). **C** NMDS analysis comparing the similarity of expression levels of NK cell characteristic antigens in different groups (*n* = 6). **D** Mosaic plot of single NK cells (*n* = 6). **E** Boxplot showing the proportion of NK cell subpopulations (*n* = 6). **F** One-SENSE analysis comparing the terminal and functional profiles of NK cells (*n* = 6). (E) represented mean ± SD analyzed by unpaired *t* test. **P* < 0.05, ***P* < 0.01. NMDS, Non-metric MultiDimensional Scaling; One-SENSE, one-dimensional soli-expression by nonlinear stochastic embedding.

**Supplementary Tables**

**Table S1.** Clinical information of patients with hepatocellular carcinoma at our center.

| **ID** | **SEX** | **AGE** | **Cirrhosis (negative/positive)** | **Grade** | **Tumor site** | **T** | **N** | **M** | **AJCC** |
| --- | --- | --- | --- | --- | --- | --- | --- | --- | --- |
| Patient_1 | Male | 77 | Positive | Ⅱ | Left paramedian sector | T3 | N1c | M0 | 3B |
| Patient_3 | Male | 43 | Positive | III | Right paramedian sector | T4 | N0 | M1 | 4B |
| Patient_4 | Female | 50 | Positive | I | Left paramedian sector | T3 | N0 | M0 | 3A |
| Patient_5 | Male | 47 | Negative | II | Left paramedian sector | T2 | N0 | M0 | 2 |
| Patient_6 | Male | 40 | Positive | II | Left paramedian sector | T3 | N0 | M0 | 3A |
| Patient_7 | Female | 46 | Positive | II | Right paramedian sector | T4 | N0 | M0 | 3B |
| Patient_8 | Male | 50 | Positive | II | Left paramedian sector | T3 | N0 | M0 | 3A |
| Patient_9 | Male | 56 | Negative | III | Left paramedian sector | T3 | N0 | M0 | 3A |
| Patient_10 | Female | 77 | Positive | II | Right paramedian sector | T3 | N0 | M0 | 3A |
| Patient_11 | Male | 67 | Negative | III | Right paramedian sector | T3 | N0 | M0 | 3A |

**Table S2.** Antibodies used in this study.

| **REAGENT or RESOURCE** | **SOURCE** | **IDENTIFIER** |
| --- | --- | --- |
| Acetylated-Lysine Antibody | CST | Cat # 9441 |
| Anti-FLAP antibody | Abcam | Cat # ab314653 |
| beta Actin Monoclonal Antibody | Invitrogen | Cat # MA1-140 |
| BD OptiBuild™ BV650 Rat Anti-Mouse IgG1 | BD biosciences | Cat # 742478 |
| BD Pharmingen™ Purified Mouse Anti-Human Granzyme K | BD biosciences | Cat # 567938 |
| B3GAT1 Polyclonal Antibody | Invitrogen | Cat # PA5-119947 |
| CBP/p300 Polyclonal Antibody | Invitrogen | Cat # PA5-99492 |
| CCL5 (RANTES) Recombinant Polyclonal Antibody | Invitrogen | Cat # 710001 |
| CD3 Monoclonal Antibody | Invitrogen | Cat # MA1-7630 |
| CD7 Monoclonal Antibody | Invitrogen | Cat # 14-0079-82 |
| CD56 Monoclonal Antibody | Invitrogen | Cat # MA5-11563 |
| CD90.1 (Thy-1.1) Monoclonal Antibody | Invitrogen | Cat # 14-0900-81 |
| CD357 (AITR/GITR) Chimeric Recombinant Rabbit Monoclonal Antibody | Invitrogen | Cat # MA5-47934 |
| CD357 (AITR/GITR) Monoclonal Antibody | Invitrogen | Cat # 14-5875-80 |
| Coronin 1A (D6K5B) XP® Rabbit mAb | CST | Cat # 92904 |
| CPEB2 Polyclonal Antibody | Invitrogen | Cat # PA5-113055 |
| Cystatin F Polyclonal Antibody | Invitrogen | Cat # PA5-103772 |
| DYKDDDDK Tag Monoclonal Antibody | Invitrogen | Cat # MA1-91878 |
| FCER1G Polyclonal Antibody | Invitrogen | Cat # PA5-115222 |
| Ferritin Heavy Chain Recombinant Rabbit Monoclonal Antibody | Invitrogen | Cat # 701934 |
| Ferritin Recombinant Rabbit Monoclonal Antibody | Invitrogen | Cat # MA5-32244 |
| Ferroportin Polyclonal Antibody | Invitrogen | Cat # PA5-22993 |
| FLAP Polyclonal Antibody | Invitrogen | Cat # PA5-18283 |
| FTMT Polyclonal Antibody | Invitrogen | Cat # PA5-30906 |
| GAPDH Loading Control Monoclonal Antibody | Invitrogen | Cat # MA5-15738 |
| Goat anti-Mouse IgG1 Cross-Adsorbed Secondary Antibody, Alexa Fluor™ 647 | Invitrogen | Cat # A-21240 |
| Goat anti-Rabbit IgG (H+L) Cross-Adsorbed Secondary Antibody, FITC | Invitrogen | Cat # F-2765 |
| Goat anti-Rabbit IgG (H+L) Highly Cross-Adsorbed Secondary Antibody, Alexa Fluor™ 790 | Invitrogen | Cat # A11369 |
| Goat anti-Rabbit IgG (H+L) Secondary Antibody, HRP | Invitrogen | Cat # 31460 |
| Goat anti-Rabbit IgG (H+L) Cross-Adsorbed Secondary Antibody, Alexa Fluor™ 488 | Invitrogen | Cat # A-11008 |
| Granzyme A Polyclonal Antibody | Invitrogen | Cat # PA5-96539 |
| Granzyme K Polyclonal Antibody | Invitrogen | Cat # PA5-50980 |
| HA Tag Monoclonal Antibody | Invitrogen | Cat # 26183 |
| HDAC1 Polyclonal Antibody | Invitrogen | Cat # PA1-860 |
| HDAC9 Polyclonal Antibody | Invitrogen | Cat # PA5-23346 |
| Histone H3 Recombinant Polyclonal Antibody | Invitrogen | Cat # 710546 |
| HSC70 Polyclonal Antibody | Invitrogen | Cat # PA5-24624 |
| HSPA9 Polyclonal Antibody | Invitrogen | Cat # PA5-79410 |
| HSPE1 Polyclonal Antibody | Invitrogen | Cat # PA5-79415 |
| HSP60 Monoclonal Antibody | Invitrogen | Cat # MA3-012 |
| HSP70 Monoclonal Antibody | Invitrogen | Cat # MA3-009 |
| HSP90 alpha Monoclonal Antibody | Invitrogen | Cat # MA5-17091 |
| HSP90 beta Polyclonal Antibody | Invitrogen | Cat # PA3-012 |
| HSP105 Polyclonal Antibody | Invitrogen | Cat # PA5-79417 |
| IFN gamma Monoclonal Antibody (B133.5) | Invitrogen | Cat # M701 |
| IFN gamma Monoclonal Antibody (XMG1.2) | Invitrogen | Cat # 13-7311-85 |
| Myc Tag Polyclonal Antibody | Invitrogen | Cat # PA1-981 |
| NEDD4L Monoclonal Antibody | Invitrogen | Cat # H00023327-M04 |
| NKG2A/C/E Monoclonal Antibody | Invitrogen | Cat # 16-5896-85 |
| NKG7 (E6S2A) Rabbit mAb | CST | Cat # 84835 |
| CST | CST | Cat # 51968 |
| NKp46 Polyclonal Antibody | Invitrogen | Cat # PA5-102860 |
| Phospho-PLCg2 (Tyr759) Recombinant Rabbit Monoclonal Antibody | Invitrogen | Cat # LA |
| p21 Monoclonal Antibody | Invitrogen | Cat # MA5-31479 |
| RAMP1 Polyclonal Antibody | Invitrogen | Cat # PA5-110265 |
| RAMP1 (extracellular) Polyclonal Antibody | Invitrogen | Cat # ARR-021 |
| Rat anti-Mouse IgG1 Secondary Antibody, PE | Invitrogen | Cat # 12-4015-82 |
| Rat anti-Mouse IgG2a Secondary Antibody, PerCP-eFluor™ 710 | Invitrogen | Cat # 46-4210-82 |
| Recombinant Human XCL1 | Biolegend | Cat # 758002 |
| Recombinant Mouse XCL1 | Biolegend | Cat # 783502 |
| RFX7 Antibody | Novus | Cat # NBP1-71819 |
| ROR gamma (t) Monoclonal Antibody | Invitrogen | Cat # 14-6988-82 |
| SIRT3 Monoclonal Antibody | Invitrogen | Cat # PA5-96406 |
| SLC11A2 Polyclonal Antibody | Invitrogen | Cat # PA5-35136 |
| SLC39A8 Polyclonal Antibody | Invitrogen | Cat # PA5-21073 |
| SMG1 Polyclonal Antibody | Invitrogen | Cat # PA5-115933 |
| STEAP3 Polyclonal Antibody | Invitrogen | Cat # PA5-20406 |
| Ultra-LEAF™ Purified anti-mouse CD52 Antibody | Biolegend | Cat # 165703 |
| ZIP14 Polyclonal Antibody | Invitrogen | Cat # PA5-21077 |
| 6x-His Tag Monoclonal Antibody | Invitrogen | Cat # MA1-21315 |

**Supplementary References**

1. Chen KP, Hsu CL, Oyang YJ, Huang HC, Juan HF. BIC: a database for the transcriptional landscape of bacteria in cancer. Nucleic Acids Res. 2023;51(D1):D1205-d11.

2. Newman AM, Liu CL, Green MR, Gentles AJ, Feng W, Xu Y, et al. Robust enumeration of cell subsets from tissue expression profiles. Nat Methods. 2015;12(5):453-7.

3. Finck R, Simonds EF, Jager A, Krishnaswamy S, Sachs K, Fantl W, et al. Normalization of mass cytometry data with bead standards. Cytometry A. 2013;83(5):483-94.

4. Hahne F, LeMeur N, Brinkman RR, Ellis B, Haaland P, Sarkar D, et al. flowCore: a Bioconductor package for high throughput flow cytometry. BMC Bioinformatics. 2009;10:106.

5. Finak G, Jiang W, Pardo J, Asare A, Gottardo R. QUAliFiER: an automated pipeline for quality assessment of gated flow cytometry data. BMC Bioinformatics. 2012;13:252.

6. Bendall SC, Simonds EF, Qiu P, Amir el AD, Krutzik PO, Finck R, et al. Single-cell mass cytometry of differential immune and drug responses across a human hematopoietic continuum. Science. 2011;332(6030):687-96.

7. Van Gassen S, Callebaut B, Van Helden MJ, Lambrecht BN, Demeester P, Dhaene T, et al. FlowSOM: Using self-organizing maps for visualization and interpretation of cytometry data. Cytometry A. 2015;87(7):636-45.

8. Cieslak MC, Castelfranco AM, Roncalli V, Lenz PH, Hartline DK. t-Distributed Stochastic Neighbor Embedding (t-SNE): A tool for eco-physiological transcriptomic analysis. Mar Genomics. 2020;51:100723.

9. Cheng Y, Wong MT, van der Maaten L, Newell EW. Categorical Analysis of Human T Cell Heterogeneity with One-Dimensional Soli-Expression by Nonlinear Stochastic Embedding. J Immunol. 2016;196(2):924-32.

10. Wang J, Wang J, Huang Y, Xiao Y. 3dRNA v2.0: An Updated Web Server for RNA 3D Structure Prediction. Int J Mol Sci. 2019;20(17).

11. Zhou X, Zheng W, Li Y, Pearce R, Zhang C, Bell EW, et al. I-TASSER-MTD: a deep-learning-based platform for multi-domain protein structure and function prediction. Nat Protoc. 2022;17(10):2326-53.

12. Eberhardt J, Santos-Martins D, Tillack AF, Forli S. AutoDock Vina 1.2.0: New Docking Methods, Expanded Force Field, and Python Bindings. J Chem Inf Model. 2021;61(8):3891-8.

13. Laskowski RA, Swindells MB. LigPlot+: multiple ligand-protein interaction diagrams for drug discovery. J Chem Inf Model. 2011;51(10):2778-86.

14. Nyberg WA, Ark J, To A, Clouden S, Reeder G, Muldoon JJ, et al. An evolved AAV variant enables efficient genetic engineering of murine T cells. Cell. 2023;186(2):446-60.e19.

15. Wang S, Meyer E, McKay JK, Matz MV. 2b-RAD: a simple and flexible method for genome-wide genotyping. Nat Methods. 2012;9(8):808-10.

16. Lindsay MR, D'Angelo T, Munson-McGee JH, Saidi-Mehrabad A, Devlin M, McGonigle J, et al. Species-resolved, single-cell respiration rates reveal dominance of sulfate reduction in a deep continental subsurface ecosystem. Proc Natl Acad Sci U S A. 2024;121(15):e2309636121.
