# Supplementary material for: Intratumoral Brevibacillus parabrevis enhances antitumor immunity by inhibiting NK cell ferroptosis in hepatocellular carcinoma
Source: Cell Death Dis. 2025 May 21;16(1):407. doi: 10.1038/s41419-025-07733-7 (PMC12095603; doi:10.1038/s41419-025-07733-7)

Fig. 2K

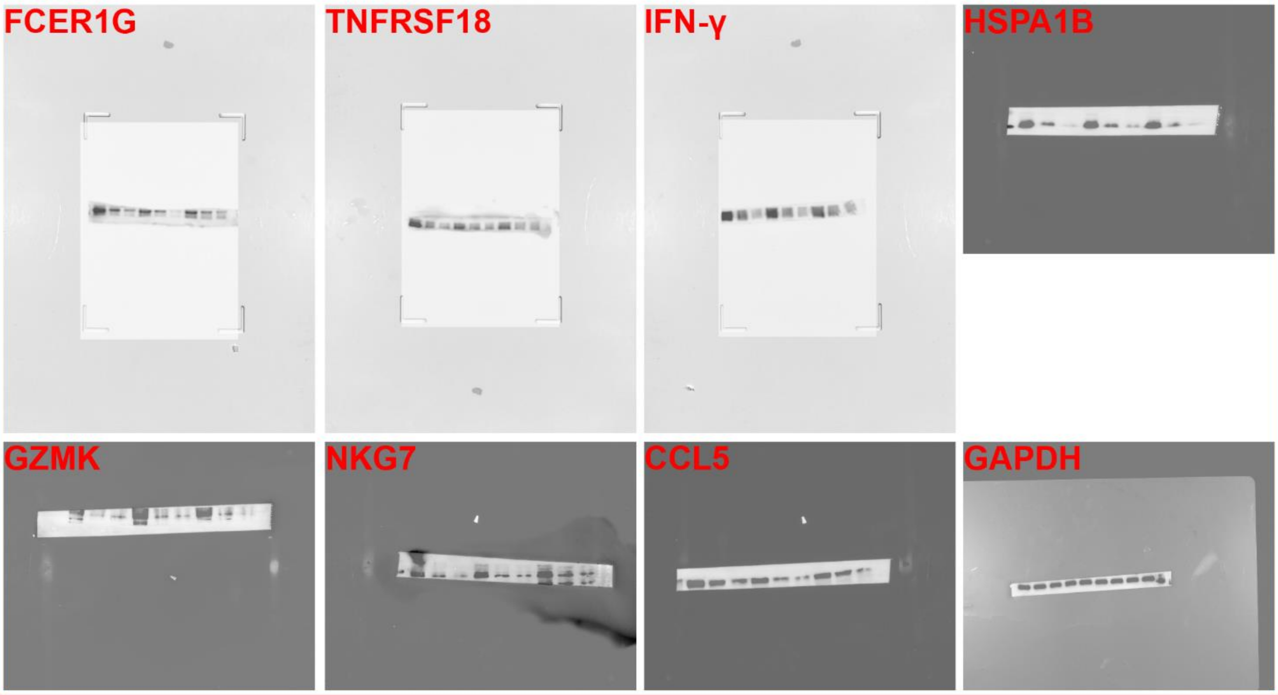
FCER1G
TNFRSF18
IFN-γ
HSPA1B
GZMK
NKG7
CCL5
GAPDH

Fig. 2L

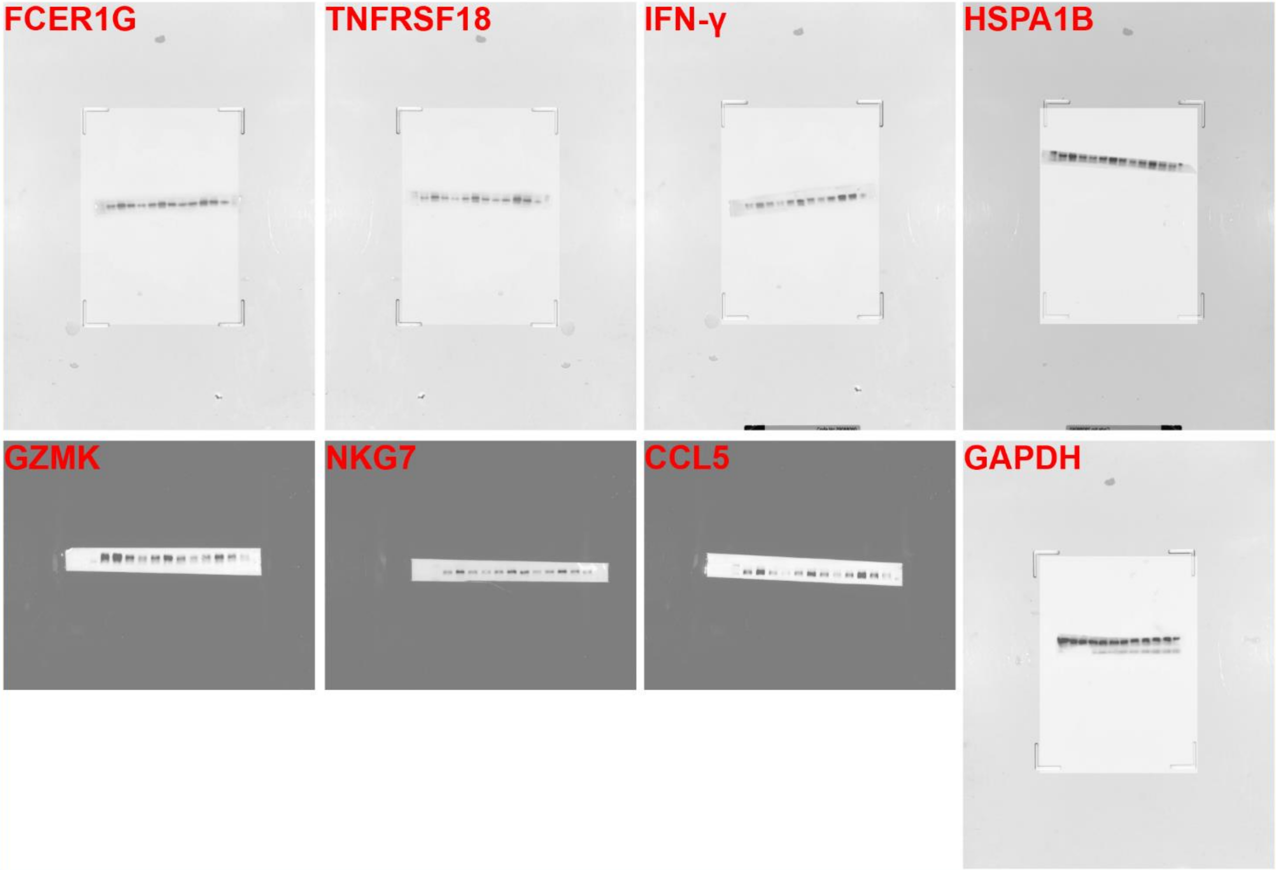
FCER1G
TNFRSF18
IFN-γ
HSPA1B
GZMK
NKG7
CCL5
GAPDH

Fig. 3A

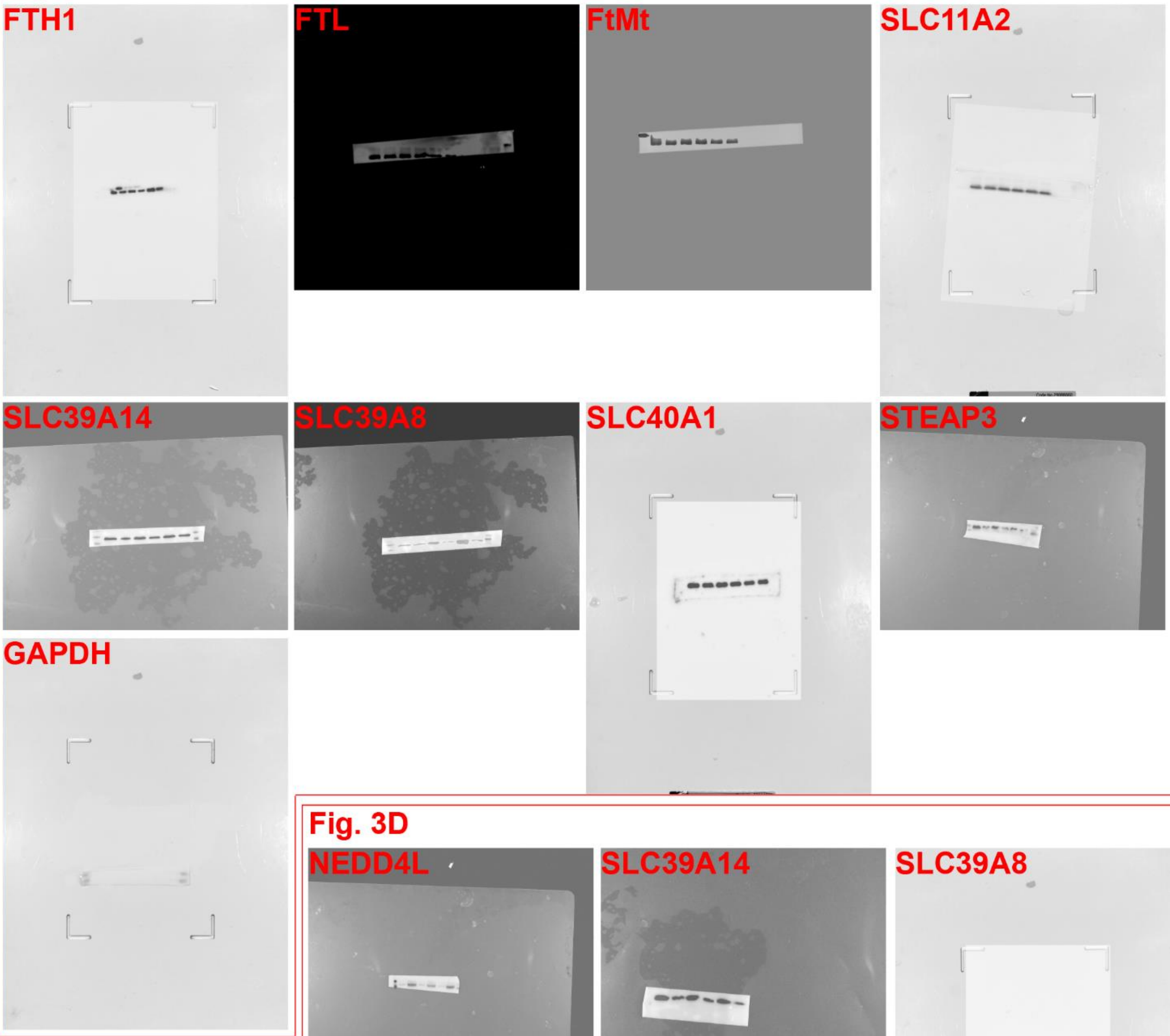
FTH1
FTL
FtMt
SLC11A2
SLC39A14
SLC39A8
SLC40A1
STEAP3
GAPDH

Fig. 3D

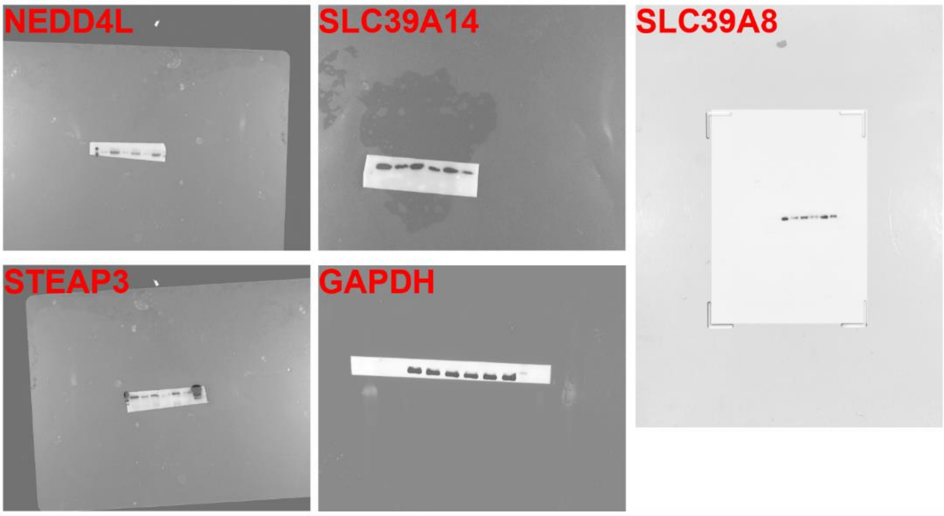
NEDD4L
SLC39A14
SLC39A8
STEAP3
GAPDH

Fig. 3E

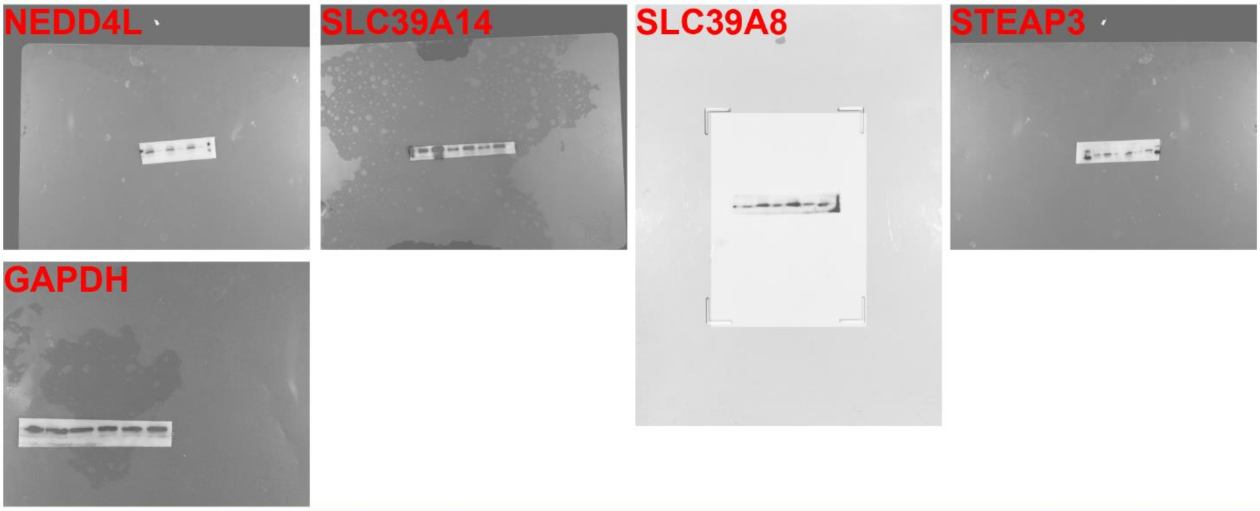

Fig. 3F

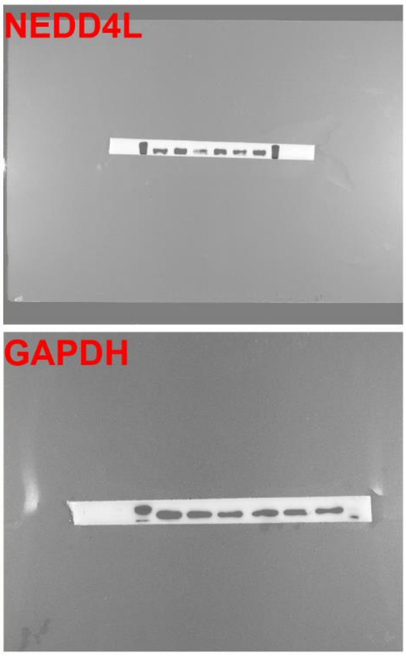

Fig. 3G

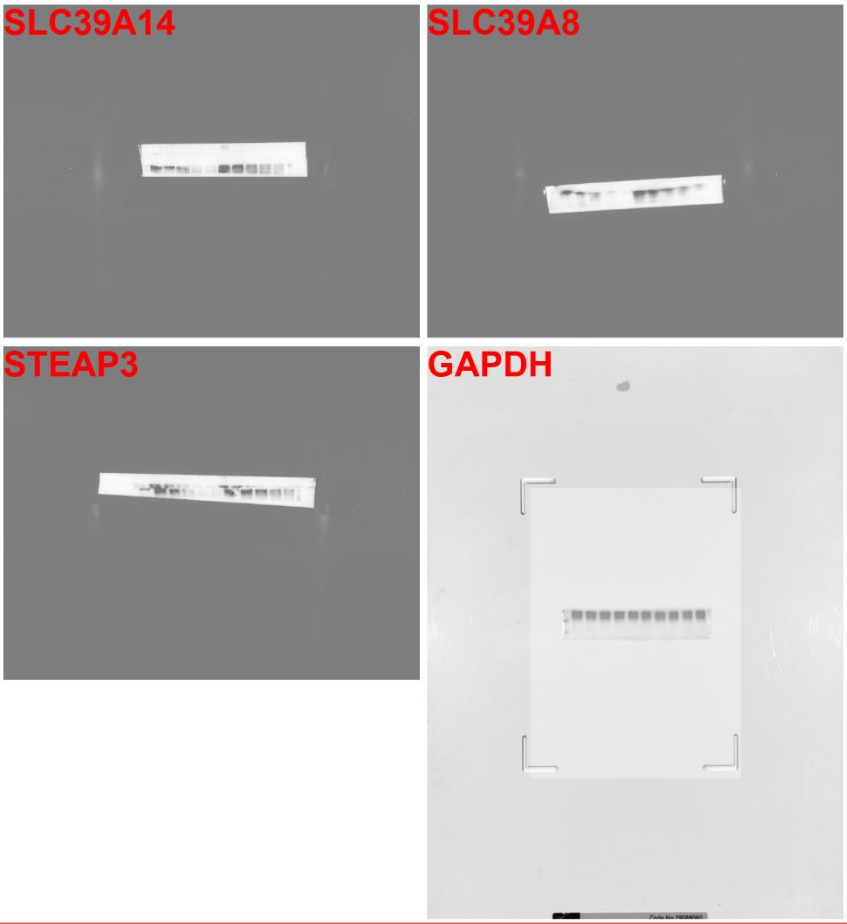

Fig. 4D

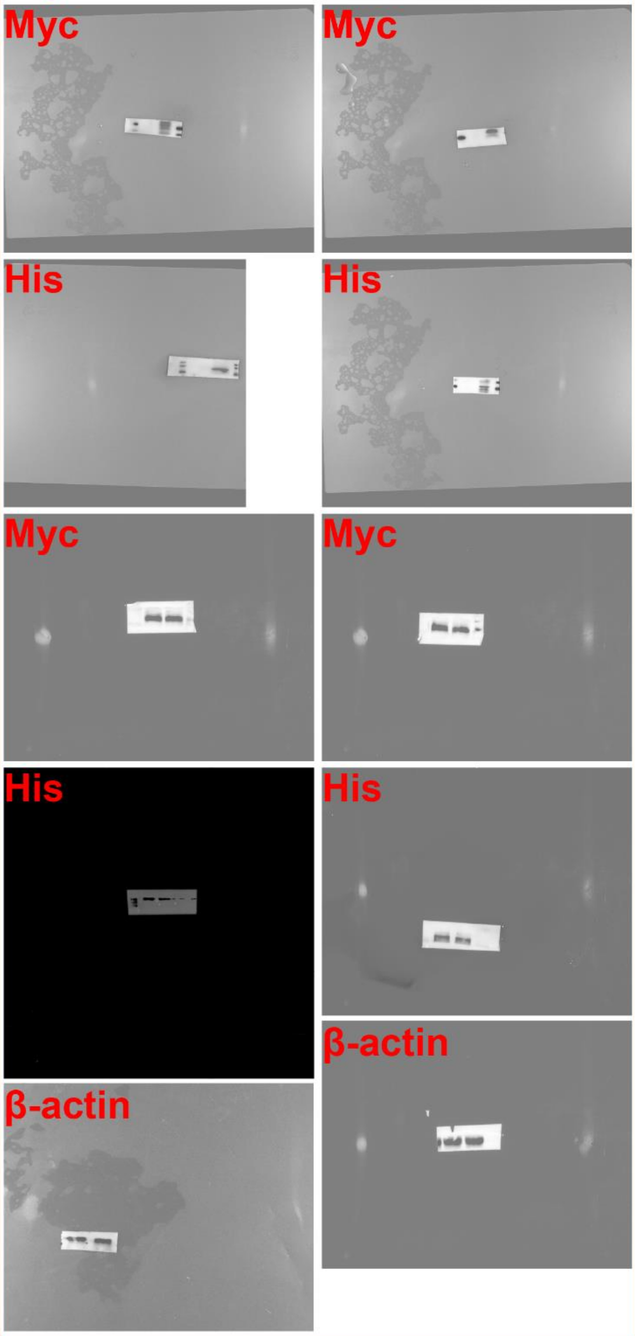
Myc
Myc
His
His
Myc
Myc
His
His
β-actin
β-actin

Fig. 4E

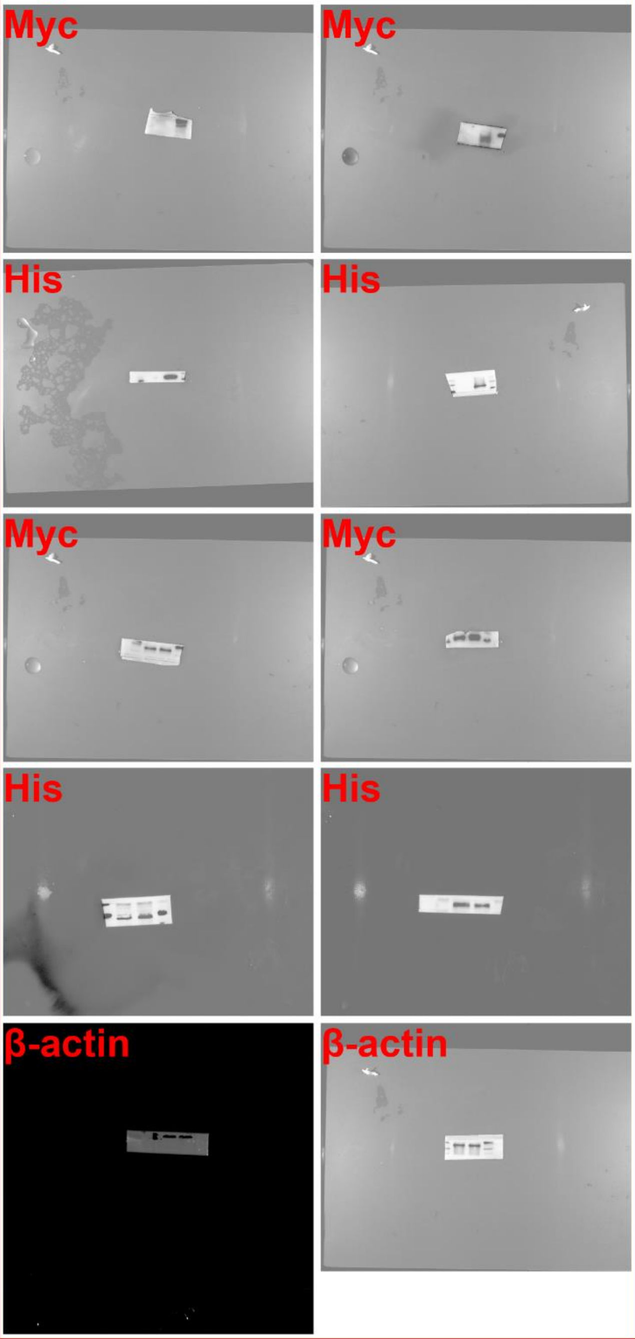
Myc
Myc
His
His
Myc
Myc
His
His
β-actin
β-actin

Fig. 4F

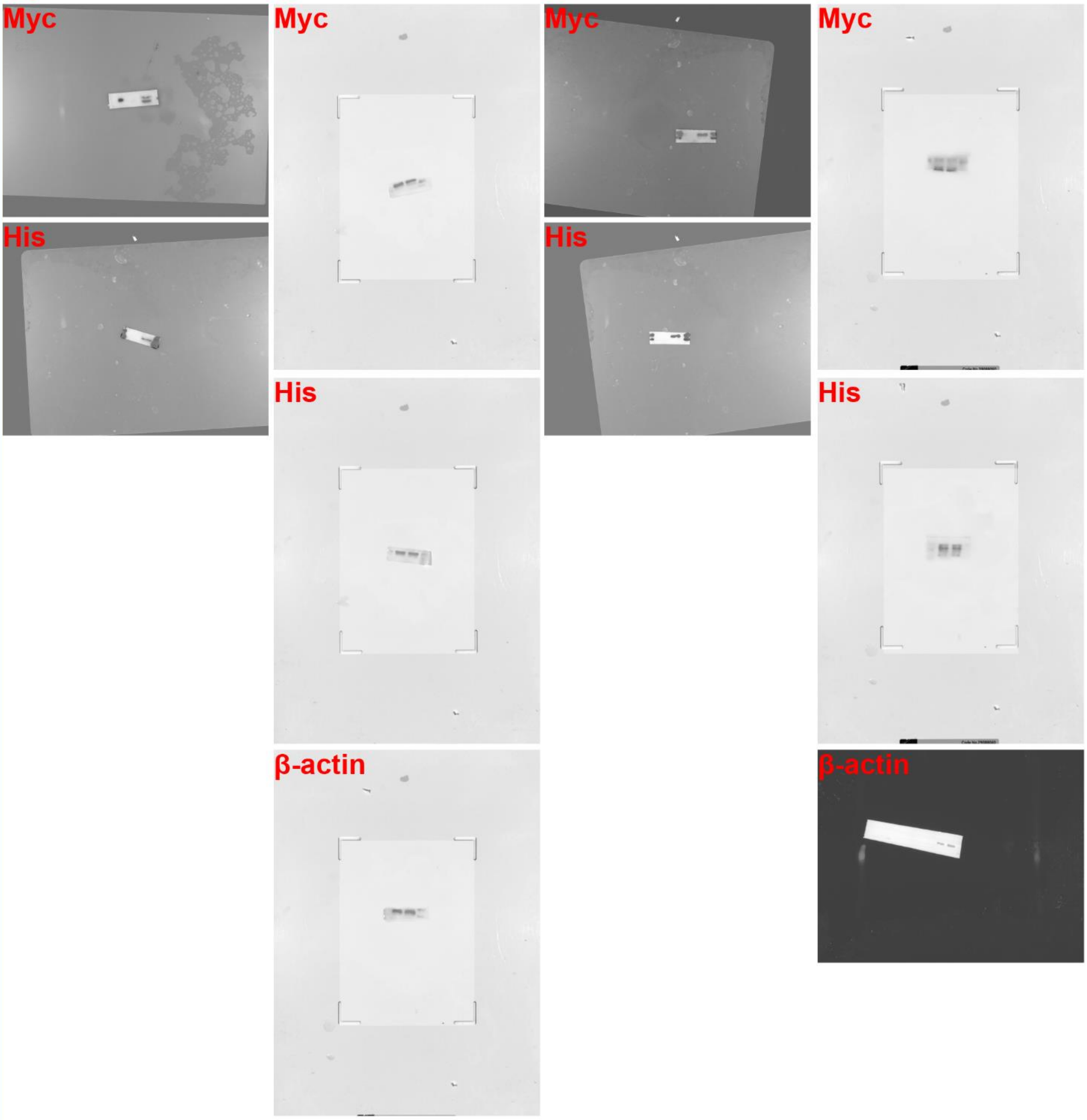

Fig. 4K

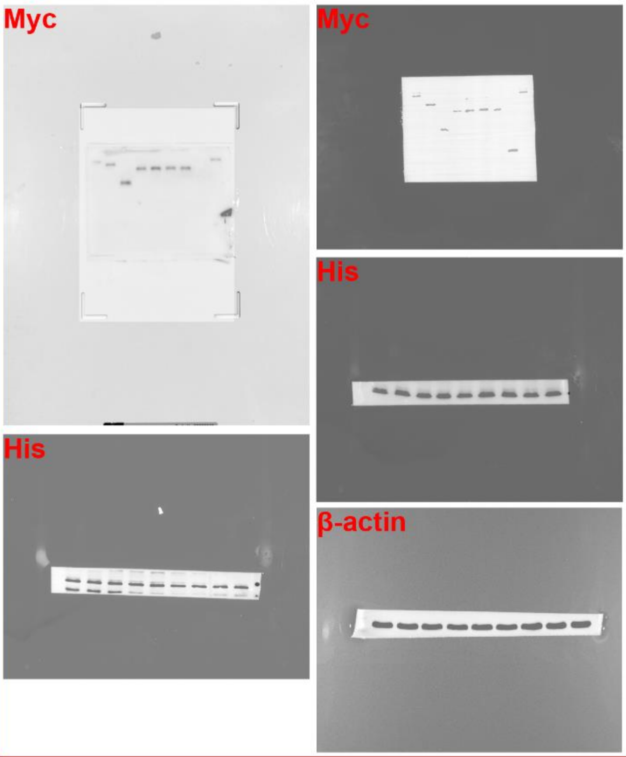

Fig. 4L

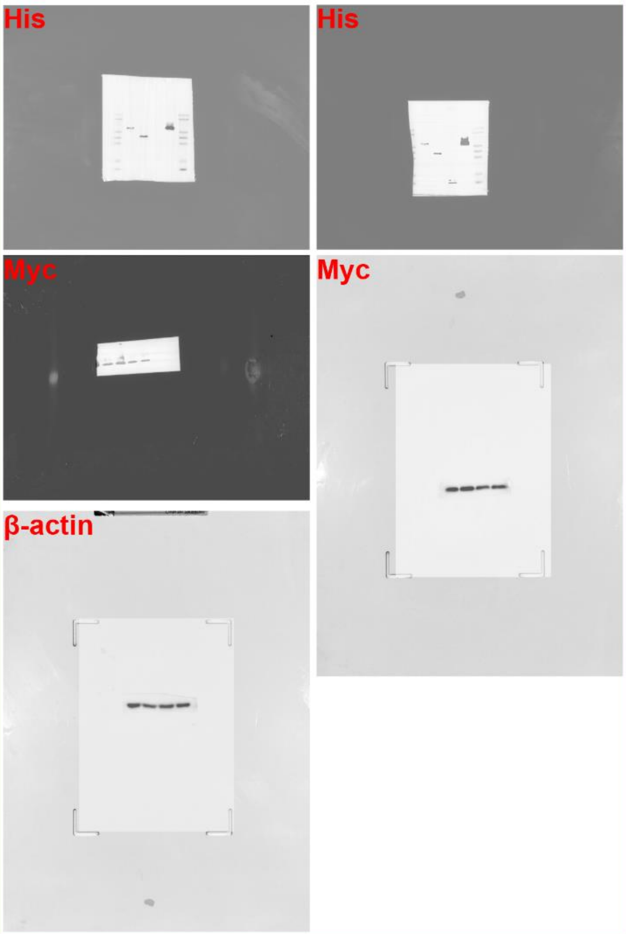

Fig. 4M

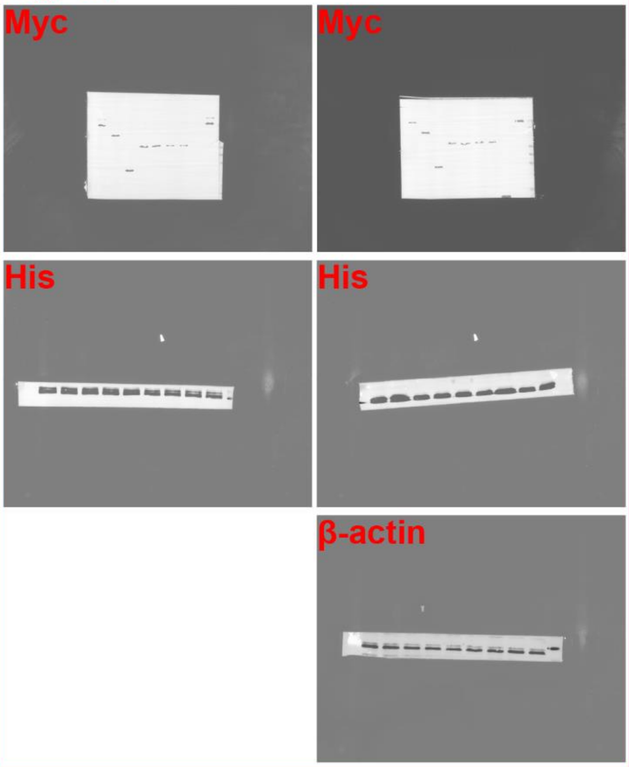

Fig. 4N

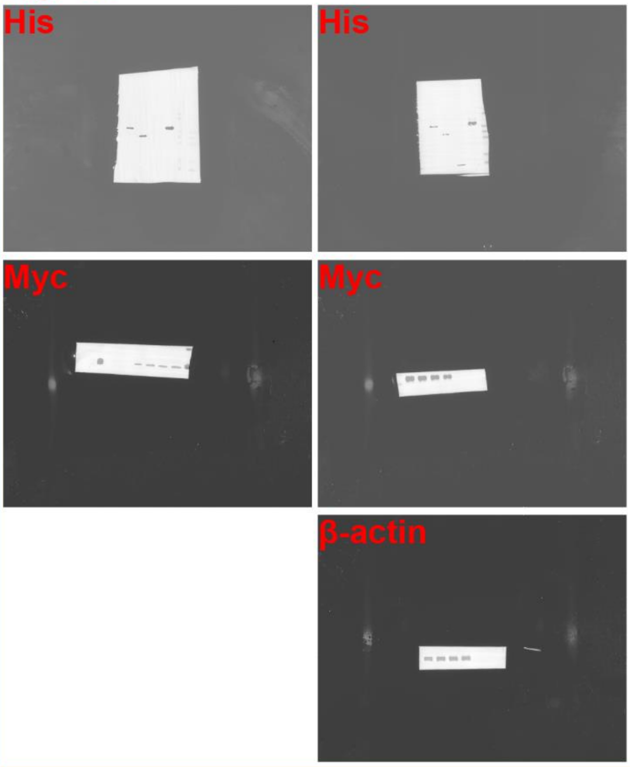

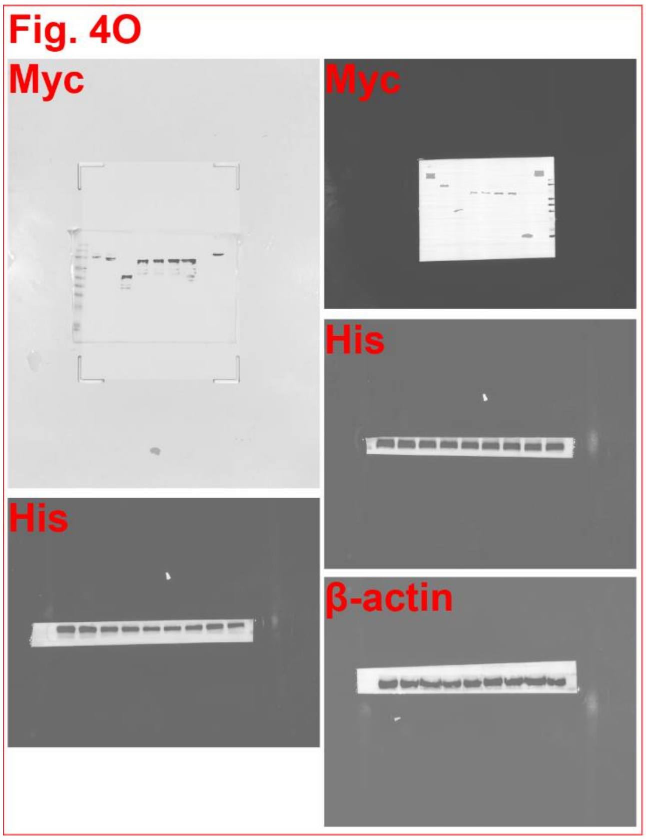
Fig. 4O
Myc
Myc
His
His
β-actin

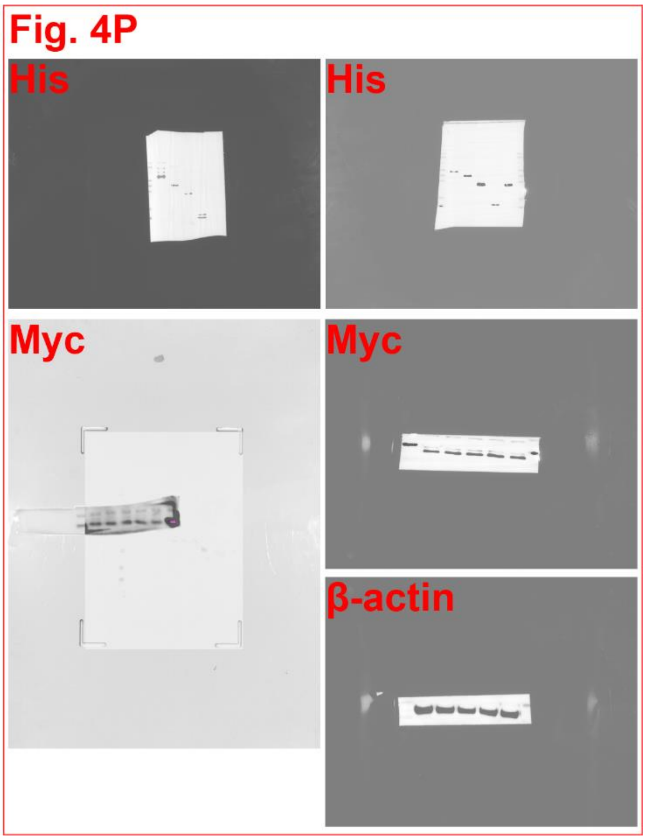
Fig. 4P
His
His
Myc
Myc
β-actin

Fig. 5A

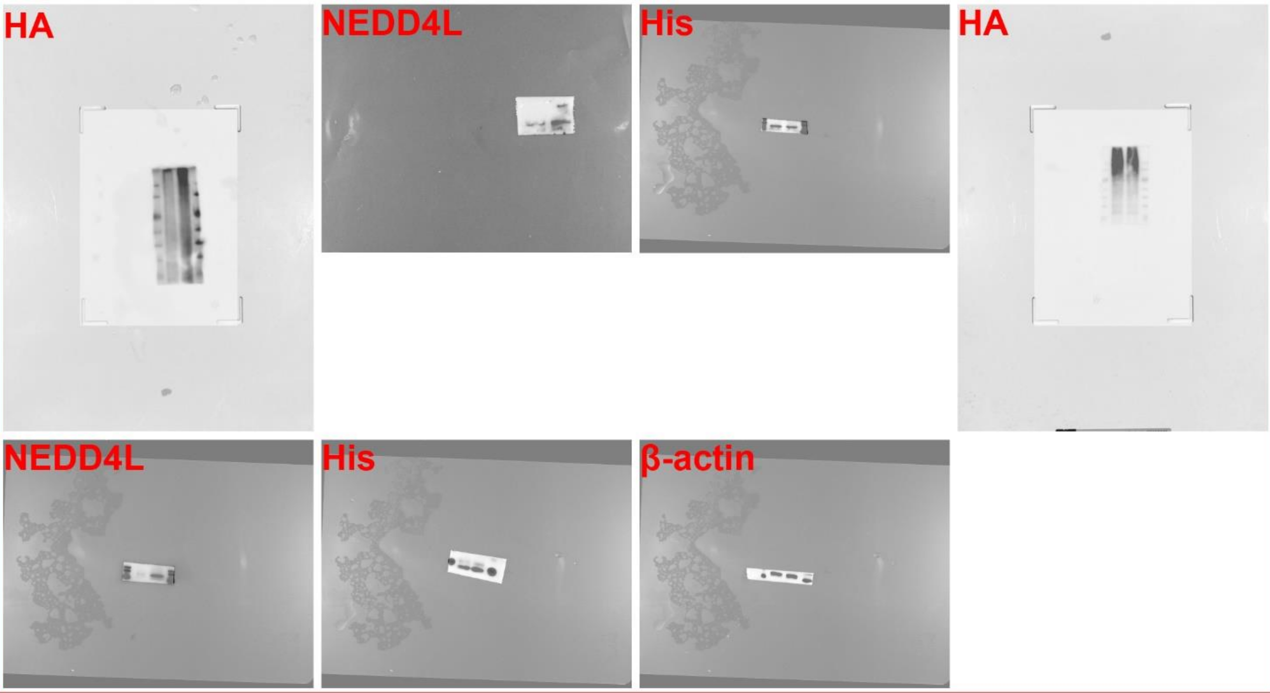
HA
NEDD4L
His
HA
NEDD4L
His
β-actin

Fig. 5B

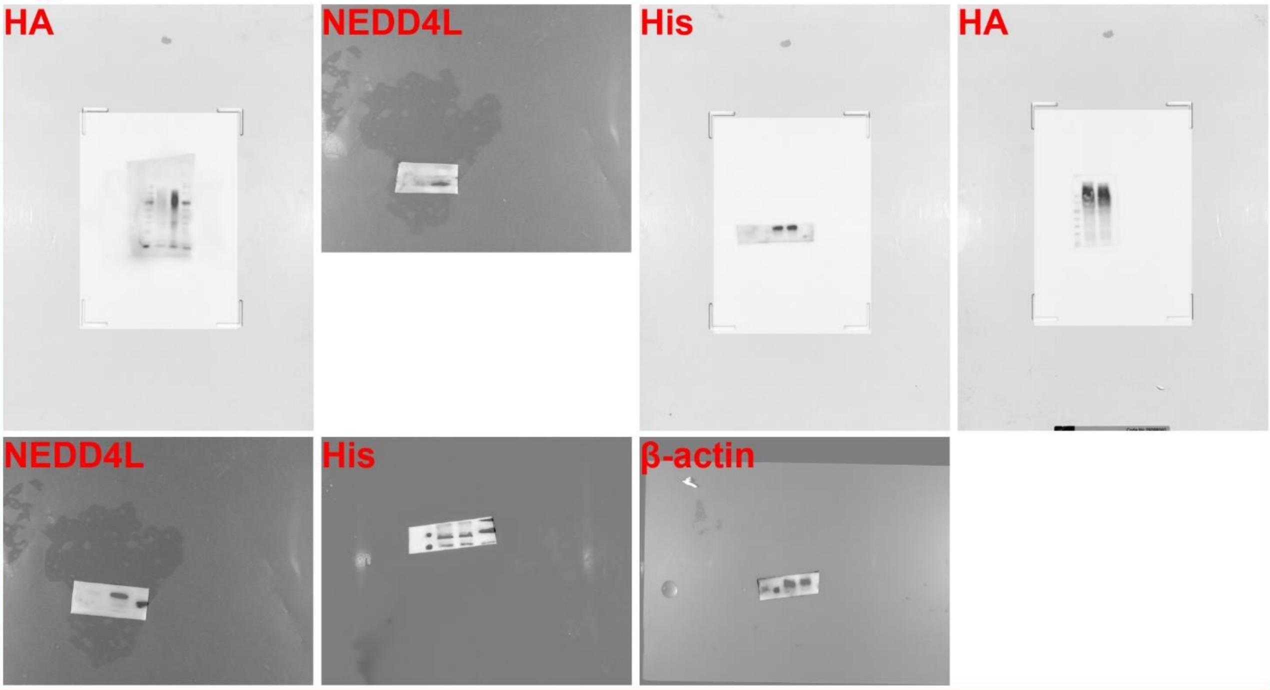
HA
NEDD4L
His
HA
NEDD4L
His
β-actin

Fig. 5C

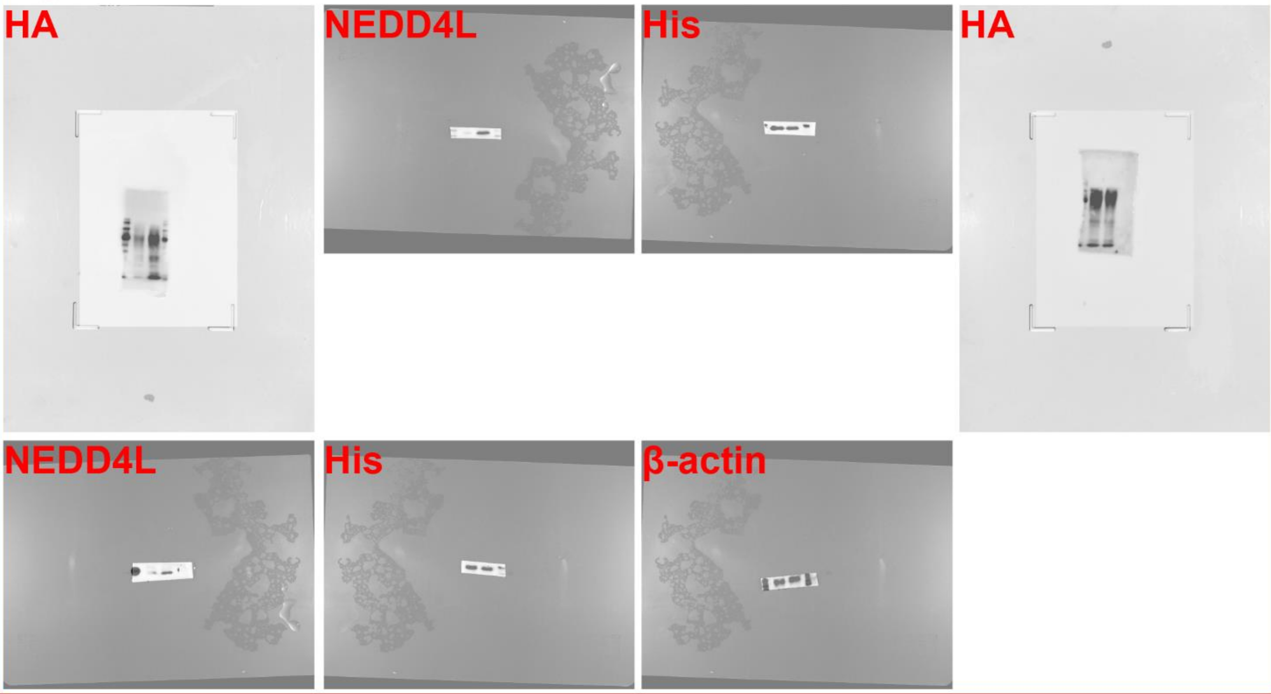
HA
NEDD4L
His
HA
NEDD4L
His
β-actin

Fig. 5D

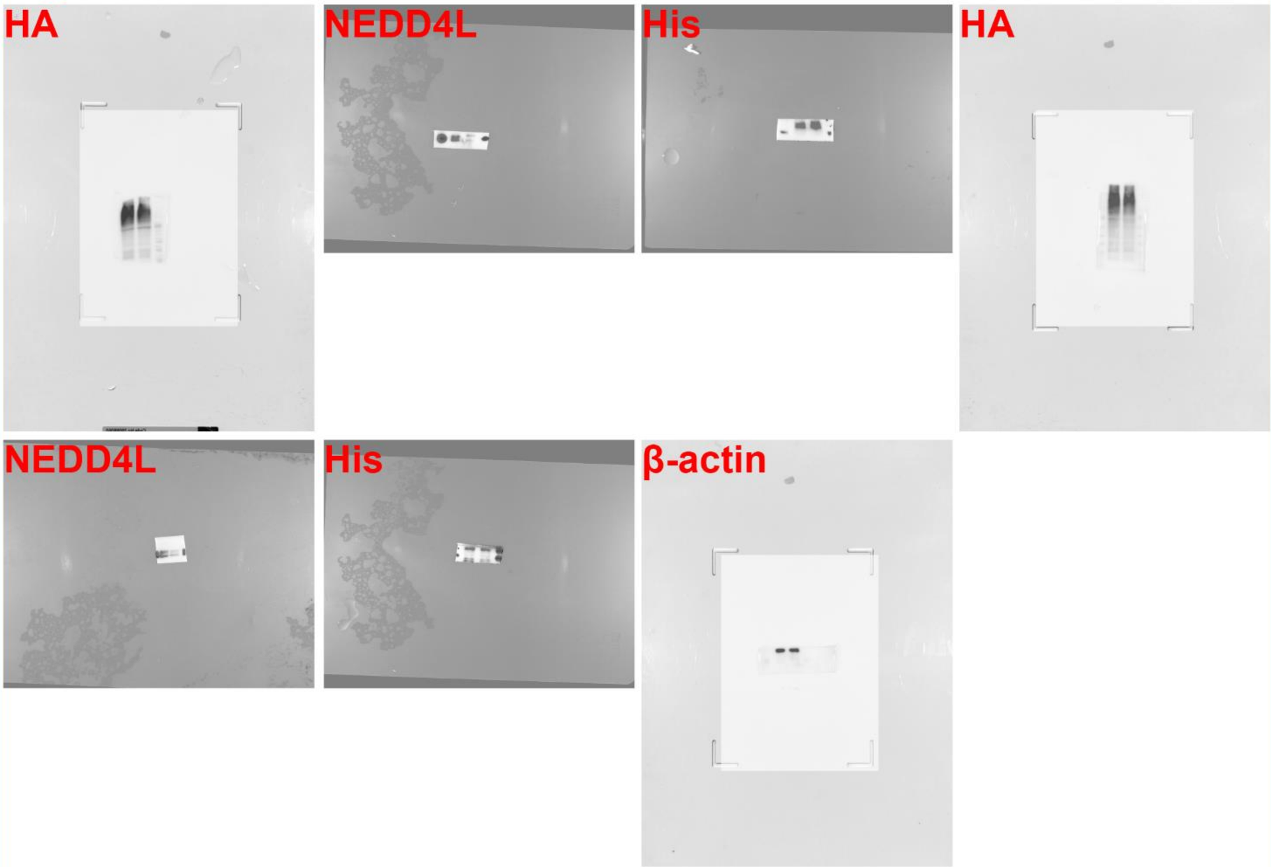
HA
NEDD4L
His
HA
NEDD4L
His
β-actin

Fig. 5E

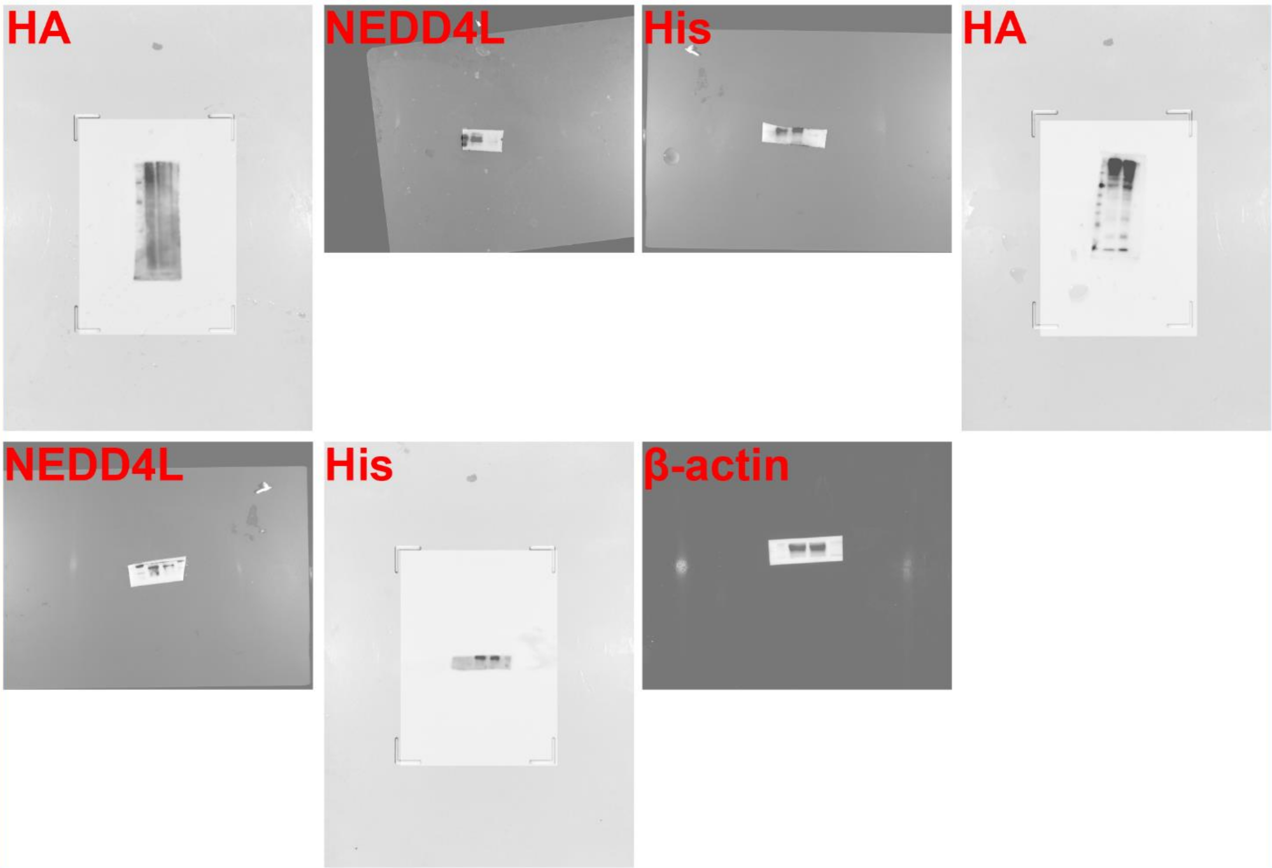
HA
NEDD4L
His
HA
NEDD4L
His
β-actin

Fig. 5F

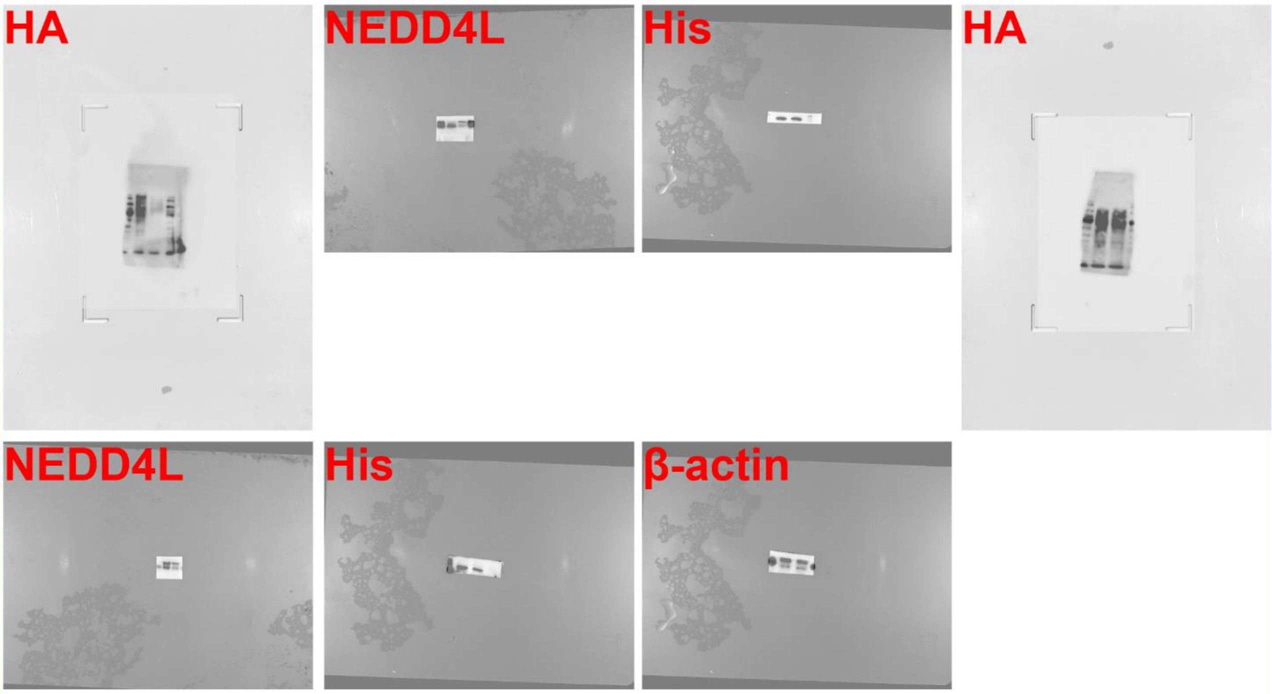
HA
NEDD4L
His
HA
NEDD4L
His
β-actin

Fig. 5G

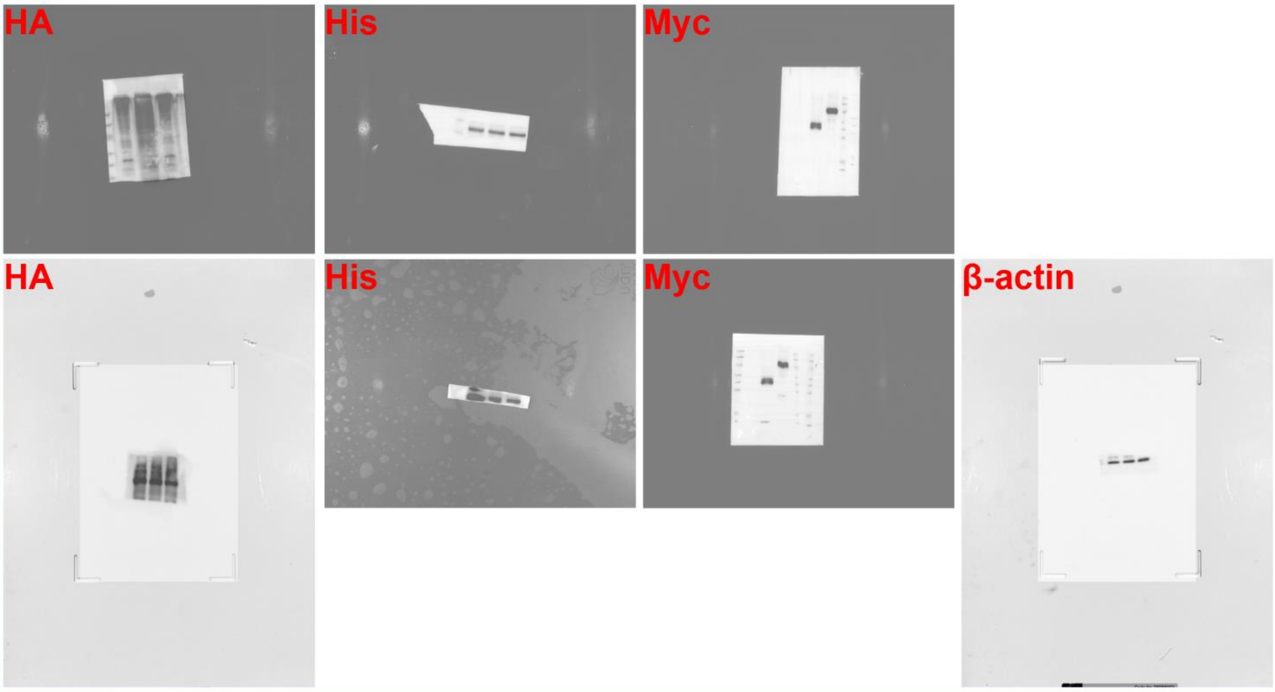

Fig. 5H

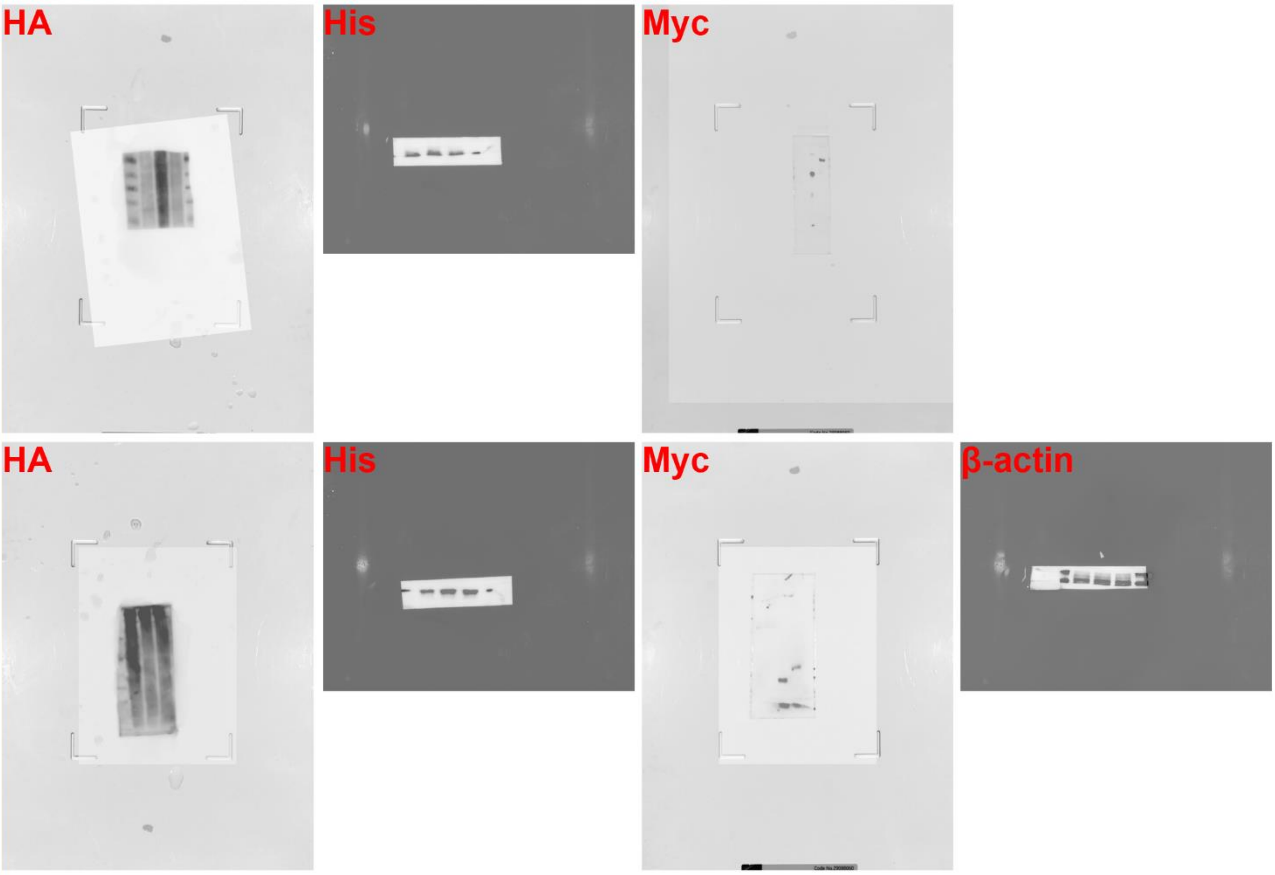

Fig. 5I

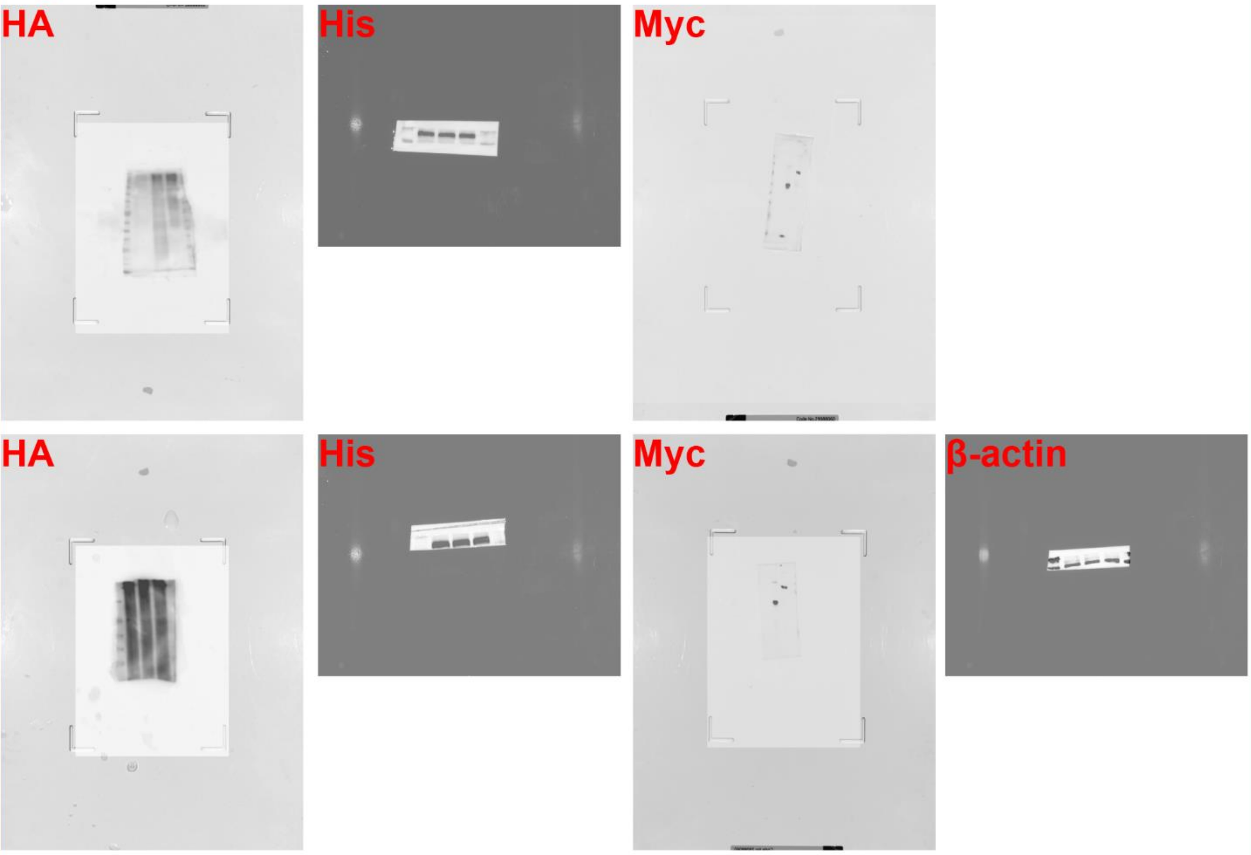

Fig. 5J

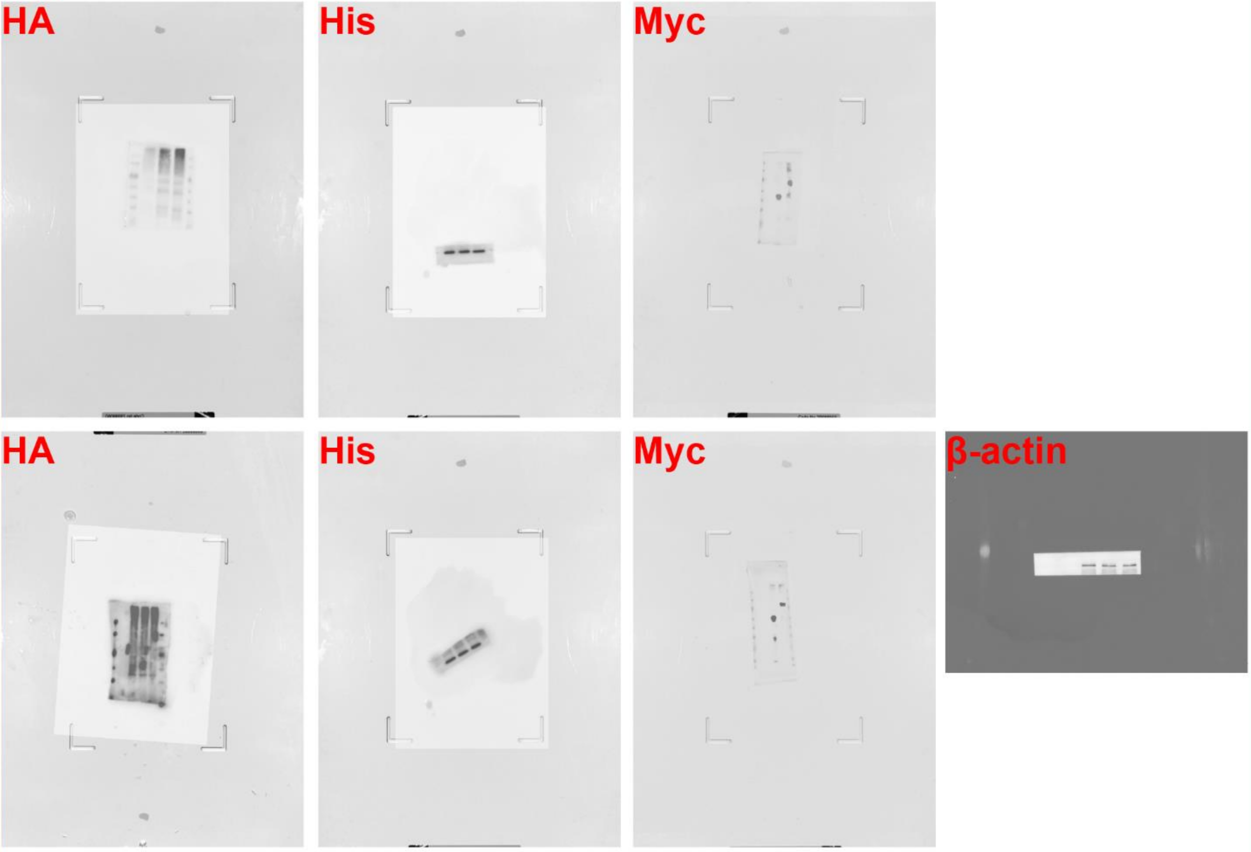

Fig. 5K

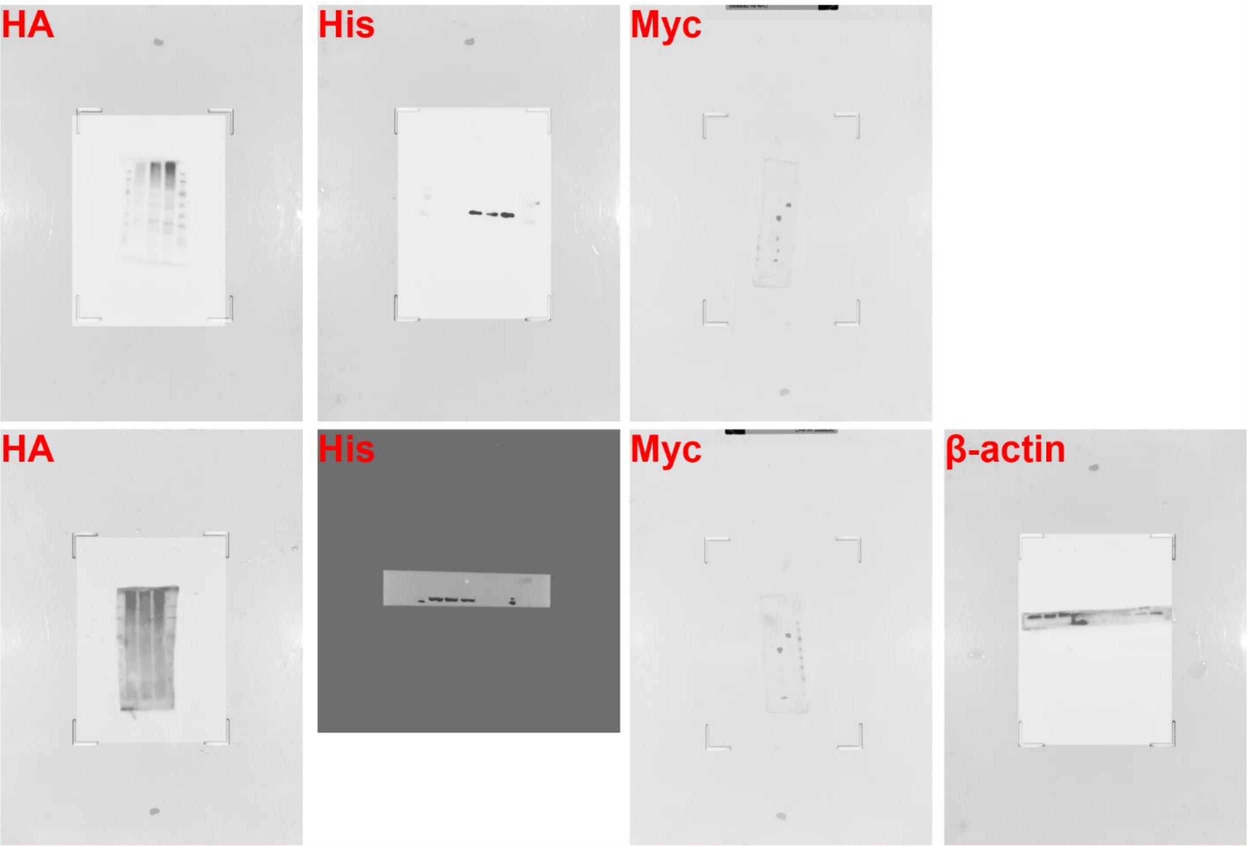

Fig. 5L

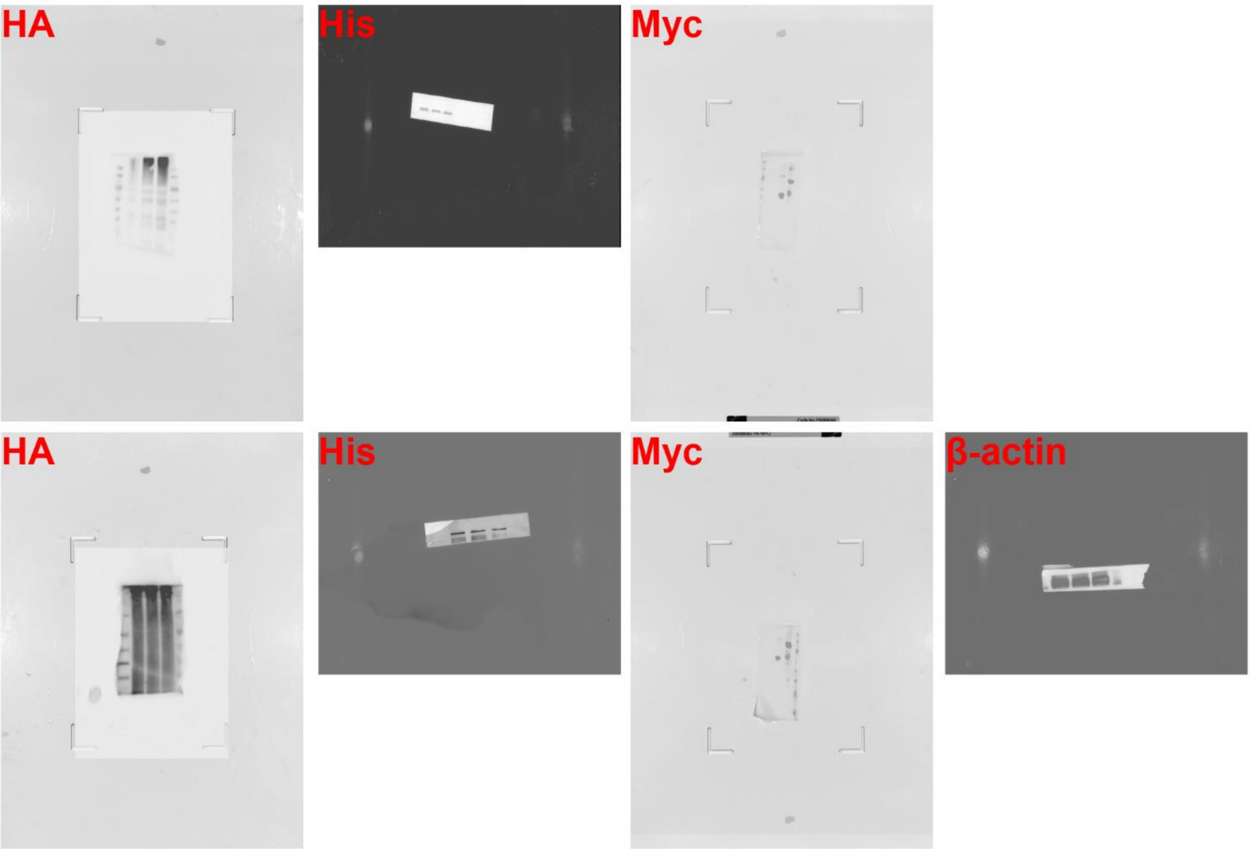

Fig. 5M

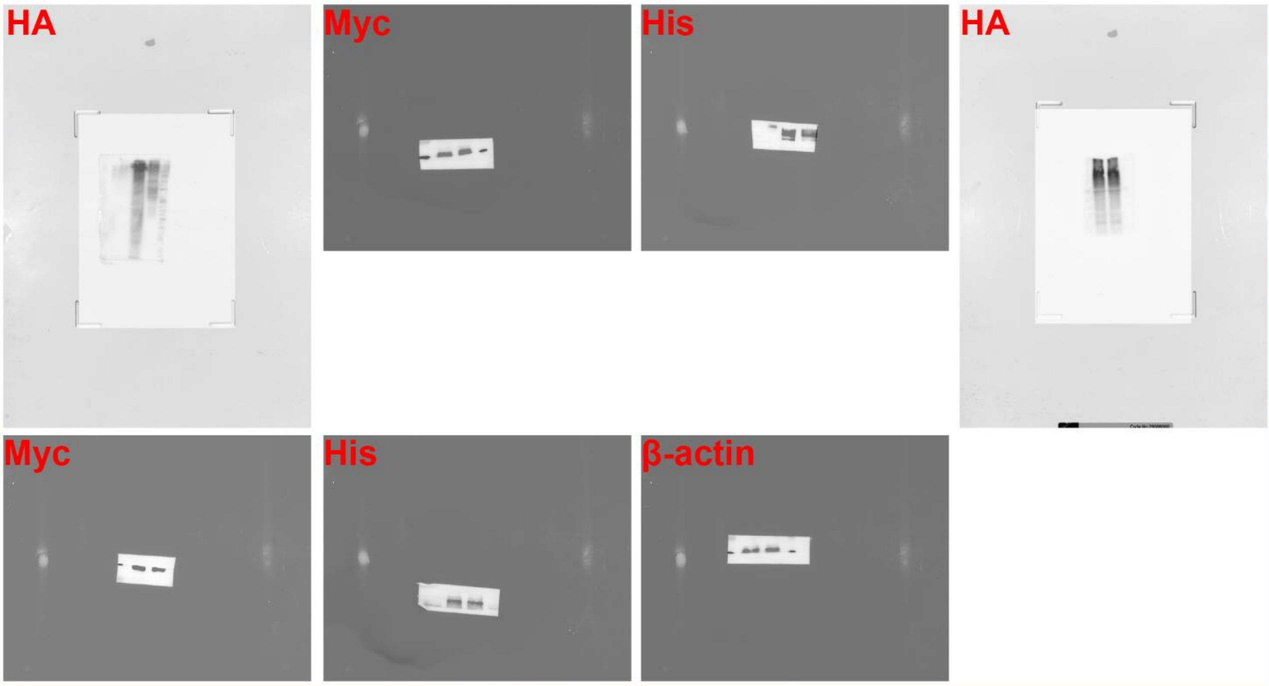

Fig. 5N

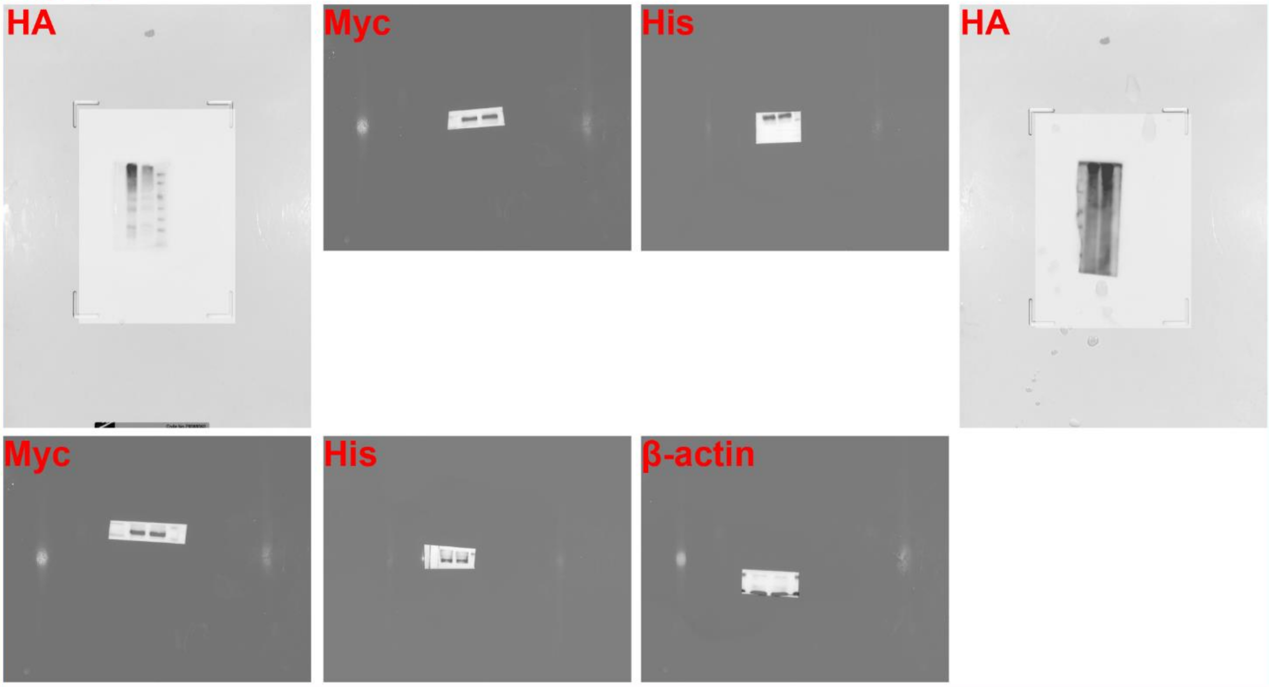

Fig. 5O

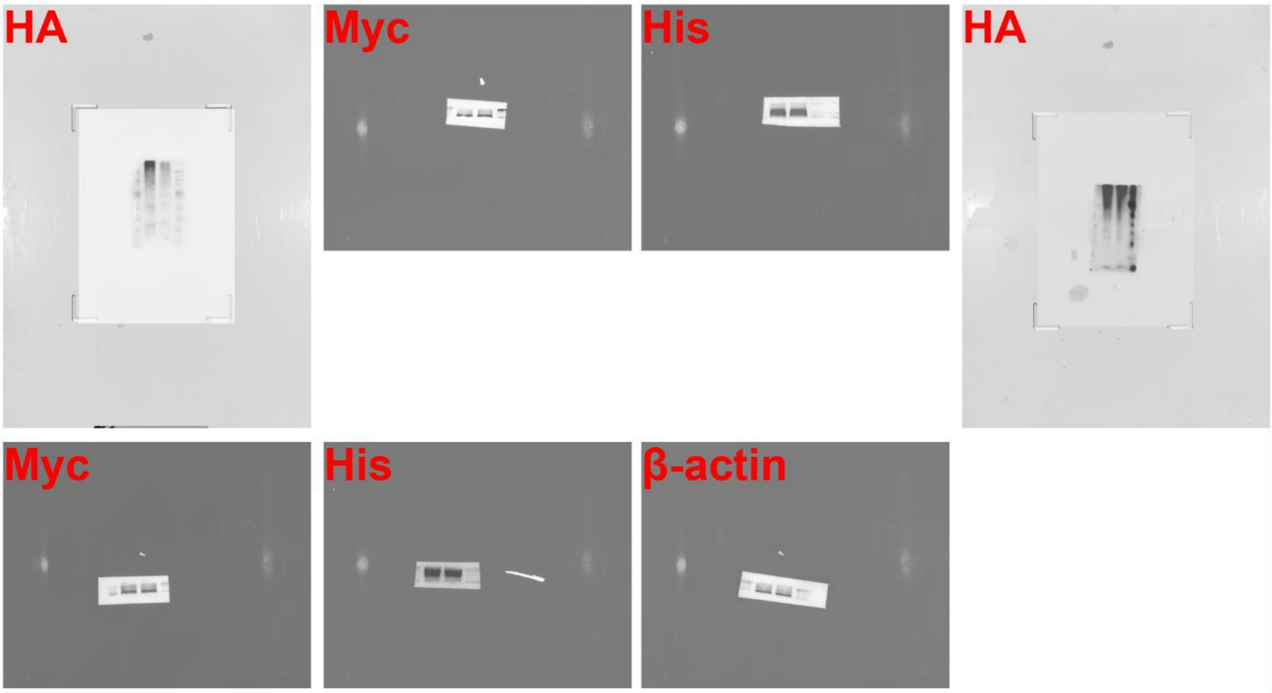

Fig. 5P

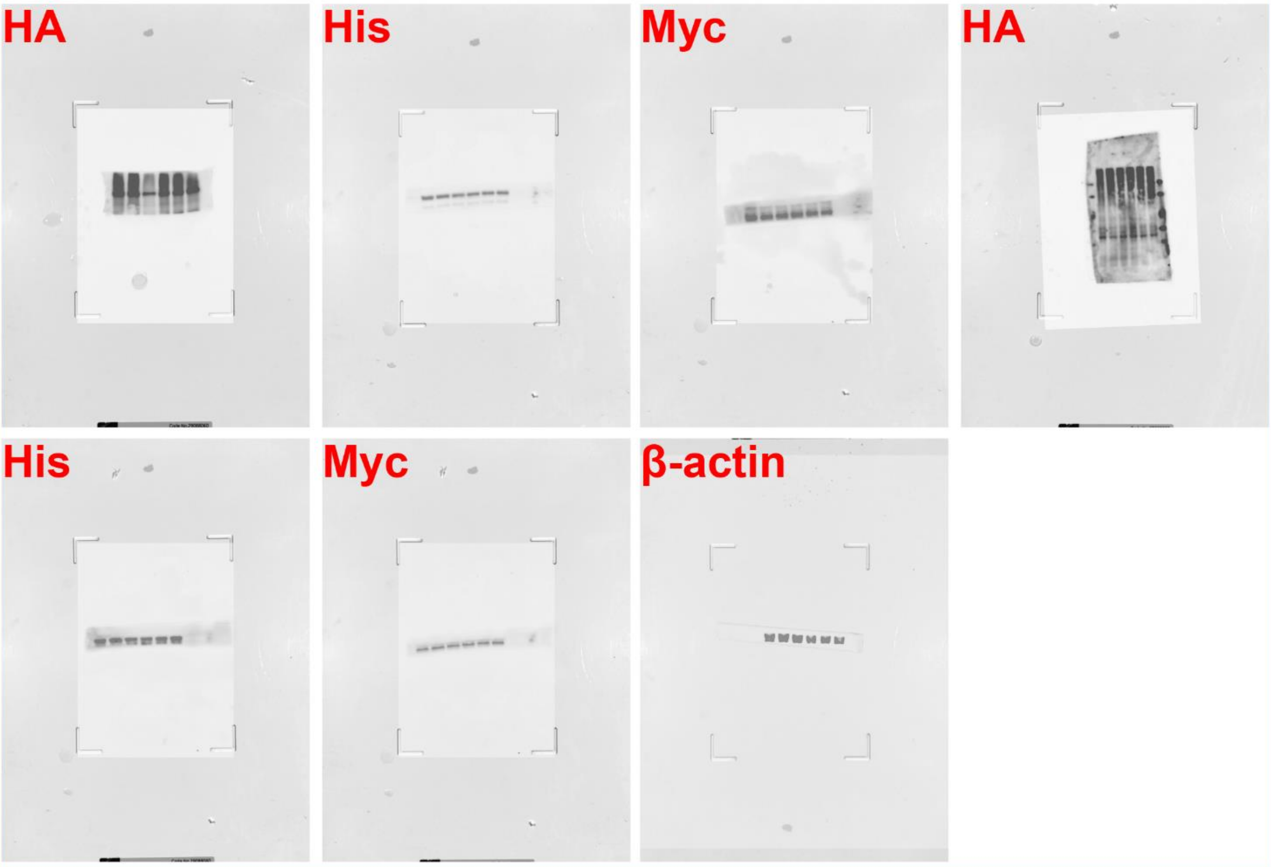

Fig. 5Q

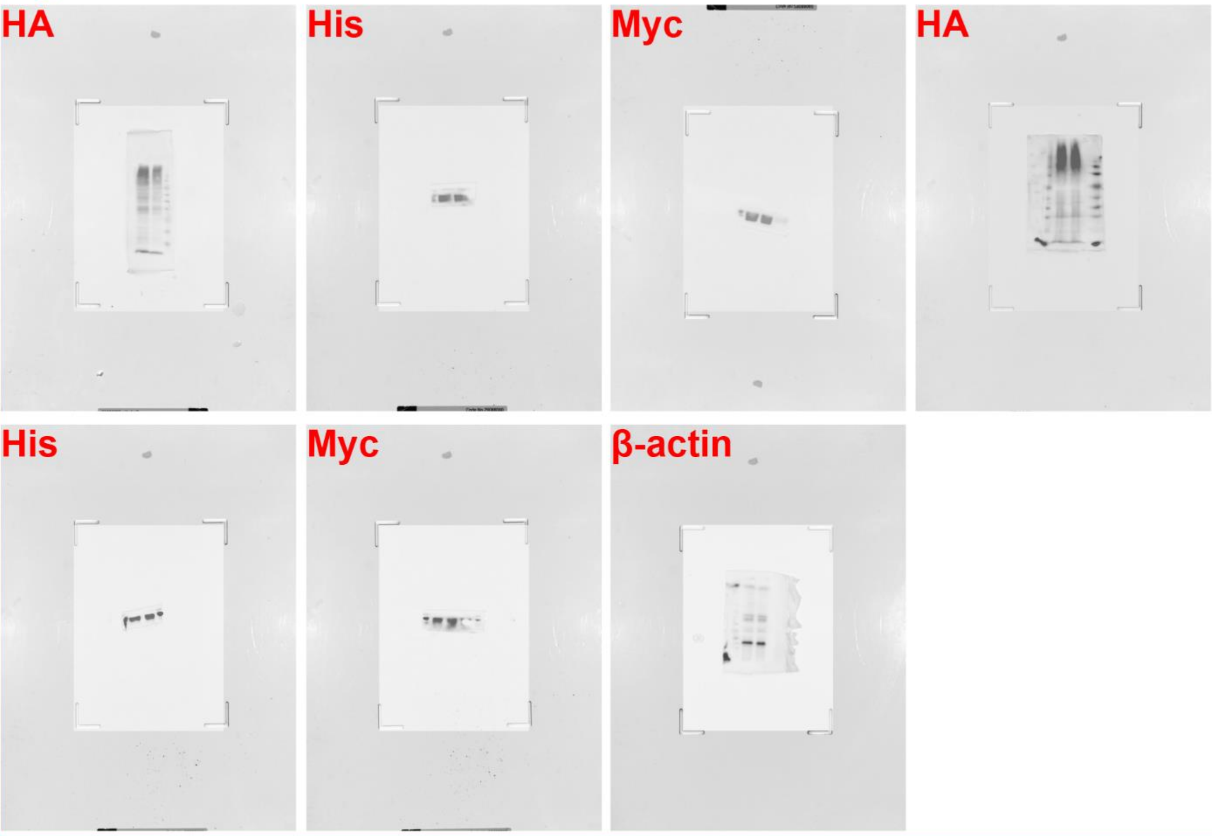

Fig. 5R

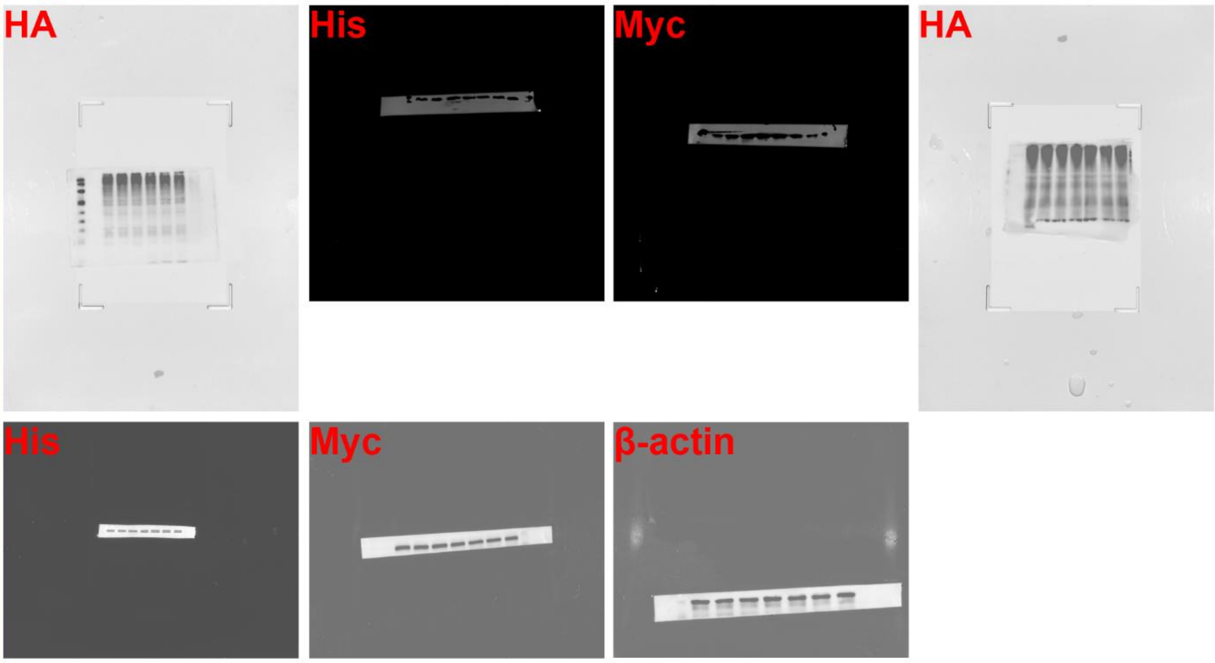

Fig. 5T

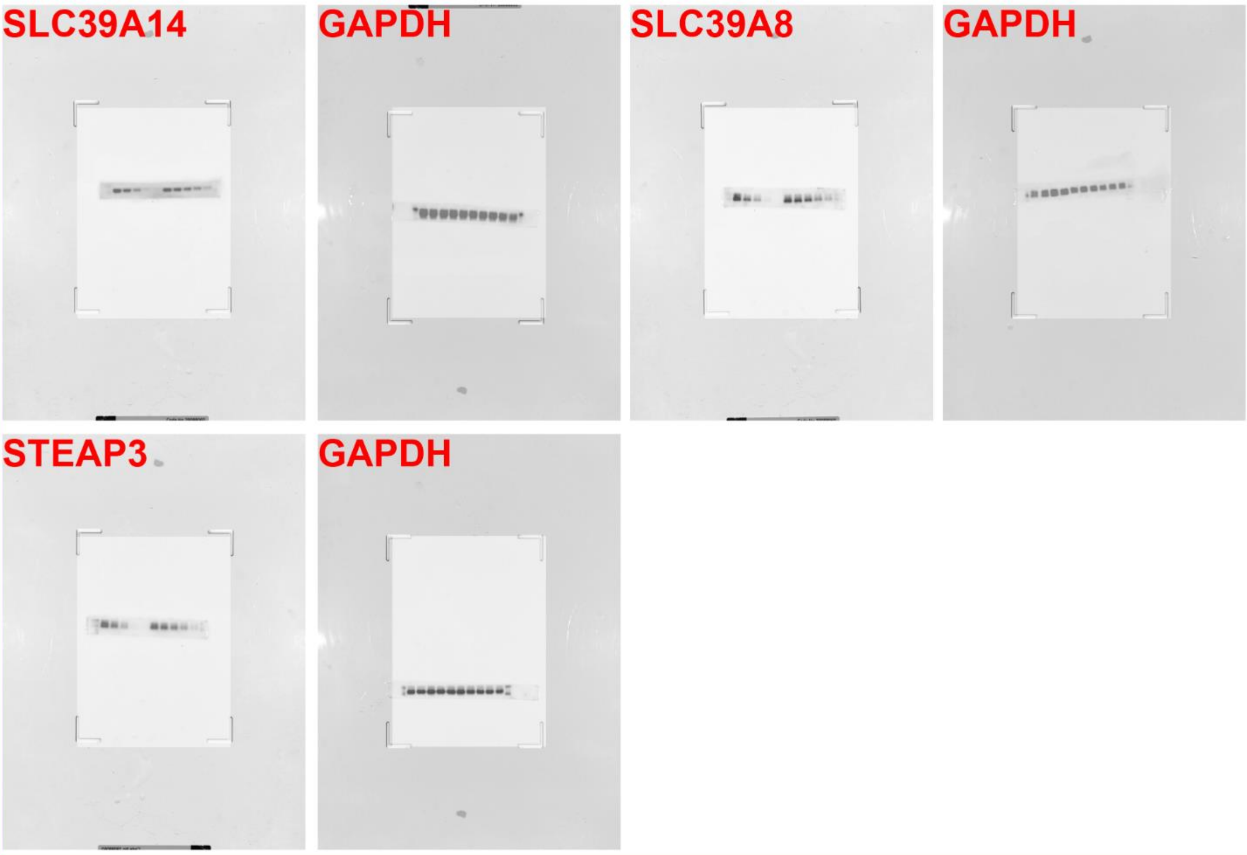
SLC39A14
GAPDH
SLC39A8
GAPDH
STEAP3
GAPDH

Fig. 5U

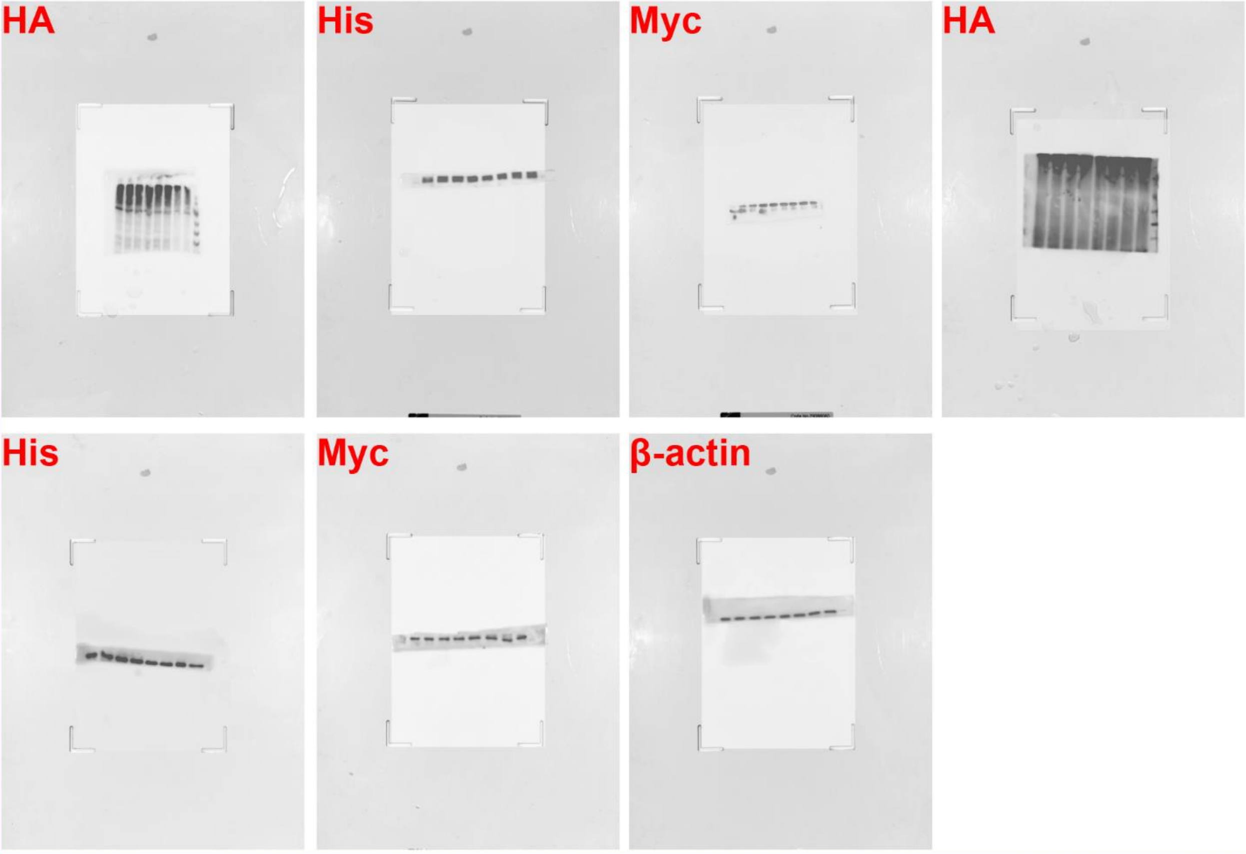
HA
His
Myc
HA
His
Myc
β-actin

Fig. 5V

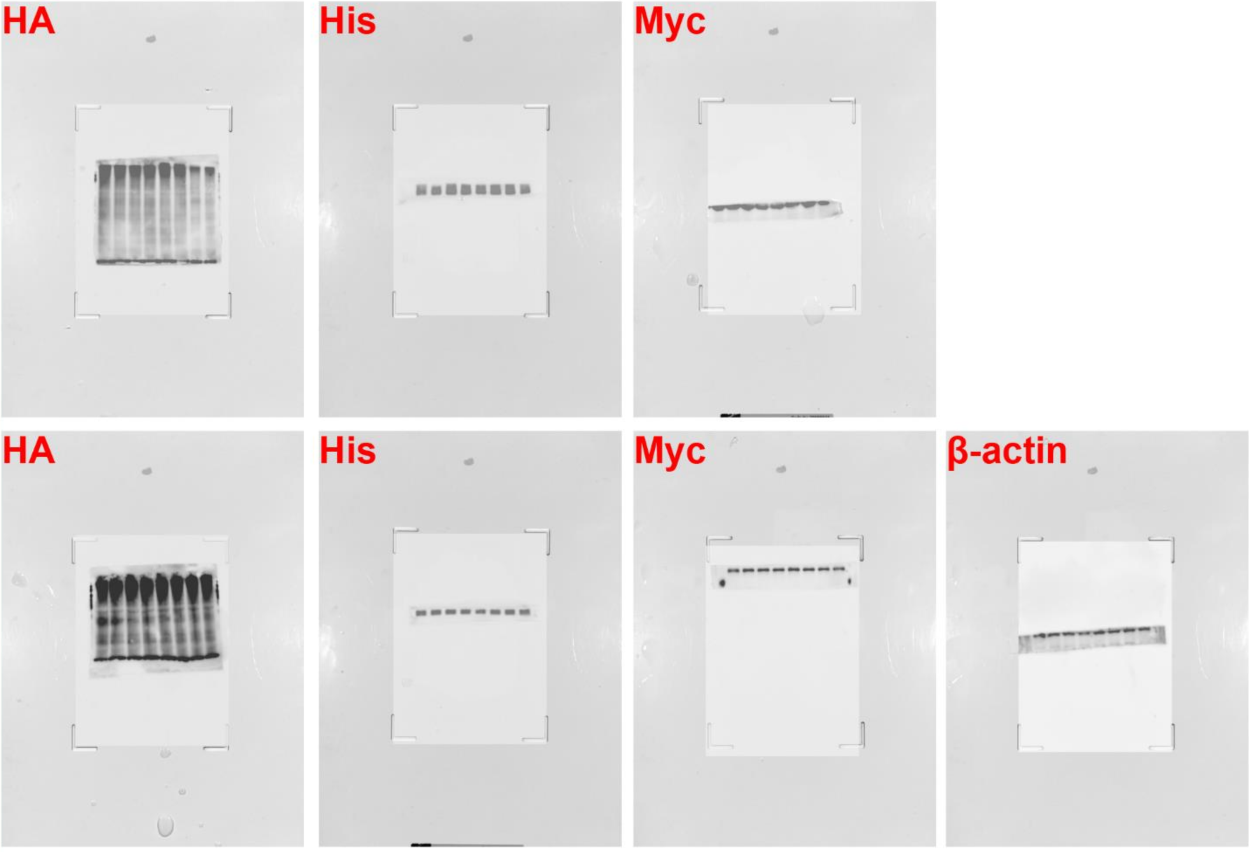

Fig. 5W

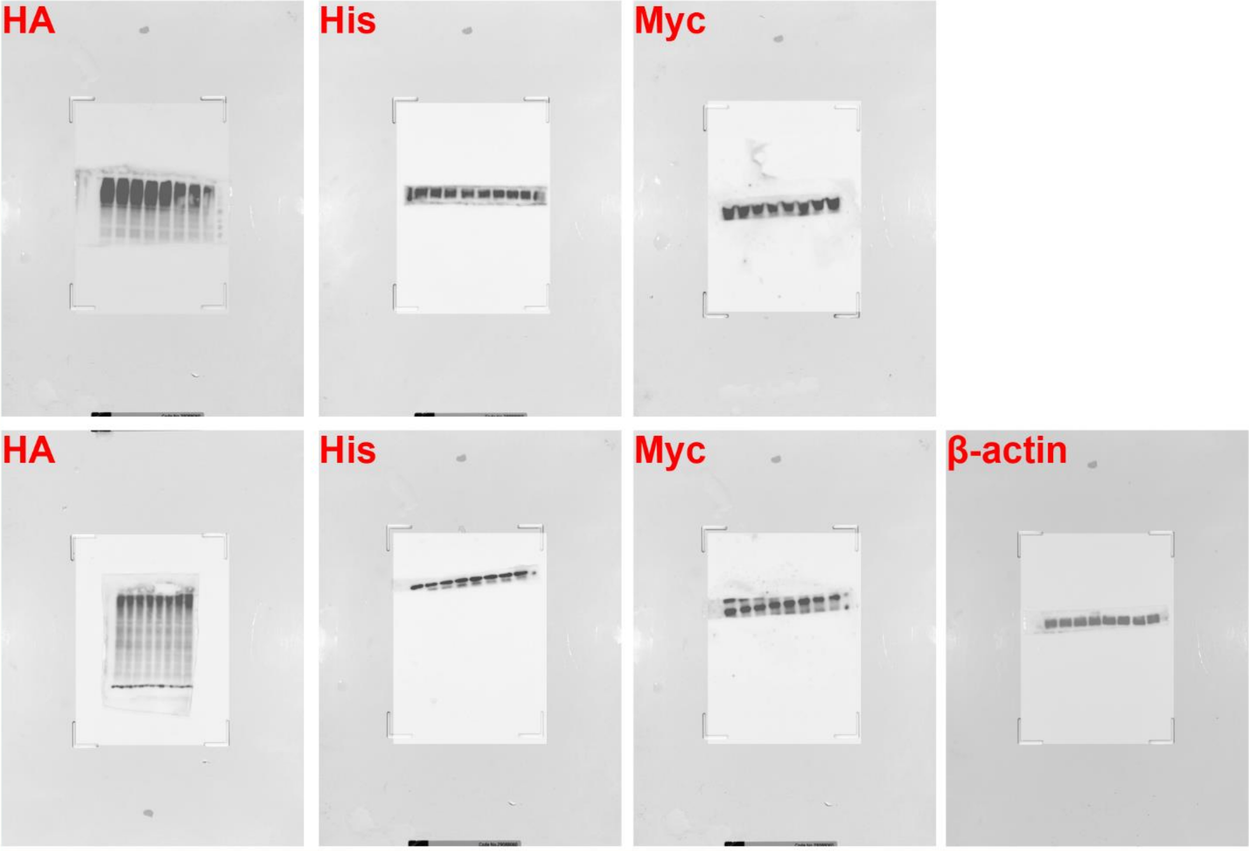

Fig. 5X

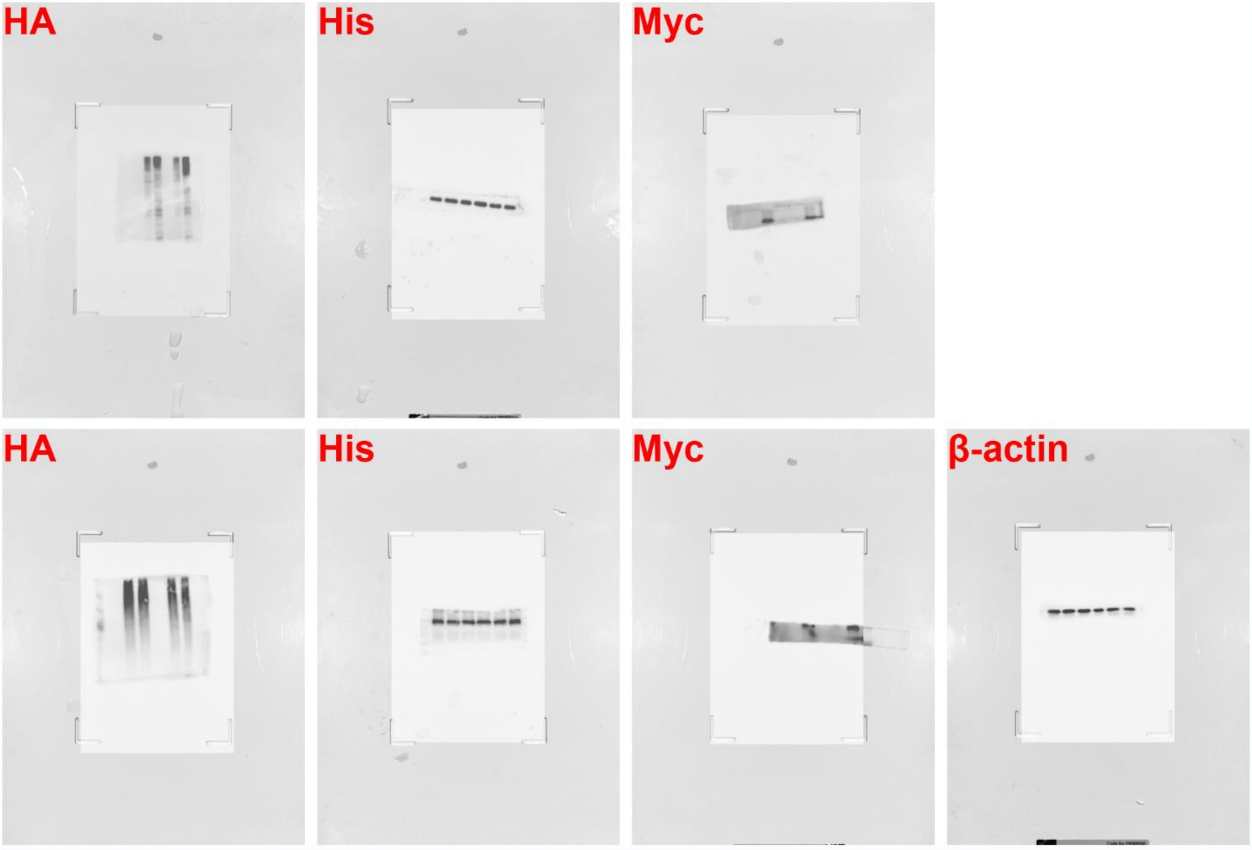

Fig. 5X

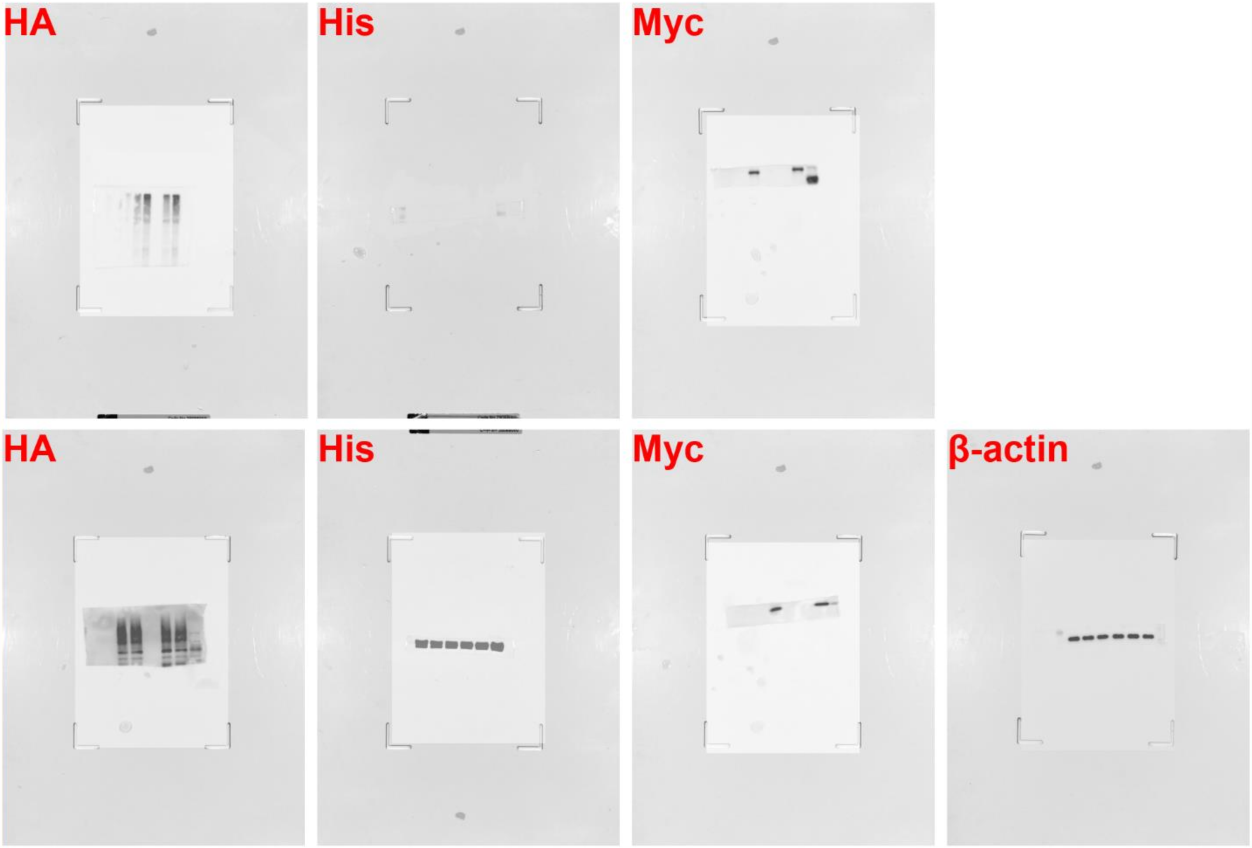

Fig. 5X

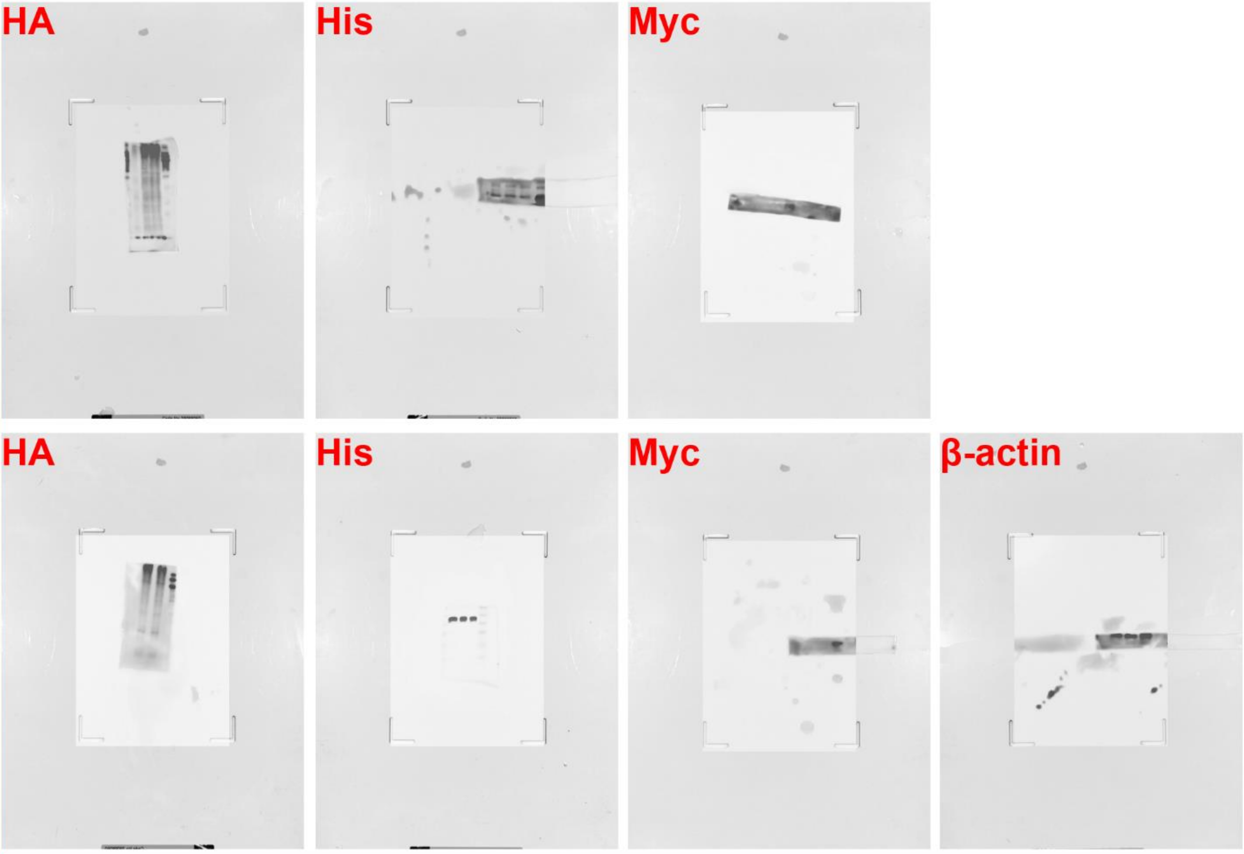

Fig. 6B

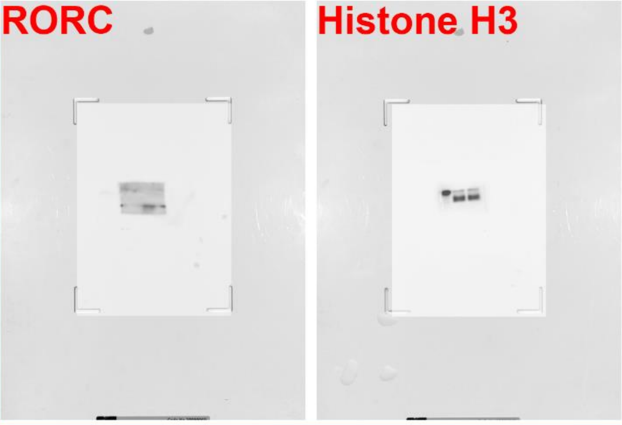

Fig. 6D

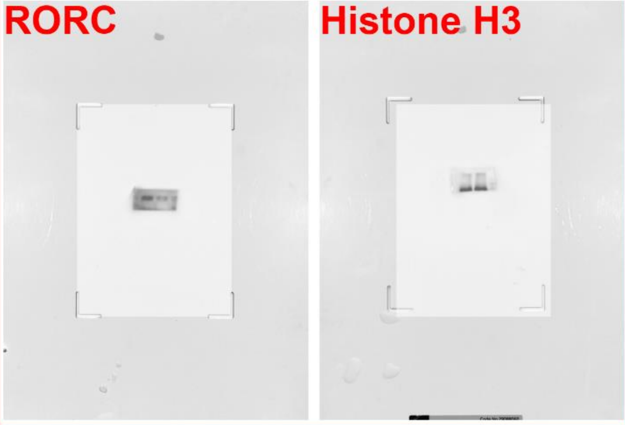

Fig. 6F

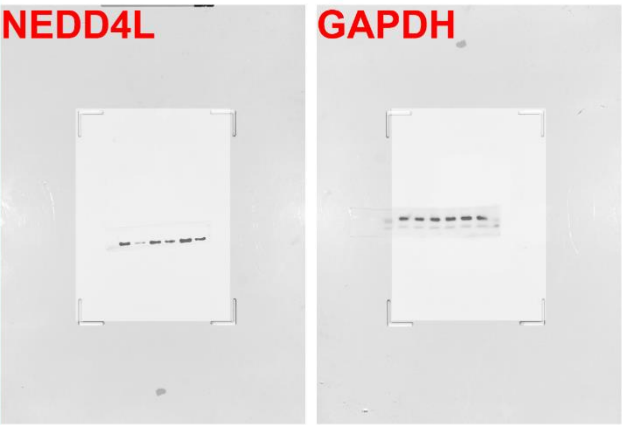

Fig. 6G

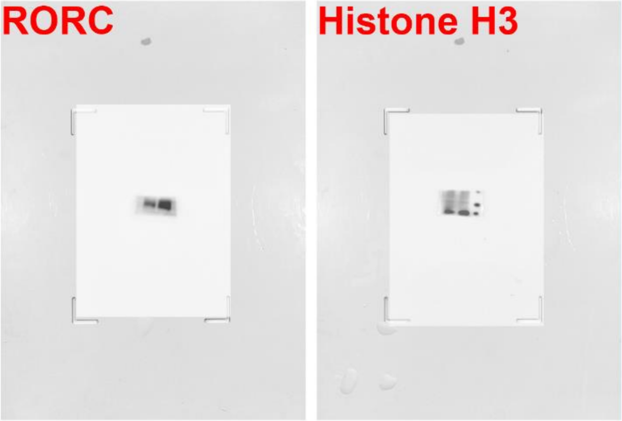

Fig. 6N

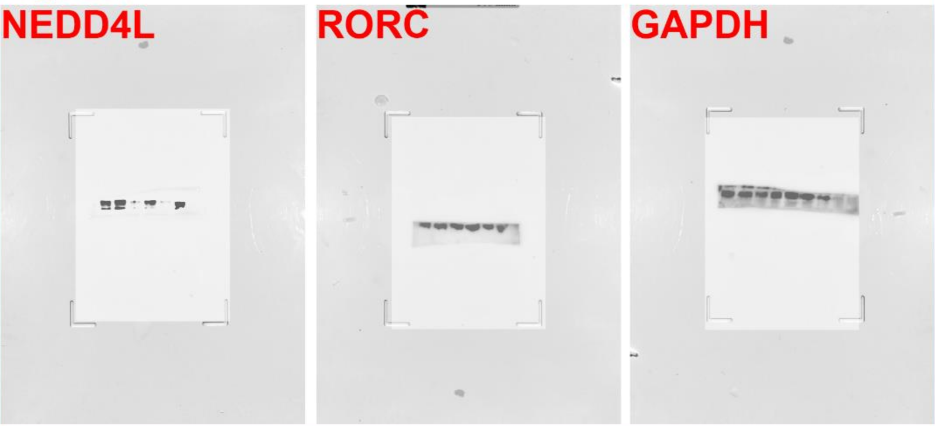

Fig. 6O

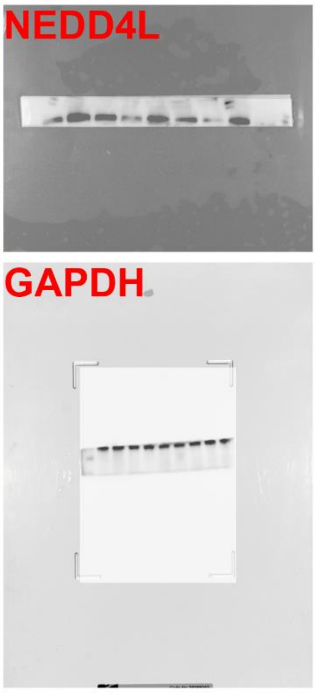

Fig. 6P

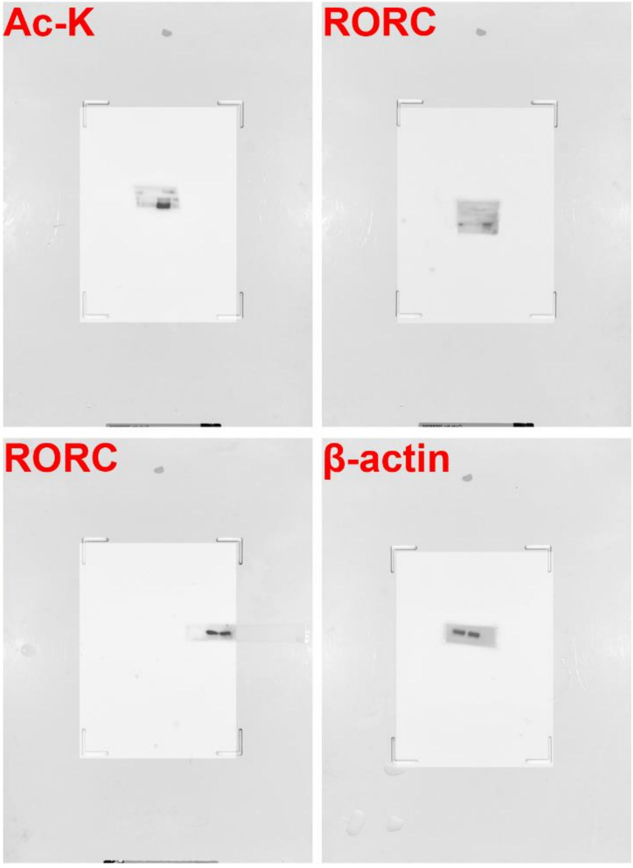

Fig. 6Q

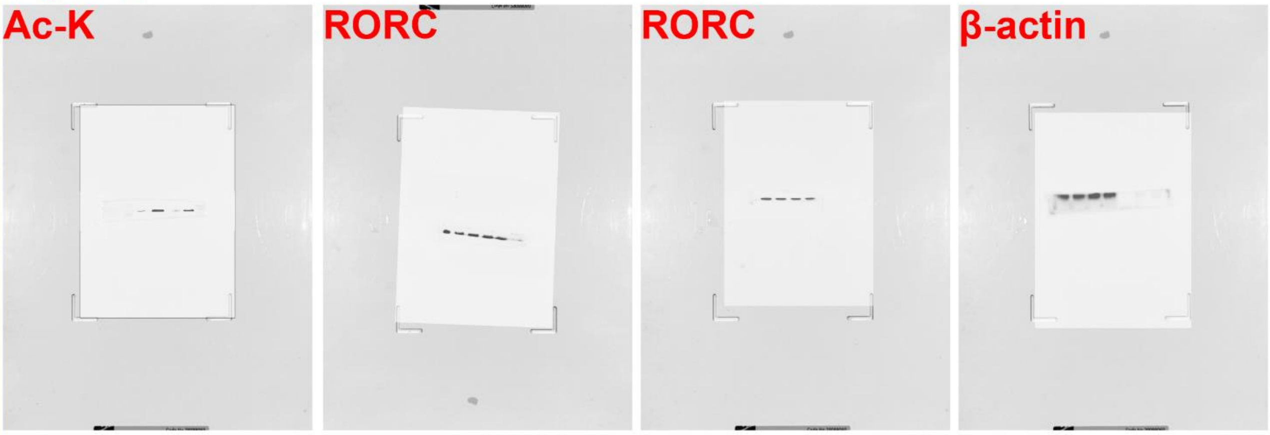

Fig. 6R

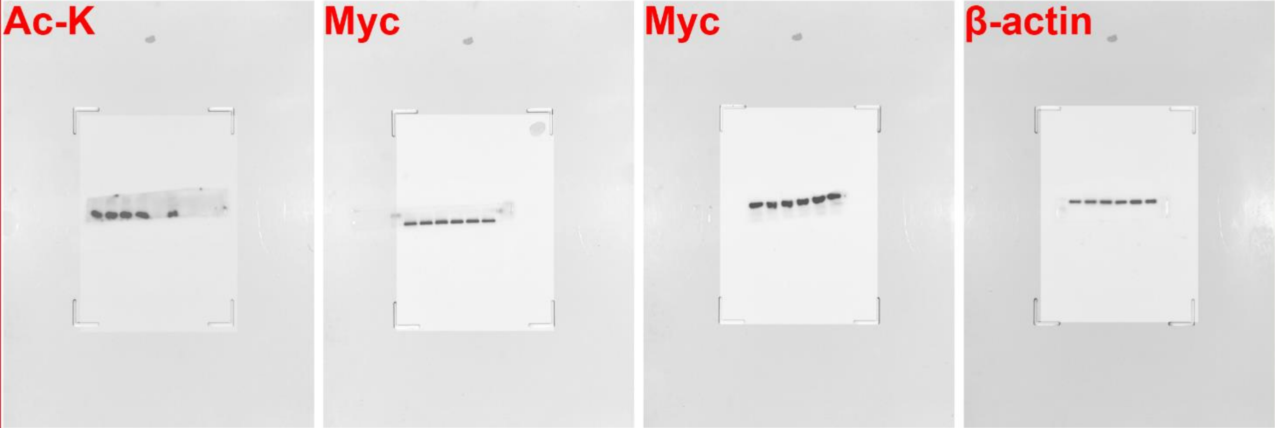

Fig. 6T

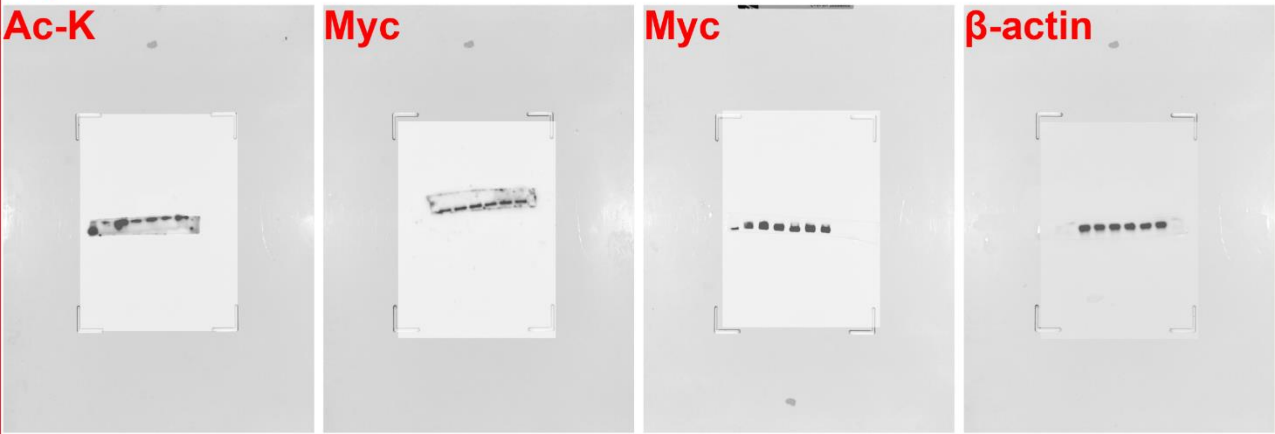

Fig. 6U

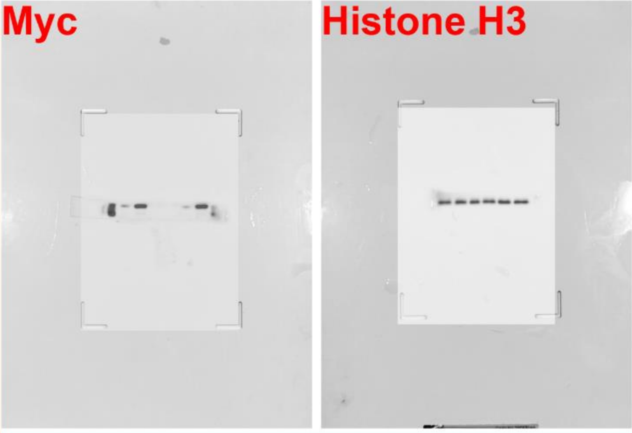

Fig. 7B

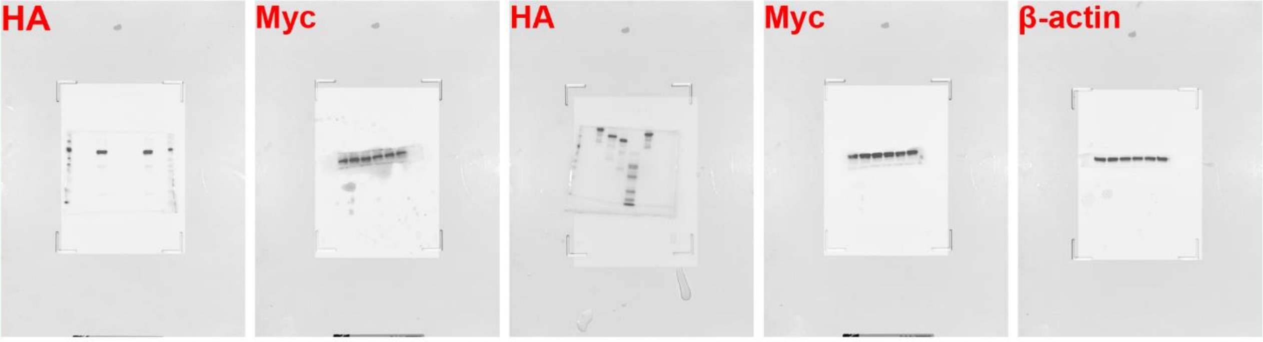

Fig. 7C

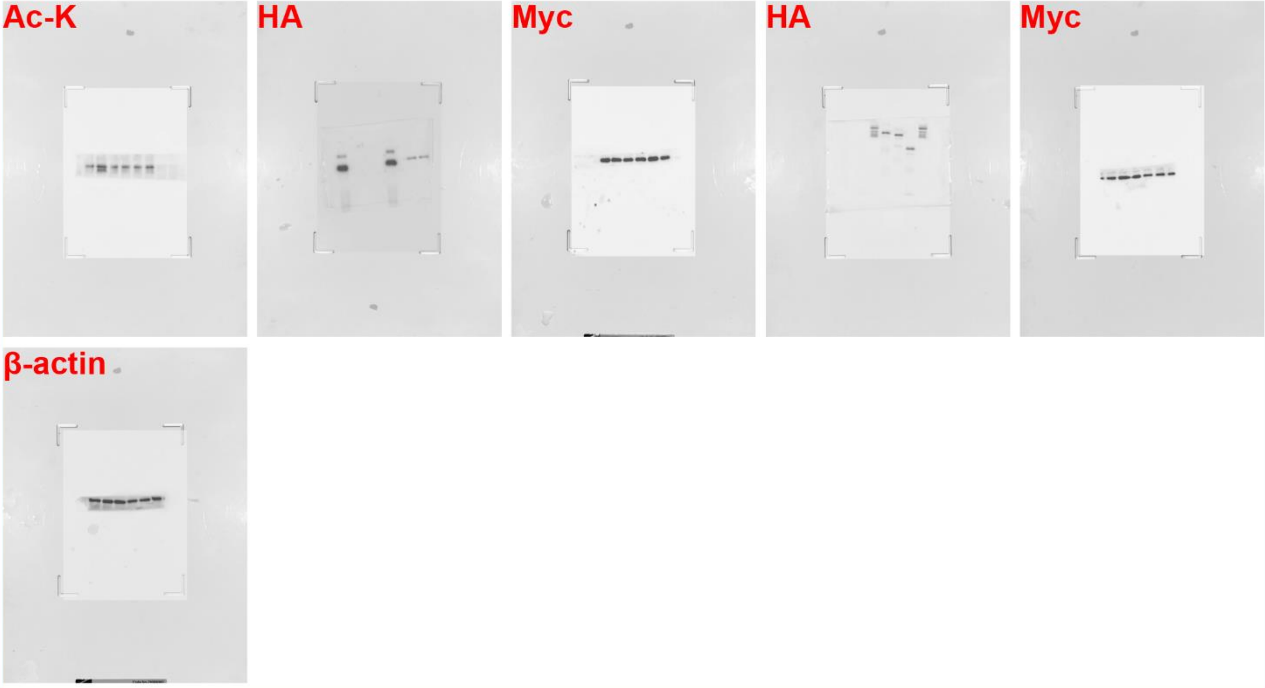

Fig. 7D

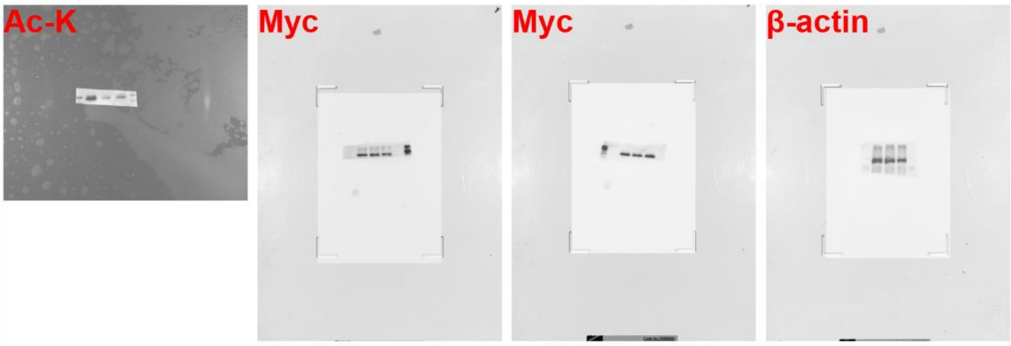

Fig. 7E

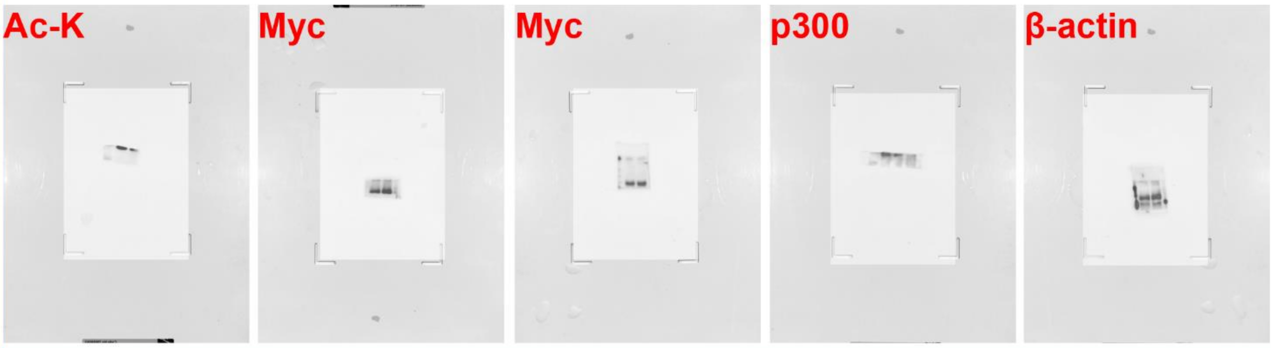

Fig. 7F

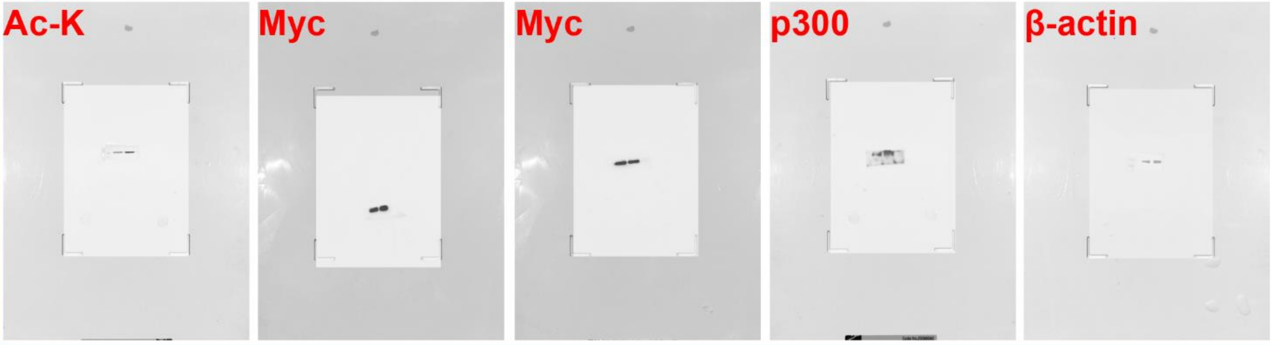

Fig. 7G

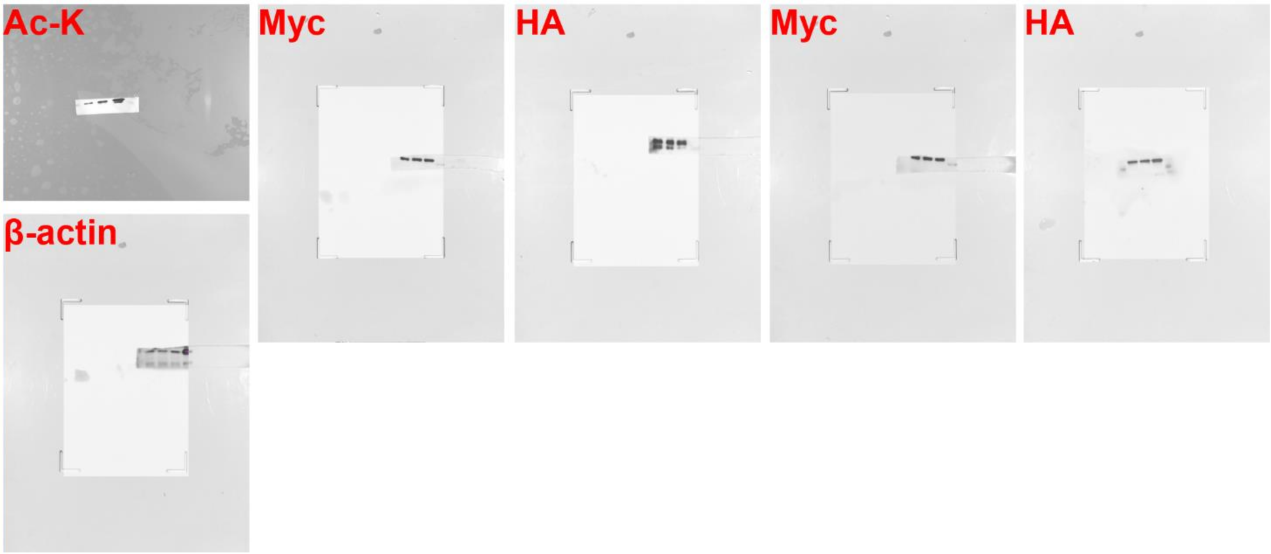

Fig. 7H

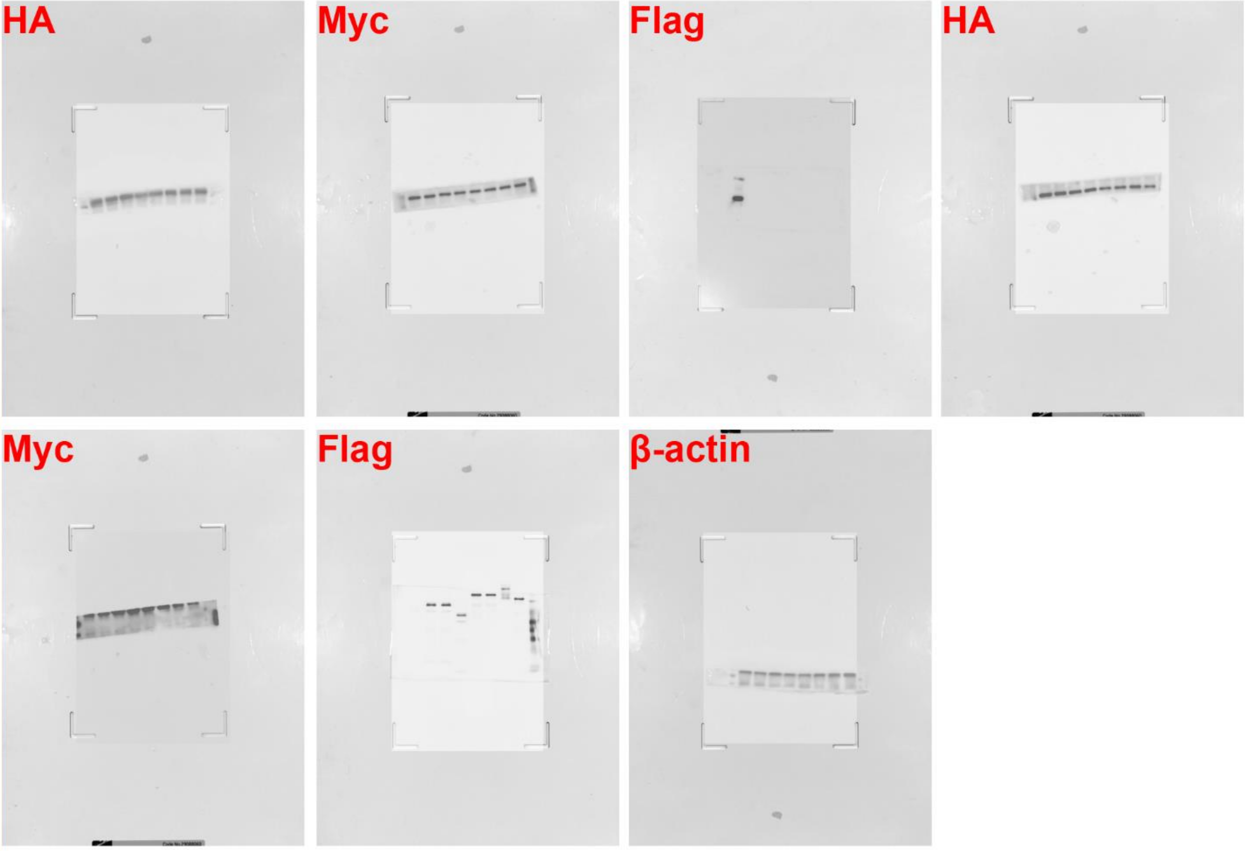
HA
Myc
Flag
HA
Myc
Flag
β-actin

Fig. 7I

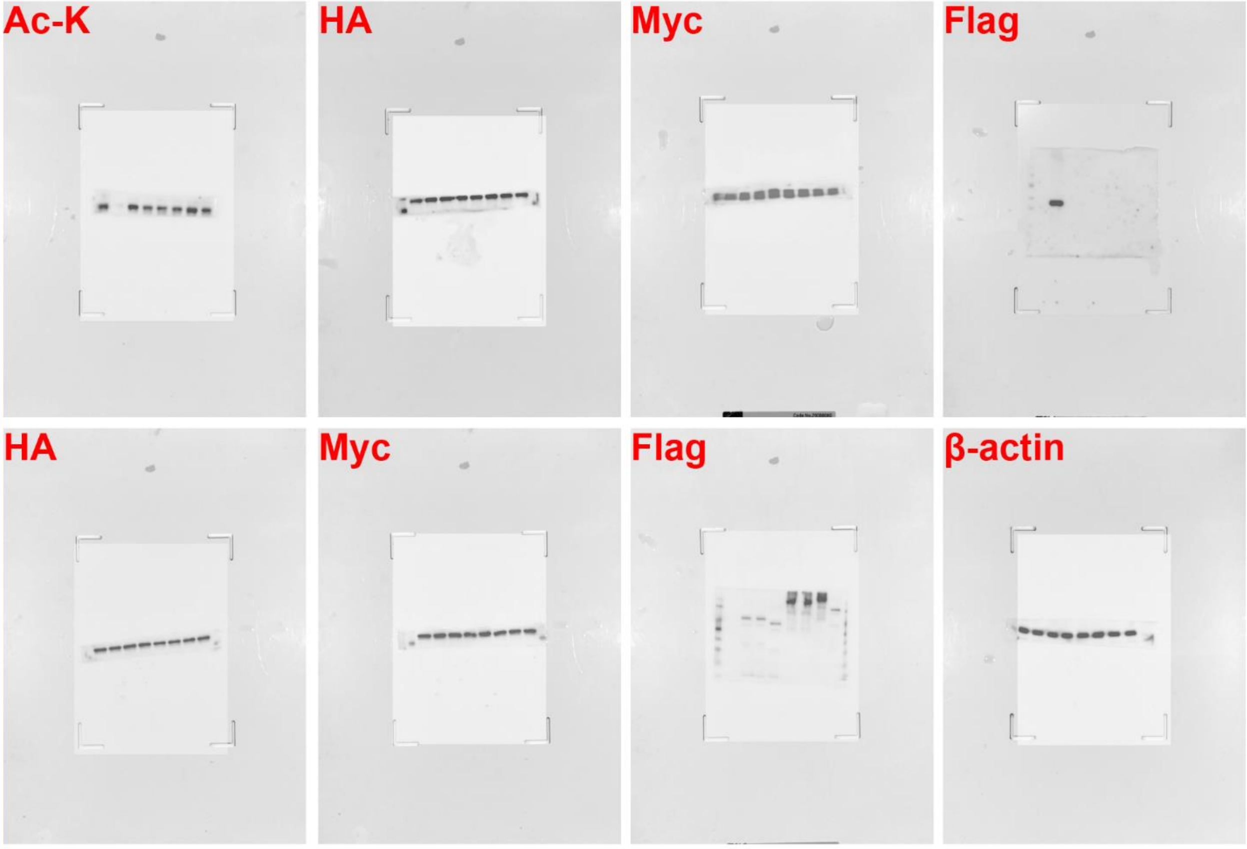
Ac-K
HA
Myc
Flag
HA
Myc
Flag
β-actin

Fig. 7J

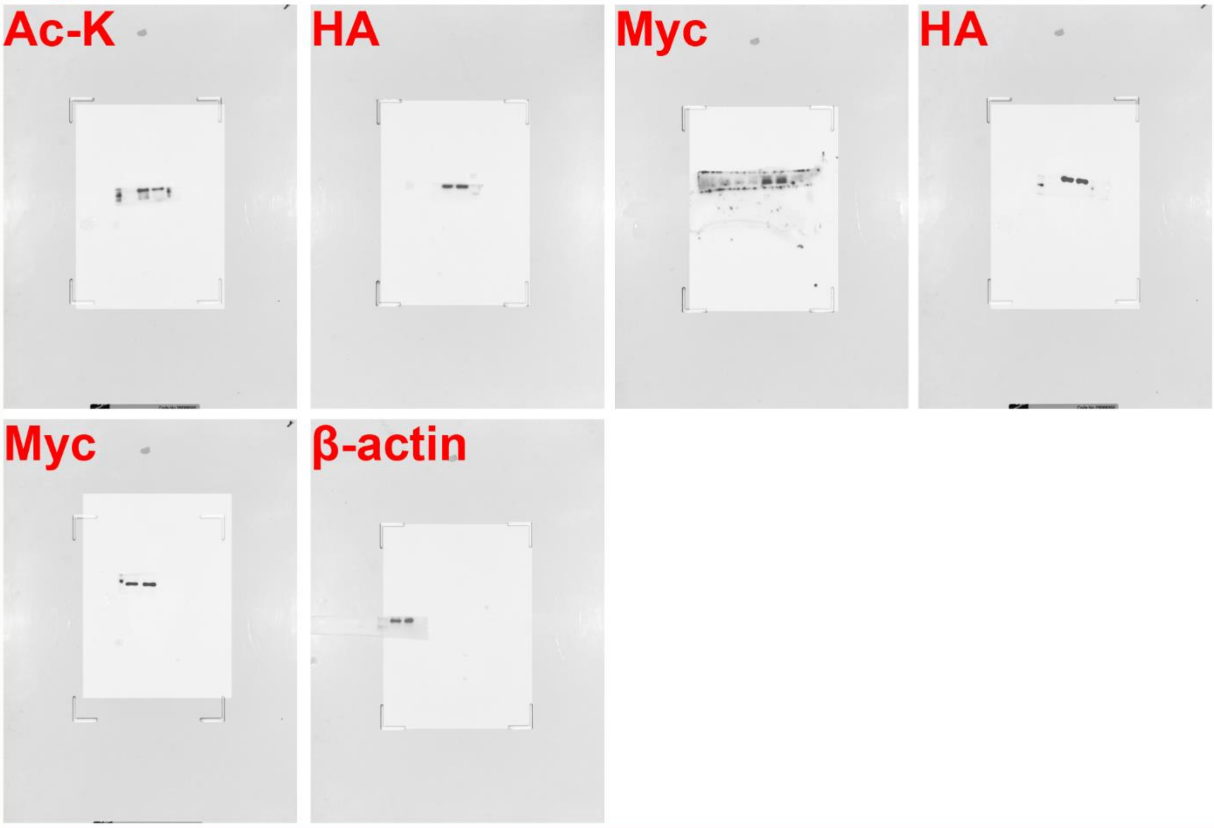

Fig. 7K

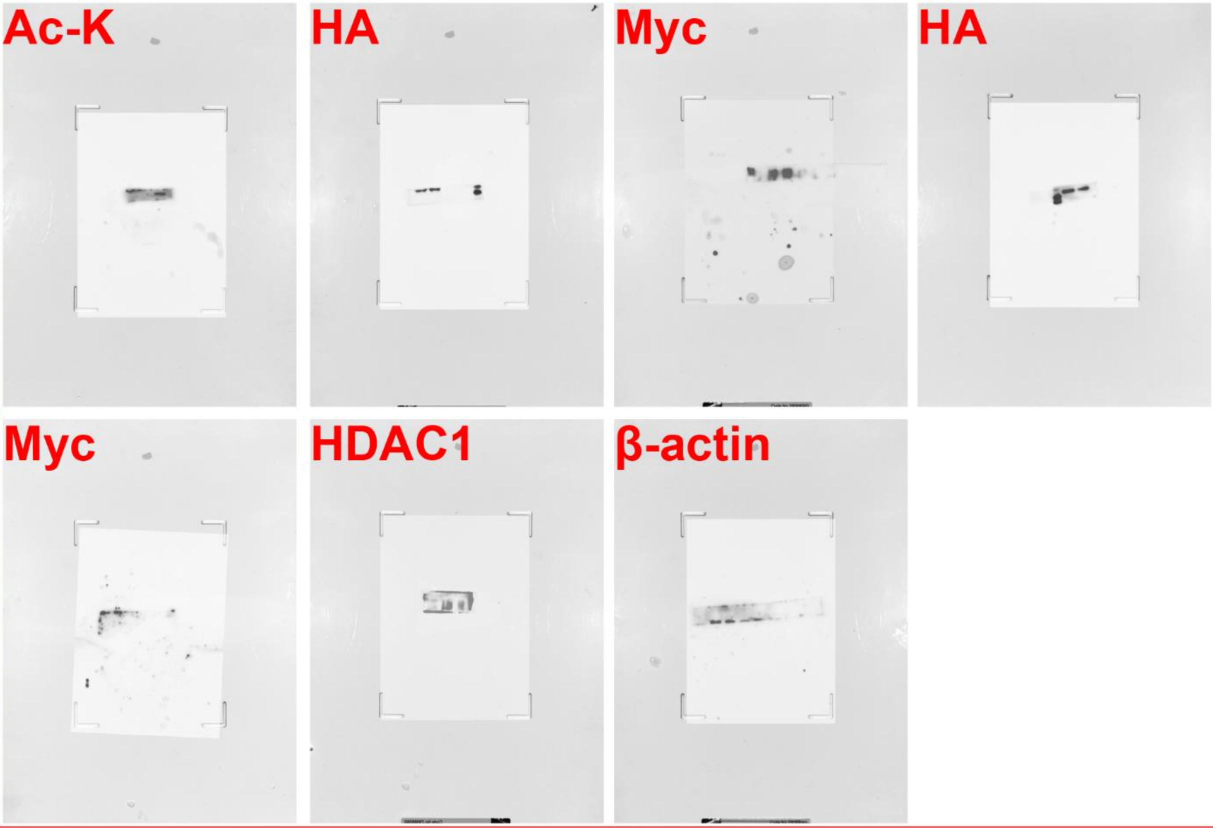

Fig. 7L

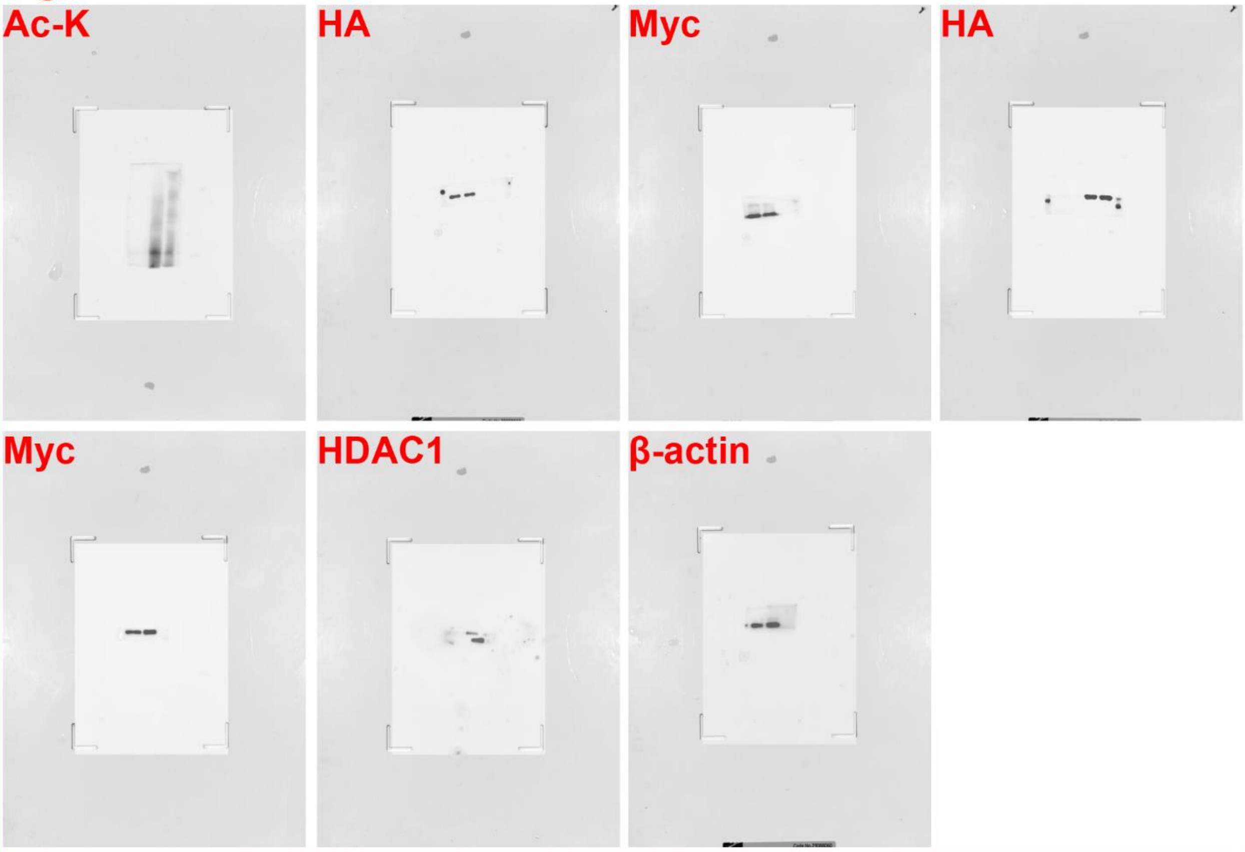

Fig. 7M

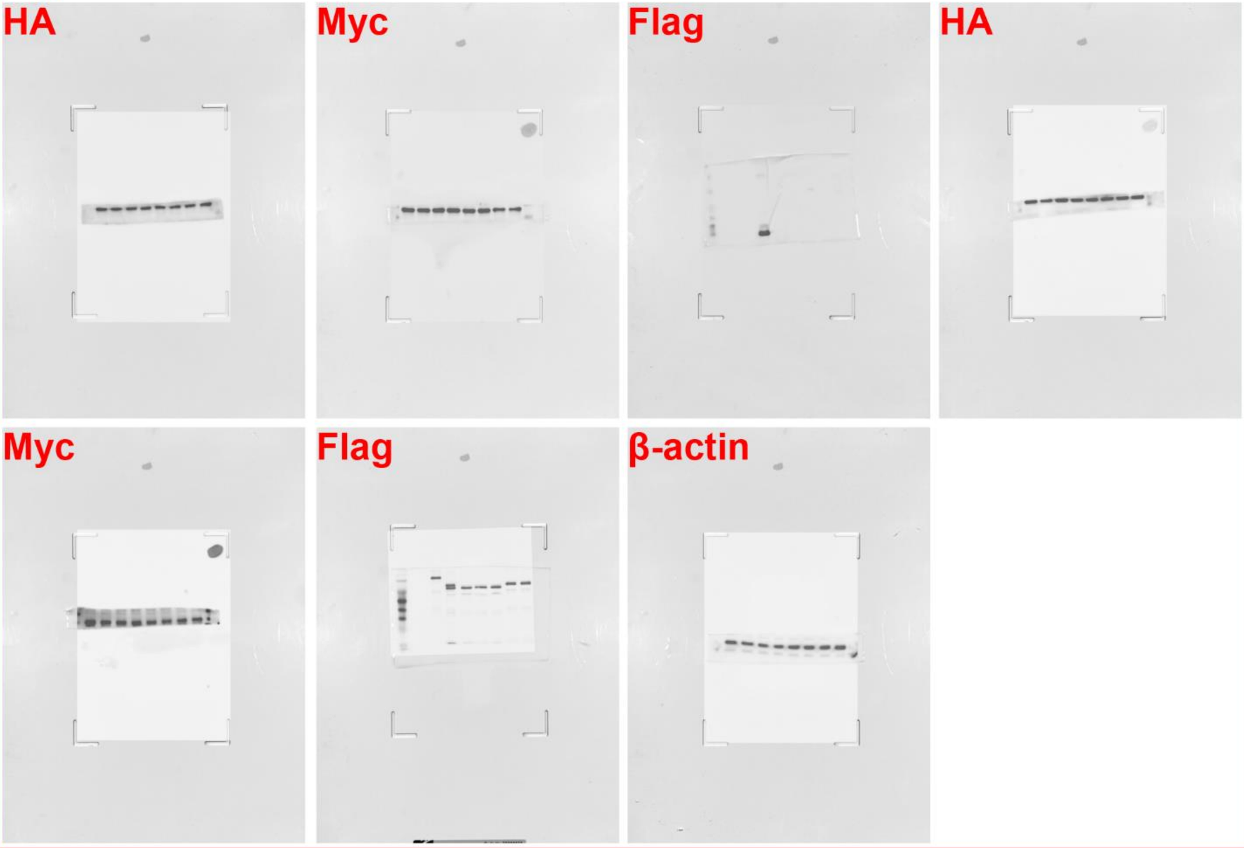

Fig. 7N

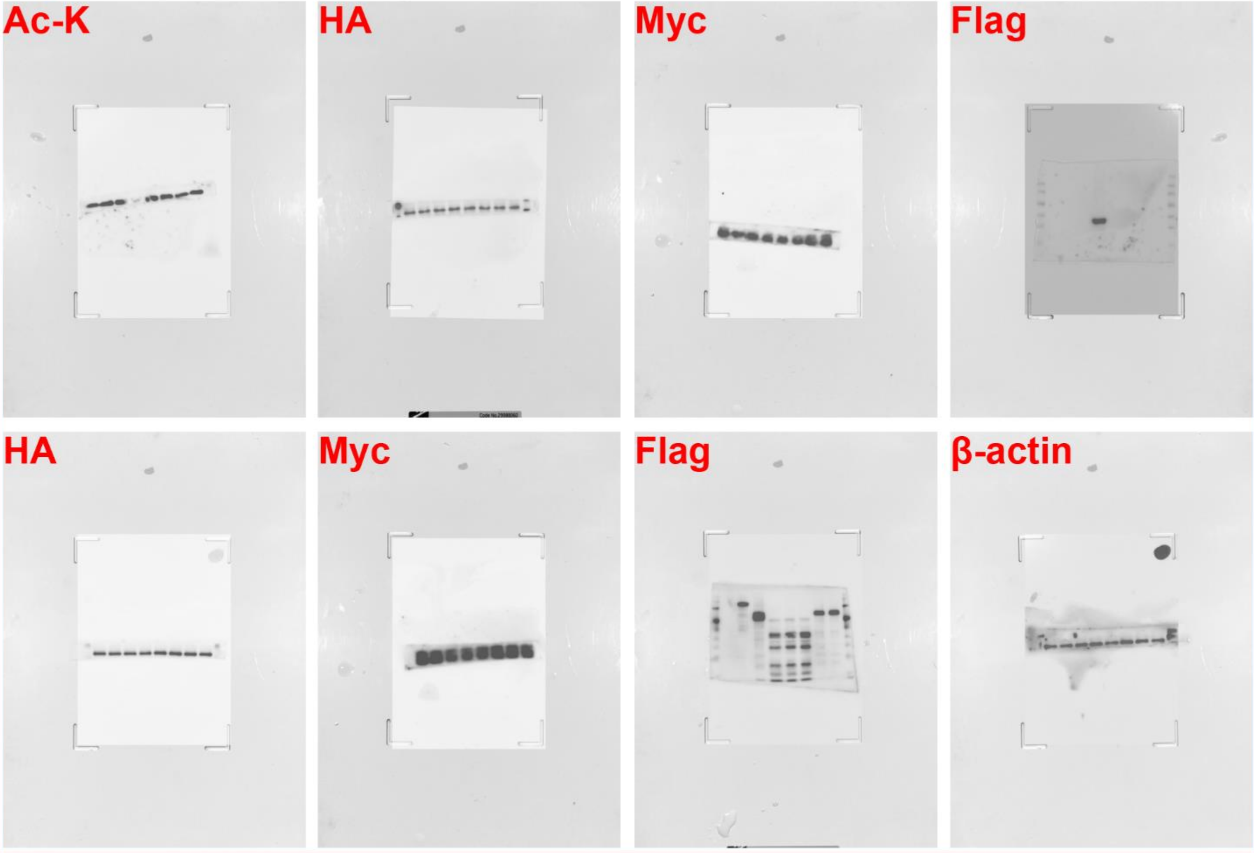

Fig. 7O

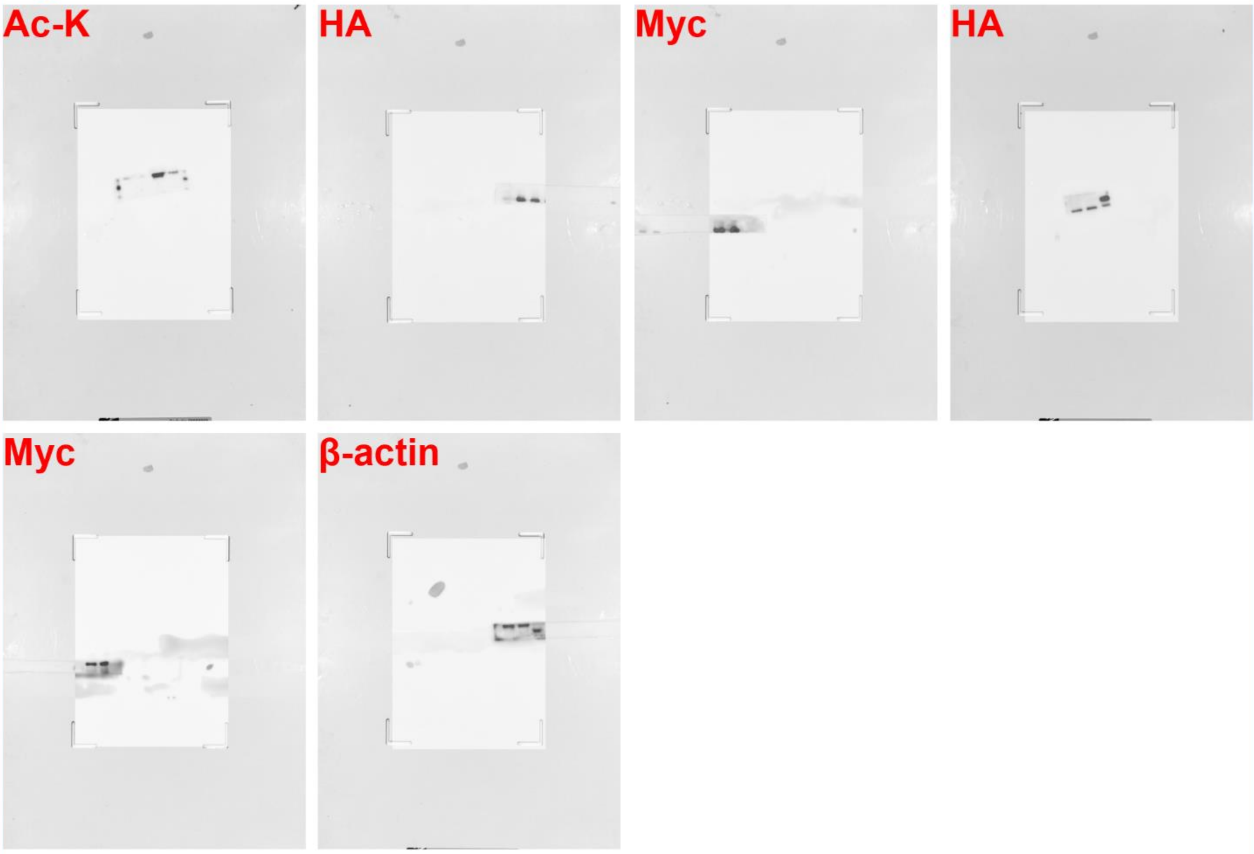

Fig. 7P

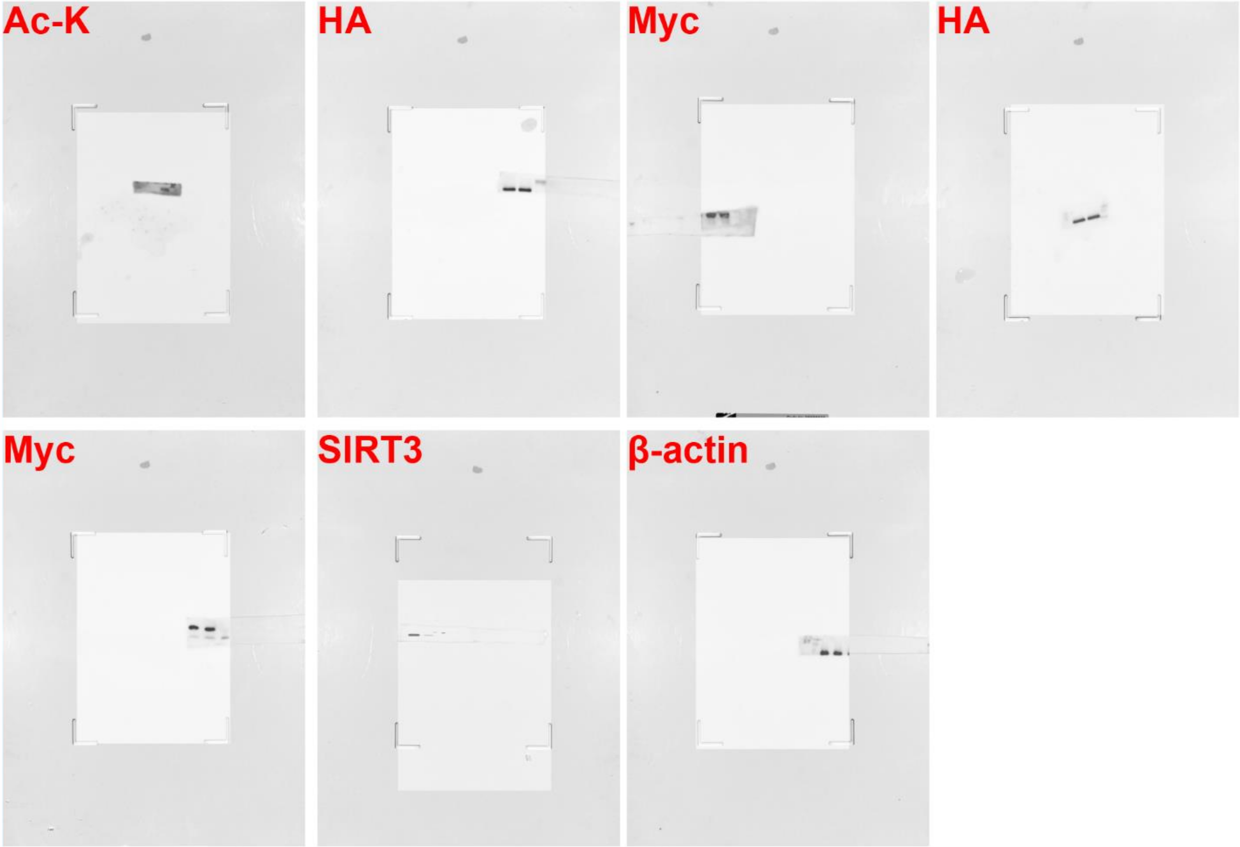

Fig. 7Q

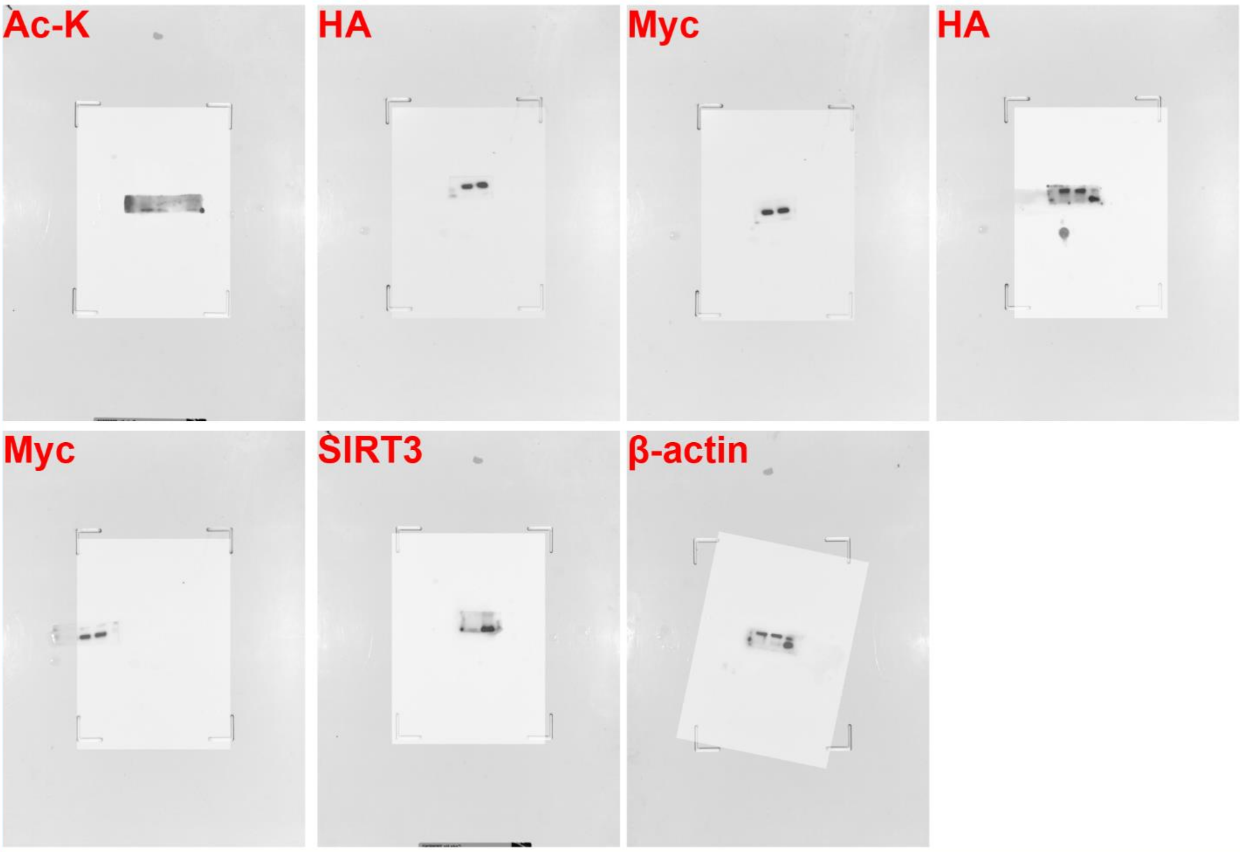

Supplement: Supplementary file 2 — Original western blots [file 41419_2025_7733_MOESM2_ESM.pdf]
